# Non-targeted and chiral amino acid metabolomics of colon cancer: Revealing novel chiral biomarkers and metabolic pathways

**DOI:** 10.1016/j.jpha.2025.101429

**Published:** 2025-08-09

**Authors:** Yuxuan Li, Xinxin Kong, Guangyi Zhang, Hongzhu Jin, Xi-Ling Li, Toufeng Jin, Jun Zhe Min

**Affiliations:** Department of Pharmaceutical Analysis, College of Pharmacy of Yanbian University, Key Laboratory of Natural Medicines of the Changbai Mountain, Ministry of Education, and Department of General Surgery Yanbian University Hospital, Yanji, Jilin, 133002, China

## Abstract

• Non-targeted and DL-amino acid metabolomics employed for biomarker screening in CRC.
• A novel chiral mass spectrometry probe, TPP-BSA, was developed.
• Simultaneous separation of 13 chiral amino acids achieved using a C18 column.
• First-time revelation of the correlation between serum D-amino acids and CRC.
• Characterization of pathways potentially contributing to the development of CRC.

## Graphical abstract

**Figure:**
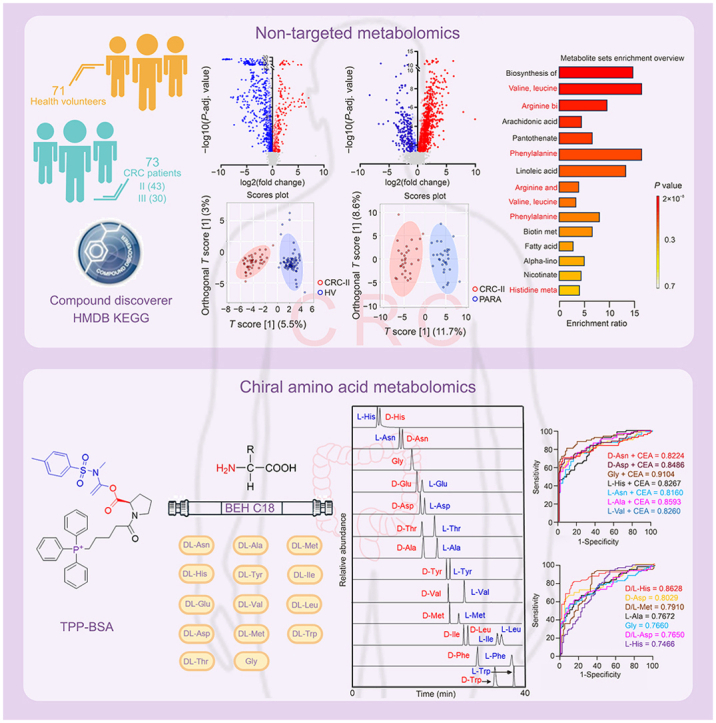

Colorectal cancer (CRC), the third most commonly diagnosed malignancy worldwide, is associated with a high mortality rate [1]. Current blood-based detection of carcinoembryonic antigen (CEA) lacks sufficient sensitivity and specificity for early CRC diagnosis, often resulting in detection at advanced stages [2]. Metabolomics-driven differential metabolite profiling offers insights into CRC pathogenesis and may facilitate the discovery of novel clinical biomarkers [3]. However, the potential significance of chiral compounds in disease development is frequently neglected due to methodological constraints and limitations in existing databases. Accumulating evidence suggests that certain D-enantiomers present in the human body are strongly linked to various pathological conditions [4,5]. In this study, untargeted metabolomics was employed to identify and characterize differential metabolic pathways in serum and tissue samples from patients with CRC. For the first time, the association between serum D-amino acid levels and CRC was elucidated using a newly developed chiral mass spectrometry probe, (S)-(5-(2-(((1-((N,4-dimethylphenyl) sulfonamido) vinyl) oxy) carbonyl) pyrrolidin-1-yl)-5-oxopentyl) triphenylphosphonium (TPP-BSA), which enhanced the resolution of metabolically relevant pathways implicated in CRC pathogenesis.

Untargeted metabolomic profiling was conducted on serum, tumor, and paracancerous tissues obtained from patients with CRC, with clinical information detailed in Table S1. The serum of health volunteers (HVs) and the samples of CRC patients were provided by the physical examination and gastrointestinal surgery of the Affiliated Hospital of Yanbian University (Jilin, China), respectively. This study was conducted by the Declaration of Helsinki. Participants provided written informed consent forms. This research received approval from the Medical Ethics Committee of Yanbian University and the Affiliated Hospital (Approval No.: 2023211). Based on Human Metabolome Database (HMDB) and Kyoto Encyclopedia of Genes and Genomes (KEGG) databases, 19 and 23 significantly altered metabolites were identified in the sera of patients with stage II and stage III CRC, respectively (Table S2). In stage II and stage III patients, 42 and 49 differential metabolites were detected in tumor and paracancerous tissues, respectively (Table S3). Orthogonal Partial Least Squares Discriminant Analysis (OPLS-DA) modeling demonstrated clear separation between HVs and patients with CRC based on these differential metabolites (Figs. 1A and B, S1 and S2). Integrated analysis of serum and tissue metabolomic data highlighted pathways related to amino acid metabolism, unsaturated fatty acid biosynthesis, and arachidonic acid metabolism as key contributors to CRC pathogenesis (Fig. 1C).

**Fig. 1:**
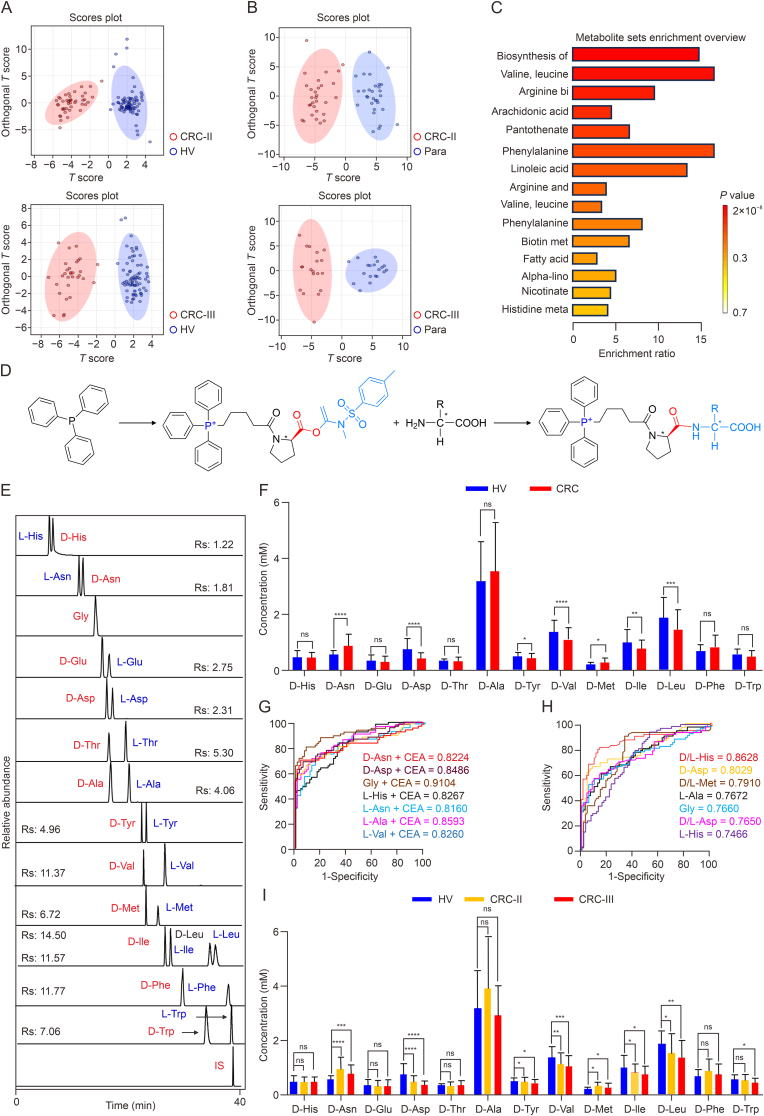
Untargeted metabolomics and (S)-(5-(2-(((1-((N,4-dimethylphenyl) sulfonamido) vinyl) oxy) carbonyl) pyrrolidin-1-yl)-5-oxopentyl) triphenylphosphonium (TPP-BSA)-based targeted amino acid metabolomics. (A) Orthogonal Partial Least Squares Discriminant Analysis (OPLS-DA) of serum differential metabolites in healthy volunteers and colon cancer patients (positive ion mode). (B) OPLS-DA maps of differential metabolites in cancerous and paracancerous tissues of colon cancer patients (positive ion mode). (C) Correlation pathway maps of differential metabolite enrichment in serum and tissues. (D) Synthesis of novel chiral mass spectrometry probes, TPP-BSA, and structural formulae of reaction with DL-amino acids. (E) Evaluation of the efficacy of chiral splitting of DL-amino acids by TPP-BSA. (F) Differences in D-amino acid content in serum of healthy volunteers and colon cancer patients. (G) Receiver Operating Characteristic (ROC) curves of differential amino acid and carcinoembryonic antigen co-diagnosis. (H) ROC curves of DL-amino acids and D/L-amino acid ratios. (I) Differences in D-amino acid content in serum of patients with different staged colon cancer. (^∗^*P* < 0.05, ^∗∗^*P* < 0.01, ^∗∗∗^*P* < 0.001, ^∗∗∗∗^*P* < 0.0001, ns: no significance. HV: Health volunteers. CRC: Colorectal cancer patients. Para: para-cancerous tissue. His: Histidine. Asn: Asparaginate. Gly: Glycine. Glu: Glutamic acid. Asp: Aspartic acid. Thr: Threonine. Ala: Alanine. Tyr: Tyrosine. Val: Valine. Met: Methionine. Ile: Isoleucine. Leu: Leucine. Phe: Phenylalanine. Trp: Tryptophan. IS: Internal standard. CEA: Carcinoembryonic antigen).

To further investigate the role of D- and L-amino acids within these metabolic pathways, the novel chiral probe TPP-BSA—featuring triphenylphosphine as its nucleus—was synthesized and applied (Figs. 1D and S3, Table S4). This enabled simultaneous resolution of 13 chiral amino acids (Fig. 1E, and Table S5). Additionally, a quantitative method was developed and validated to concurrently measure 14 amino acids in human serum, with methodological validation summarized in Tables S6–S10.

DL-amino acid content was analyzed in the sera of 70 HVs and 68 patients with CRC (Tables S11–S14). Significant alterations in amino acid levels were observed, with L-histidine (His) (*P* < 0.0001), glycine (Gly) (*P* < 0.0001), L-alanine (Ala) (*P* < 0.0001), L-tyrosine (Tyr) (*P* < 0.001), L-valine (Val) (*P* < 0.0001), L-leucine (Leu) (*P* < 0.001), D-asparaginate (Asn) (*P* < 0.0001), and D-methionine (Met) (*P* < 0.05) exhibiting upregulation, while L-Asn (*P* < 0.0001), D-aspartic acid (Asp) (*P* < 0.0001), D-Tyr (*P* < 0.05), D-Val (*P* < 0.0001), D-isoleucine (Ile) (*P* < 0.01), and D-Leu (*P* < 0.001) showed a downregulation trend (Figs. 1F, S5A, and S5B). To enhance CRC detection using amino acid markers, the differential amino acids were combined with CEA, revealing area under curve (AUC) values greater than 0.8 for L-His, L-Asn, L-Ala, L-Val, D-Asn, and D-Asp, indicating a strong correlation with CRC. Notably, the AUC for Gly combined with CEA was 0.9104 (Fig. 1G).

Further analysis of the DL-amino acid ratios revealed significant differences between CRC and HV groups, with upregulated ratios for His (*P* < 0.0001), Asn (*P* < 0.05), Ala (*P* < 0.0001), Tyr (*P* < 0.01), Val (*P* < 0.001), Met (*P* < 0.0001), and Trp (*P* < 0.01) in CRC, while Asp (*P* < 0.0001) and Ile (*P* < 0.05) ratios were downregulated (Fig. S5C). Receiver Operating Characteristic (ROC) curve analysis for D-amino acids, L-amino acids, and DL-amino acid ratios were plotted and an attempt was made to differentiate between HV and CRC using the OPLS-DA model. However, complete separation could not be achieved, although there was a certain trend of separation between HV and CRC (Figs. 1H and S5D). The identified differential amino acids suggest that CRC pathogenesis may be associated with metabolic pathways including alanine, aspartate, and glutamate metabolism, histidine metabolism, the glyoxylate cycle, and glutathione metabolism (Fig. S5E). To further elucidate the metabolic pathways implicated in CRC, untargeted metabolomics and DL-amino acid metabolomics data were integrated and mapped using ipath 3.0 software, providing valuable insights into CRC pathogenesis and potential therapeutic targets (Fig. S6).

Patients with CRC are clinically staged based on pathological findings. Therefore, serum levels of D-amino acids, L-amino acids, and D/L-amino acid ratios were analyzed across different CRC stages to determine their correlation with disease progression ( Figs. 1I and S7). The levels of D-Val, L-Val, and D-Leu exhibited increased significance as CRC advanced. While L-Trp and D-Trp showed no significant differences in stage II serum, they demonstrated significant downregulation in stage III patients. These amino acids warrant further investigation in subsequent studies.

This study identified significantly altered metabolites in the serum of HVs and patients with CRC, as well as in cancer and paracancer tissues from patients with CRC, using untargeted metabolomics. The metabolic pathways potentially contributing to CRC pathogenesis were subsequently explored. Given the strong association between amino acid synthesis and metabolism with CRC, a novel chiral mass spectrometry probe, TPP-BSA, was developed for the separation and quantification of DL-amino acids in the serum of patients with CRC. For the first time, the correlation between specific D-amino acids and CRC was established using this newly developed derivatization reagent, refining the understanding of the related metabolic pathways that may drive CRC pathogenesis. These findings offer valuable insights into CRC pathogenesis and present potential biomarkers for clinical diagnosis of CRC.

## CRediT authorship contribution statement

**Yuxuan Li:** Writing – review & editing, Investigation. **Xinxin Kong:** Writing – review & editing, Methodology. **Guangyi Zhang:** Methodology. **Hongzhu Jin:** Validation. **Xi-Ling Li:** Formal analysis, Data curation. **Toufeng Jin:** Conceptualization. **Jun Zhe Min:** Writing – review & editing, Project administration, Funding acquisition.

## Declaration of competing interest

The authors declare that there are no conflicts of interest.
