# Supplementary material for: Non-targeted and chiral amino acid metabolomics of colon cancer: Revealing novel chiral biomarkers and metabolic pathways
Source: J Pharm Anal. 2025 Aug 9;16(1):101429. doi: 10.1016/j.jpha.2025.101429 (PMC12874408; doi:10.1016/j.jpha.2025.101429)
Supplement: Multimedia component 1 [file mmc1.docx]

**1. Introduction**

Recently, Ultra-high-performance liquid chromatography high-resolution mass spectrometry (UHPLC-HRMS)-based metabolomics has emerged as an essential instrument in systems biology, playing a critical role in understanding the interactions between small molecule metabolites and diseases such as cancer [1]. However, due to limitations in databases and detection technologies, the identification of chiral compounds through conventional untargeted metabolomics alone remains challenging, necessitating the use of chiral-targeted and chiral-untargeted metabolomics for validation [2,3]. Chiral targeted metabolomics not only enables effective separation of chiral compounds but also allows for the determination of their absolute concentrations in biological samples from healthy and diseased groups using absolute quantification methods, which is of significant importance for the development of clinical biomarkers [4]. Colorectal cancer (CRC), characterized by an extremely high mortality rate, has been a major focus of research, particularly in the areas of early diagnosis and targeted treatment [5]. Despite this, the relationship between CRC and the levels of chiral compounds, as well as associated metabolic pathways, remains poorly understood. This research utilized both non-targeted and chiral-targeted metabolomics to thoroughly analyze metabolic changes in the serum of patients with CRC, shedding light on the role of chiral amino acids (AAs) and other key metabolites in CRC pathogenesis. The findings present essential theoretical insights and identify possible biomarkers for the clinical management and diagnosis of CRC.

**2. Materials and methods**

*2.1. Reagents*

Triphenylphosphine (TPP), DL-valine (Val), D/L-aspartic acid (Asp), and D/L-histidine (His) were obtained from TCI Co., Ltd (Tokyo, Chiba, Japan). 5-bromovaleric acid, sodium carbonate, N, N-diisopropylethylamine (DIPEA), anhydrous sodium sulfate, dichloromethane (DCM), Ammonium acetate, and (S) - 3-amino-3 - (4-bromophenyl) propionic acid (IS) were from Aladdin Biochemical Technology Co., Ltd (Shanghai, China). D-proline tert-butyl ester, N, N, N', N'- tetramethylchloroformamidine hexafluorophosphate, N-methylimidazole (NMI), and N-ethynyl-N,4-dimethylbenzenesulfonamide were obtained from Bide Pharmaceutical Technology Co., Ltd. (Shanghai, China). Hydrochloric acid, D/L-alanine (Ala), D/L-phenylalanine (Phe), D/L-asparagine (Asn), D/L-tryptophan (Try), D/L-methionine (Met), DL-isoleucine (Ile), D/L-leucine (Leu), D/L -glutamic acid (Glu) and glycine (Gly) were provided by Budweiser Reagent Co., Ltd (Shanghai, China). Ethyl acetate was purchased from Quanrui Reagent Co., Ltd (Dalian, Liaoning, China). D/L-tyrosine (Tyr) and D/L-threonine (Thr) were provided by Sigma Aldrich (Shanghai, China). Artificial serum was provided by Huizhi Heyuan Biotechnology Co., Ltd (Suzhou, Jiangsu, China). LC-MS grade acetonitrile (ACN), methanol (MeOH), and formic acid were purchased from Fisher Scientific (Pittsburgh, Pennsylvania, USA).

*2.2 CRC and HV samples and ethical statement*

The serum of HVs and the samples of CRC patients were provided by the physical examination and gastrointestinal surgery of the Affiliated Hospital of Yanbian University (Jilin, China), respectively. This study included 73 patients with CRC (47 males aged 39-85 and 26 females aged 37-84) were collected in this study. The pathological diagnosis was made by experts in the Department of Pathology of the Affiliated Hospital of Yanbian University. There were 43 patients with stage-II CRC and 30 patients with stage-III CRC (Table S1). Healthy human serum samples were provided by 71 volunteers without intestinal diseases (33 males 51-83 and 38 females 51-81 years old). This study was conducted by the Declaration of Helsinki. Participants provided written informed consent forms. This research received approval from the Medical Ethics Committee of Yanbian University and the Affiliated Hospital. (Approval No. 2023211, Approval Date February 11, 2023).

*2.3 Synthesis of a novel chiral probe TPP-BSA*

In a 10 mL ACN solution, 1 mol of TPP and 1.2 mol of 5-bromopentanoic acid were dissolved, and the mixture was heated to 85°C on a magnetic stirrer with nitrogen protection for 24 hours. After cooling the reaction liquid to room temperature (Rt), the solvent was extracted with a rotary evaporator to produce crude (4-carboxybutyl) triphenylphosphonium (TPPP). Subsequently, the TPPP was washed three times with ethyl acetate, and the filter residue was retained by filtration to obtain the pure product of TPPP. 1 mol of the TPPP sample was dissolved in 20 mL of ACN, followed by the sequential addition of 1.2 mol of tert-butyl D-proline and 2.2 mol of N-methylimidazole to the above solution. After stirring for 5 minutes at Rt, 1.2 mol of N, N, N',N'-tetramethylchloroauron hexafluorophosphate was added to the above solution and reacted overnight at Rt. After the reaction finished, the solvent was removed by evaporation, and the residue was re-dissolved in 30 mL of dichloromethane. The dichloro layer was washed by water, saturated sodium carbonate solution, and 1 M hydrochloric acid solution, respectively. Anhydrous sodium sulfate was used to dry the retained organic layer, which was then filtered and spin-dried to create the intermediate product(5-(2-(tert-butoxycarbonyl) pyrrolidin-1-yl)-5-oxopentyl) triphenylphosphonium (TPP-D-Pro-OtBu). The intermediate TPP-D-Pro-OtBu was dissolved in 3 mL of ACN, followed by the addition of 10 mL of hydrochloric acid solution (6 M) and stirred overnight to remove the tert-butyl ester group. Then the precipitated sample was filtered and placed in a vacuum drying oven to obtain a pure product of (5-(2-carboxypyrrolidin-1-yl)-5-oxopentyl) triphenylphosphonium (TPP-D-Pro). 1 mol of TPP-D-Pro was mixed with 5 mL of dichloromethane, followed by the addition of 1 mol of N-ethynyl-N,4-dimethylbenzenesulfonamide. The reaction was allowed to proceed overnight at Rt. Finally, the solvent was evaporated to give the crude product (*S*)-(5-(2-(((1-((N,4-dimethylphenyl) sulfonamido) vinyl) oxy) carbonyl) pyrrolidin-1-yl)-5-oxopentyl) triphenylphosphonium (TPP-BSA). The crude TPP-BSA was purified using a reverse ODS column with solvents of water and methanol solution. After purification, the pure product of TPP-BSA was obtained.

*2.4 Amino acids preparation and labeling reaction of TPP-BSA*

Due to the different solubility of AAs, 50 mM of AAs stock solution was prepared using ACN, ACN: H_2_O=1:1 (*v:v*), and 2% HCl solution, respectively. Subsequently, the above stock solutions were diluted and mixed with ACN: H_2_O=1:1 (*v:v*) and artificial serum, respectively, to prepare a mixed solution containing 1 mM of each DL-amino acid. The pure solvent standard solution was used for subsequent derivatization reaction evaluation, while the artificial serum standard solution was used for methodological research. IS was prepared using ACN: H_2_O=1:1 (*v:v*), and the final concentration was 0.5 mM. The 2.0 mL centrifuge tube was added with 50 μL DL-AAs, 50 μL of 0.5 mM IS, 100 μL of 10 mM DIPEA solution, and 100 μL of 10 mM TPP-BSA solution. After thorough mixing, the reaction was placed under a metal heater at 70^o^C for 1 hour for derivatization. Under these reaction conditions, DL-AAs were essentially completely labeled. The reaction solution was allowed to cool and then passed through a 0.22 μm filter membrane for UHPLC-HRMS determination. The optimal fragmentation ions for each AAs were examined in Full MS DDMS^2^ mode.

*2.5 Validation of the method*

Calibration curves were created by spiking different concentration levels of DL-AAs and constant concentrations of IS into artificial serum for the internal standard method. The calibration curve for each analyte consisted of six points, which were measured three times in the corresponding concentration ranges. The signal-to-noise ratios (S/N) were 10:1 and the limits of quantification (LOQ) were determined accordingly. Parallel quality control samples were analyzed for intra- and inter-day precision and accuracy based on the different AAs to be measured. Matrix effects and recoveries were assessed by analyzing six replicates of artificial serum spiked with different concentration levels of DL-AAs. Three different concentrations of spiked artificial serum were placed at Rt for 4 h, stored in an autosampler (4^o^C) for 8 h, and placed in the presence of three freeze-thaw cycles from -20^o^C to Rt, respectively, and relative standard deviation (RSD) values were used to assess the stability of the samples.

*2.6 Biological sample pretreatment*

Transfer 200 μL of serum sample solution into a 2 ml Ep tube, and then add 800 μL of pre-chilled methanol solution, vortex for 30 s, and store at -20 ^o^C for 30 min. Thereafter, the sample was centrifuged at 13000 rpm for 10 minutes to remove the protein, and the supernatant was concentrated in a vacuum concentration system. After adding 200 μL of MeOH: H_2_O =1:1 (*v/v*) to the concentrated sample, vortex for 30 s. Ultimately, the solution was filtered using a 0.22 μm membrane and employed for UHPLC-HRMS measurement. Serum Quality Control (QC) samples were obtained by mixing 10 μL of samples from the HV group and CRC group.

Slice tissue samples (100 mg) and two grinding beads were placed in 1.5 ml Ep tubes. Homogenize in a tissue mill at 60 Hz for 1 minute and repeat the process three times. The homogenate was then sonicated in an ice bath for 30 minutes and the extract was poured into the homogenizing tube. Subsequently, 1200 μL of pre-cooled methanol solution was added and stored at -20^o^C for 30 min. Subsequent precipitation of proteins and re-solubilization methods were consistent with serum samples.

For the targeted DL-AAs study, the serum sample pretreatment was consistent with the previously described procedure, followed by aspiration of 50 μL of the replicated serum sample for derivatization using the procedure in section 2.4, with a final injection volume of 1 μL.

*2.7 UHPLC-HRMS conditions*

The ultra-high performance liquid chromatography system was connected to the quadrupole or orbital ion trap Q Exactive high-resolution mass spectrometer equipped with heated electrospray ionization (HR-ESI) source interface (Thermo Fisher Scientific, Waltham, Massachusetts, USA). CORTECS C18 (2.7 µm, 3 mm×150 mm) was used for the separation of substances in untargeted metabolomics. An aqueous solution of 0.1% formic acid served as mobile phase A and an ACN with 0.1% formic acid was used as mobile phase B for the analysis of samples in the positive ion mode. An aqueous solution containing 10 mM ammonium acetate served as mobile phase A and ACN was used as mobile phase B for the analysis of samples in the negative ion mode. The gradient elution program in positive ion mode for serum samples was set as follows: 5% B (0-3 min), 5-68% B (3-10 min), 68-98% B (10-20 min), 98% B (20-23 min). The gradient elution program in negative ion mode for serum samples was set as follows: 5-60% B (0-5 min), 60-98% B (5-15 min), 98% B (15-20 min). The gradient elution program in positive ion mode for tissue samples was set as follows: 5% B (0-3 min), 5-50% B (3-8 min), 50-98% B (8-18 min), 98% B (18-21 min). The gradient elution program in negative ion mode for tissue samples was set as follows: 5% B (0-1 min), 5-75% B (1-6 min), 75-98% B (6-16 min), 98% B (16-21 min). The flow rate was set at 0.3 mL/min and The column temperature was maintained at 40 ◦C. Full MS DDMS^2^ was used for ion scanning with a scan range of 75–1150 Da. The step collision energy was set to 20, 40, and 60 eV.

ACQUITY UPLC BEH C18 (1.7 µm, 2.1 mm×100 mm) was used as a novel mass spectrometry probe for structural confirmation of TPP-BSA, isolation of DL-AAs, and targeted AAs metabolomics. TPP-BSA was analyzed in positive ion mode using 0.1% formic acid aqueous solution as mobile phase A and 0.1% formic acid acetonitrile as mobile phase B. The gradient elution program was set as follows: 10-90% B (0-10 min). Full MS mode was used for ion scanning. Isolation of DL-AAs and targeted AAs metabolomics were analyzed in positive ion mode using 0.1% formic acid aqueous solution as mobile phase A and 0.1% formic acid methanol. The gradient elution program was set as follows: 25% B (0-15 min), 25-40% B (20-35 min), 40-90% B (35-40min). Scanning data was performed using ions with a scale of 100-1000 m/z while in PRM mode and the collision energy was set to 40 eV. Monitoring of four varieties of product ions' derivative was performed using PRM, each variety was Ala-TPP-BSA m/z 531.24→269.14/460.20, Thr-TPP-BSA m/z 561.25→299.16/460.20, His-TPP-BSA m/z 597.26→335.17/460.20, Tyr-TPP-BSA m/z 623.26→361.17/460.20, Val-TPP-BSA m/z 559.27→297.18/460.20, Ile/Leu-TPP-BSA m/z 573.28→311.19/460.20 Gly-TPP-BSA m/z 517.22→255.13/460.20, Asn-TPP-BSA m/z 574.24→312.15/460.20, Glu-TPP-BSA m/z 589.24→327.15/460.20, Phe-TPP-BSA m/z 607.27→345.18/460.20, Asp-TPP-BSA m/z 575.22→313.13/460.20, Met-TPP-BSA m/z 591.24→329.15/460.20, Met-TPP-BSA m/z 646.28→384.19/460.20, IS-TPP-BSA m/z 685.17→423.08/460.20. These products were used for qualitative and quantitative analysis because of labeling and increased signal intensity of AAs characteristic ion fragments.

*2.9 Data acquisition and statistical analysis*

Xcalibur 4.0 software was used for the acquisition of data analyzed by UHPLC-MS/MS. Compound Discoverer 3.2 software was used for the acquisition and analysis of non-targeted metabolism data in terms of retention time, m/z, peak intensity, prerequisite example information, and fragment ion information. The Human Metabolome Database (HMDB), Kyoto Encyclopedia of Genes and Genomes (KEGG), ChemSpider, and mzCloud online databases were used for information matching of the screened compounds. The mass and retention time windows were less than 5 ppm. mzCloud Best Match scores were greater than 70. Metaboanalyst 6.0 software performed principal component analysis (PCA) and orthogonal partial least squares discriminant analysis (OPLS-DA) on the normalized data to screen for significantly different metabolites between the serum of HV and CRC, as well as between the cancerous tissue and the paracancerous tissue of CRC. In addition, Metaboanalyst 6.0 software was used for differential metabolite pathway enrichment analysis. A compound was considered a significant differential metabolite when all metabolites between groups met log_2_FC ≥ 1, log_2_FC ≤ -1, *P* ≤ 0.05, and VIP value ≥ 1. The metabolites were analyzed by Metaboanalyst 6.0 software. Volcano plot, Student t-test, and ROC curve were employed for statistical analysis using the GraphPad Prism v8.02 software for the screening of DL- AAs differentials. The *P* < 0.05 was considered statistically significant. The complete pathway enrichment analysis plot was done on ipath 3.0 software. (https://pathways.embl.de/).

**3. Discussion**

CRC is among the most common malignant tumors affecting the gastrointestinal tract worldwide. [6]. Clinicopathological data indicate that the average age of onset for CRC has decreased from 50 to 40 years, with the incidence rate steadily rising in China in recent years. Due to the limitations in diagnostic methods and technology, CRC is often detected at advanced stages, after it has spread to other organs, which makes treatment considerably harder. The five-year survival rate decreases to less than 10% at this stage. Early diagnosis and surgical treatment can greatly decrease the death rates from CRC [7]. Colonoscopy, barium enema, and fecal occult blood tests (FOBT) are techniques used for CRC screening [8,9]. While colonoscopy remains the gold standard for CRC detection, it is highly invasive and cannot precisely determine the cancer stage. Carcinoembryonic antigen (CEA) and carbohydrate antigen 19-9 (CA19-9) are currently the primary blood biomarkers used to monitor CRC, but their limited sensitivity and specificity in early diagnosis restrict their ability to assess the disease's progression accurately. Hence, discovering biomarkers with improved specificity and sensitivity is important for the early detection and warning of CRC.

In this study, untargeted metabolomics was employed to analyze serum and tissue samples from patients with CRC. Metabolites that differed significantly from controls were screened using the appropriate statistical techniques. The identified metabolites were then mapped to relevant pathways that may contribute to CRC pathogenesis. To optimize the ionization efficiency of low-abundance compounds in mass spectrometry (MS), chromatographic conditions were fine-tuned. As illustrated in Figs. S1A and S2A, chromatographic peaks from serum and tissue samples exhibited good separation across various mobile phases and gradients. Compound Discoverer 3.2 software, in conjunction with the HMDB and KEGG databases, was utilized for compound identification. Volcano plots were generated using GraphPad Prism 8.02 to perform univariate analysis, applying screening parameters of log_2_FC ≥ 1, log_2_FC ≤ -1, and *P* < 0.05. Figs. S1B and S2B display the volcano plots of serum and tissue samples from patients with CRC at different stages, with red indicating upregulated compounds and blue representing downregulated ones. Further analysis of significantly altered metabolites between patients with CRC and healthy volunteers (HVs) was conducted using OPLS-DA, as shown in Figs. 1A, 1B, S1C, and S2C. Except for tissue samples in the negative ion model, all other groups demonstrated clear differentiation between CRC and control groups. Finally, using a VIP ≥ 1 threshold, metabolites that significantly differed between CRC and control samples were identified in serum and tissue samples (Tables S2 and S3). Cluster analysis heatmaps were generated to visually represent the differential metabolite changes between CRC and control groups. The results, presented in Figs. S1D and S2D, illustrate upregulation in red, downregulation in blue, and varying color shades to indicate the strength of the correlation.

A trend toward overexpression was observed in the CRC patient group for the majority of the differential metabolites. Among these, palmitoyl ethanolamide and glycerophospho-N-palmitoyl ethanolamine (GP-NPEA) were identified as potential disruptors of the cannabinoid system, which may contribute to intestinal cancer and inflammation. GP-NPEA serves as the metabolic precursor of palmitoyl ethanolamide [10,11]. Additionally, linoleoyl ethanolamide may suppress LPS-induced increases in cyclooxygenase-2 and prostaglandin E2 levels, while encouraging the release of pro-inflammatory cytokines such as TNF-α and IL-6. In male patients with CRC, variations were noted in testosterone sulfate, the receptor antagonist SSR240612, 15-epi prostaglandin A1, 11β-prostaglandin F2α, thromboxane B2, and prostaglandin F2A. Thromboxane B2, a biomarker for bladder cancer, may be linked to the proximity of the rectum to the bladder and prostate in men, suggesting potential metastasis to these organs [10-14]. Furthermore, 7-methylguanosine (m7G) and N4-acetylcytidine, both involved in RNA synthesis and modification, were found to be significant. m7G has been shown to influence tumorigenesis and progression by regulating the expression of oncogenes, and it serves as an independent biomarker for biochemical recurrence-free survival in prostate cancer [15]. N4-acetylcytidine may act as a cancer inhibitor by reducing hypoxia tolerance *in vivo* [16,17]. Additionally, arachidic acid and N-acetylneuraminic acid, involved in unsaturated fatty acid and sialylated glycan synthesis, respectively, were upregulated in patients with CRC, reflecting their role in regulating inflammatory responses. Current research indicates that salivary acidified polysaccharides in the serum and tissues of CRC patients show a downward trend [18]. Significant changes were also observed in serum and tissue levels of several dipeptides and AAs, with patterns consistent with previous reports [19]. Notably, a comparison of differential metabolites in serum and tissue revealed that most compounds showed a similar trend in the CRC group; however, some compounds, such as Val, exhibited opposite trends in content. Furthermore, serum biomarkers linked to bladder cancer, such as thromboxane B2 and testosterone sulfate, did not show significant differences in tissue samples. This suggests that metabolites identified in tissue samples may more accurately reflect cancer progression compared to those found in serum.

To further explore the potential pathogenesis underlying CRC, pathway enrichment analysis was performed on the significantly different metabolites in serum and tissues, with results presented in Fig. 1C. These metabolites were predominantly enriched in pathways related to unsaturated fatty acid synthesis, arachidonic acid metabolism, and AAs synthesis and metabolism. Notably, the synthesis of unsaturated fatty acids is closely linked to cancer development. Some studies have shown that the connection of ω-6/ω-3 polyunsaturated fatty acids correlate with cancer risk, where ω-3 polyunsaturated fatty acids may exert anticancer effects, while ω-6 polyunsaturated fatty acids may increase the risk of certain cancers. Moreover, elevated fatty acid synthesis in cancer cells alters cellular lipid composition, with fatty acid uptake being essential for cancer cell growth [20]. Arachidonic acid, an ω-6 fatty acid, is a critical component of cell membranes and can be metabolized into prostaglandins (PGs), leukotrienes (LTs), and thromboxanes through enzymes such as cyclooxygenase (COX) and lipoxygenase (LOX). Extensive research has shown a strong association between prostaglandins and CRC development, and this study also detected significant differences in the levels of PGs in the CRC group [21]. AAs, as the fundamental units of protein synthesis, play vital roles in tumor cell growth, metabolism, signaling, immune regulation, and other cellular functions. Among them, Gly and serine (Ser) are interconvertible *via* serine hydroxymethyltransferase, with serine involved in the glycolytic pathway that supplies ATP and energy for cell growth, thus supporting tumor proliferation. The accumulation of Ser accelerates glycolysis, further contributing to cancer initiation and progression. Additionally, Met and Thr are crucial for cellular biosynthesis, with Met participating in the methionine cycle and Thr in the folate cycle, both of which are involved in carbon metabolism [22,23]. Although the exact mechanism by which Val, a branched-chain AAs, contributes to cancer remains unclear, most studies have revealed the downregulation of Val levels in patients with CRC, suggesting that Val synthesis and metabolism may directly impact CRC growth and progression. These metabolic pathways are predominantly represented by amino acid-related synthesis and metabolic processes. Among the top 25 metabolic pathways enriched in differential metabolites, 11 are related to AAs. This prompted further investigation into the relationship between DL- AAs and CRC, with a particular focus on AAs as the central subject of study.

Since 19 of the 20 essential AAs possess chiral centers, isolating and detecting DL- AAs in the human body is crucial for thoroughly exploring the relationship between AAs and CRC. Current chiral separation methods based on high-performance liquid chromatography (HPLC) include direct and indirect approaches [24]. The direct method primarily relies on chiral columns or chiral mobile phases. However, its application is limited due to the high cost of chiral columns and the difficulty in formulating chiral mobile phases. Additionally, the direct method is not well-suited for trace detection of DL- AAs in biological samples because of their strong hydrophilicity and poor ionization efficiency. In contrast, the indirect method is effective in generating diastereoisomers of DL- AAs through pre-column derivatization, enabling analysis on less expensive reversed-phase chromatographic columns. This method also significantly improves the hydrophobicity and ionization efficiency of DL- AAs, enhancing detection sensitivity. Consequently, the indirect approach is widely used for the separation and analysis of various chiral compounds. As shown in Table S4, existing MS derivatization reagents for chiral splitting of DL- AAs include SIPAL [25], FDAA [26], DBD-PyNCS [27], DBD-M-Pro [28], (S)-NIFE [29], (R)-BiAC [30], (S)-COXA-OSu [31], D-BPCl [32], and DATAN [3,33]. Most of these reagents can effectively label and separate certain DL-AAs. For example, Pandey et al. used the DATAN reagent to separate 214 chiral compounds in serum, including 17 essential DL-AAs. The detection limits (LODs) of the currently developed mass spectrometry reagents are mostly between pmol and fmol, which is advantageous for the quantitative analysis of trace amounts of DL-AAs in biological samples. However, complete separation of all 19 DL-AAs under identical chromatographic conditions remains challenging. Additionally, simple and easy-to-operate derivatization reaction conditions are also essential criteria for evaluating chiral mass spectrometry probes. Therefore, the continued development of new MS probes is of critical importance.

In this study, a novel MS probe, TPP-BSA, was synthesized using triphenylphosphine as the parent nucleus. This probe incorporates a permanent positive charge and an α-acetoxyacetamide activation group. It is worth emphasizing that TPP-BSA does not require the additional condensing agents when derivatizing DL-amino acids, and can complete the complete labeling of AAs within 1 hour. It greatly avoids the occurrence of corresponding side reactions, such as the self-condensation of AAs. Besides, the structure of TPP-BSA contains a chiral center, allowing it to form diastereoisomers with DL- AAs and achieve chiral separation in inexpensive reversed-phase chromatography. The structural formula of the probe is depicted in Fig. S3A. The probe's structure was confirmed through LC-MS, ^1^H-NMR, and 13C-NMR, as shown in Fig. S3B and C. In positive ion mode, the retention time of TPP-BSA was 6.24 min at m/z 669.25269, aligning with the theoretical value. The ^1^H-NMR data for TPP-BSA were (300 MHz, CDCl3) *δ* 7.83 –7.57 (m, 19H), 4.40 – 4.32 (m, 1H), 3.57 (s, 1H), 3.53 (d, *J* = 6.6 Hz, 1H), 3.22 (d, *J* = 13.3 Hz, 1H), 3.18 – 3.10 (m, 1H), 2.91 (s, 3H), 2.45 (d, *J* = 3.2 Hz, 2H), 2.43 (s, 3H), 2.39 (s, 1H), 2.00 – 1.83 (m, 5H), 1.64 (s, 2H), 1.47 – 1.18 (m, 2H). The ^13^C-NMR data for TPP-BSA were (75 MHz, CDCl3) *δ* 171.47 (s), 169.90 (s), 146.46 (s), 144.44 (s), 135.35 (s), 135.31 (s), 133.61 (s), 133.48 (s), 130.80 (s), 130.80 (s), 130.80 (s), 130.80 (s), 130.80 (s), 130.80 (s), 130.80 (s), 130.63 (s), 129.72 (s), 128.15 (s), 118.65 (s), 117.51 (s), 101.64 (s), 58.87 (s), 47.16 (s), 36.80 (s), 32.78 (s), 28.89 (s), 25.16 (s), 24.95 (s), 22.55 (s), 21.79 (s), 21.70 (s).

In this study, chiral separation of 19 DL- AAs was conducted using TPP-BSA and an ACQUITY UPLC BEH C18 (2.1×100 mm, 1.7 μm) column. As shown in Fig. 1E, 12 DL- AAs derivatives were successfully separated under the same gradient, with well-shaped peaks for each AAs. The chiral separation parameters for 13 AAs are presented in Table S5, with Rs values of 1.22, 1.81, 2.75, 2.31, 5.30, 4.06, 4.96, 11.37, 6.72, 14.50, 11.57, 11.77, and 7.06 for D/L-His, D/L-Asn, D/L-Glu, D/L-Asp, D/L-Thr, D/L-Ala, D/L-Tyr, D/L-Val, D/L-Met, D/L-Ile, D/L-Leu, D/L-Phe, and D/L-Trp, respectively. The separations for 12 DL- AAs were greater than 1.5, except for D/L-His. Further investigation of the appropriate fragmentation ions for TPP-BSA-labeled AAs derivatives was conducted to support the development of a quantitative method for DL- AAs detection. Stepwise collision energy in Full MS DDMS2 mode was used to detect 13 DL- AAs, Gly, and IS derivatives, with the findings shown in Fig. S4. The mass spectra revealed precursor ion information and characteristic fragment ions for 14 AAs and IS derivatives. Notably, both the IS derivatives and the 14 TPP-BSA-labeled AAs produced fragment ions originating from the TPP-BSA derivatization reagent, with m/z 460.20386. The chemical bond between 5-bromopentanoic acid and triphenylphosphine was cleaved, producing fragment ions with a portion of the derivatization reagent attached to the AAs. For example, the TPP-BSA-Ala derivative produced a fragment ion with m/z 269.14975, confirming the structure of the AAs derivatives.

Linearity was assessed by repeated measurements of DL- AAs spiked at different concentrations in artificial serum. Each DL- AAs showed excellent linearity (R^2^ ≥ 0.99) at the respective concentration. The calibration curves and LODs for each DL- AAs are provided in Table S6, with LODs ranging from 0.2 to 4.5 fmol. This is basically consistent with the currently developed MS derivatization reagents and is suitable for trace analysis in biological samples. Precision and accuracy were evaluated using different concentrations of DL- AAs, with results presented in Table S7. Relative standard deviations (RSDs) were all less than 15%. Matrix effects and recovery rates for each AAs in serum are summarized in Tables S8 and S9, with matrix effects ranging from 4.4% to 14.58% and RSD values for recoveries between 1.88% and 12.78%. Stability analysis under various conditions showed that the AAs derivatives remained stable, with results presented in Table S10. The stability was confirmed by relative error (RE) and RSD values. In summary, the methodologies developed in this study comply with the CFDA methodological guidelines and are suitable for the quantitative analysis of AAs in serum.

Herein, serum levels of DL- AAs were measured in 70 HVs and 68 patients with CRC, with results shown in Tables S11, S12, S13, and S14. The serum levels of DL- AAs in both groups were in the micromolar (μM) range, with L- AAs generally present at significantly higher concentrations than their D-counterparts. Among the AAs, L-Ala had the highest content in human serum, approximately 452.14±134.7 μM in HVs and 292.44±115.86 μM in patients with CRC. Conversely, D-Met had the lowest content, with values around 0.21±0.08 μM in HVs and 0.28±0.08 μM in patients with CRC. To validate the accuracy of the quantification method used in this study, the AAs concentrations were compared with those reported in the literature. The results demonstrated good consistency, supporting the reliability of the AAs levels obtained in this study [34,35]. Although L- AAs are crucial for protein synthesis and maintaining normal physiological functions, certain L- and D- AAs can interconvert during tumor growth, which may influence serum D- AAs levels. Previous studies have suggested a correlation between the levels of some D- AAs and inflammatory diseases such as COVID-19 [36] and cancer [37]. As shown in Figs. 1F and S5A, statistical analysis revealed that compared to HVs, L-His (*P* < 0.0001), Gly (*P* < 0.0001), L-Ala (*P* < 0.0001), L-Tyr (*P* < 0.001), L-Val (*P* < 0.0001), and L-Leu (*P* < 0.001), as well as D-Asn (*P* < 0.0001) and D-Met (*P* < 0.05), exhibited upregulation in patients with CRC, while L-Asn (*P* < 0.0001), D-Asp (*P* < 0.0001), D-Tyr (*P* < 0.05), D-Val (*P* < 0.0001), D-Ile (*P* < 0.01), and D-Leu (*P* < 0.001) were downregulated. These findings were consistent with the results obtained from the untargeted metabolomics analysis. To assess the diagnostic potential of these differential AAs, ROC curve analysis was performed to determine their correlation with CRC diagnosis. As shown in Fig. S5B, the AUC values for these differential AAs ranged from 0.5996 to 0.8029, with D-Asp showing the highest correlation with CRC. However, it is important to note that changes in AAs levels are common across many types of cancer (including CRC), and using AAs alone for diagnosis may not provide sufficient specificity or accuracy. CEA is a commonly used biomarker for CRC diagnosis but has limitations in sensitivity and specificity when used in isolation. Therefore, the differential DL- AAs identified in this study were analyzed in conjunction with clinical CEA levels to explore new potential biomarkers for CRC diagnosis, aiming to improve diagnostic accuracy. As shown in Fig. 1G, the AUC values for L-His, L-Asn, L-Ala, L-Val, D-Asn, and D-Asp were all greater than 0.8 when combined with CEA antigen, indicating that the combination of these AAs with CEA effectively enhanced the accuracy of CRC diagnosis. Notably, the AUC value for Gly when combined with CEA was 0.9104, suggesting that the combination of Gly and CEA offers superior diagnostic potential for CRC.

While some DL- AAs in the serum of patients with CRC exhibited consistent trends, others did not show statistical significance or were only significant in isolated configurations. To further explore these discrepancies, the ratios of D- AAs to L- AAs in CRC serum were analyzed. The results, shown in Fig. S5C, revealed that the ratios of His (*P* < 0.0001), Asn (*P* < 0.05), Ala (*P* < 0.0001), Tyr (*P* < 0.01), Val (*P* < 0.001), Met (*P* < 0.0001), and Trp (*P* < 0.01) were upregulated in the CRC group, while the ratios of Asp (*P* < 0.0001) and Ile (*P* < 0.05) showed downregulation. Analysis of the AUC values further demonstrated that the DL ratio of His was particularly effective in distinguishing CRC. This ratio-based analysis offers a more comprehensive reflection of the metabolic processes involving AAs, providing valuable insights into CRC pathogenesis and potential biomarkers for the disease. Subsequently, multivariate statistical analysis was performed using the D- AAs, L- AAs, and DL- AAs ratios to differentiate between HVs and patients with CRC. While the two groups exhibited a certain trend of separation, complete differentiation was not achieved based solely on these AAs (Fig. S5D). The differential D- AAs and L- AAs were further subjected to pathway enrichment analysis using MetaboAnalyst 6.0 software. The results, shown in Fig. S5E, identified several metabolic pathways related to AAs in CRC, including alanine, aspartate, and glutamate metabolism, histidine metabolism, the glyoxylate cycle, and glutathione metabolism. Among these, alanine, aspartate, and glutamate metabolism, along with the glyoxylate cycle, are critical components of the tricarboxylic acid cycle. These pathways are involved in energy metabolism and cell biosynthesis. In rapidly proliferating cancer cells, this metabolic reprogramming leads to increased protein synthesis and energy production, which, in turn, results in the depletion of certain AAs levels. Aspartic acid is correlated with the histidine metabolic pathway, and histidine levels may influence cellular redox status and energy metabolism. Consequently, the glutathione metabolic pathway, involving sulfhydryl compounds that significantly impact redox reactions, is also linked to oxidative stress within cells. These findings suggest that oxidative stress plays a role in the pathogenesis of CRC. To comprehensively map the relevant pathways involved in CRC development, this study combined non-targeted metabolomics and DL- AAs metabolomics, creating a unified metabolic pathway map using ipath 3.0 software (Fig. S6).

As patients with CRC are clinically staged based on pathological findings, the study further analyzed serum D-AAs and L- AAs levels, along with the D/L- AAs ratio, in patients at different CRC stages to explore the correlation between AAs content and disease progression. As shown in Figs. 1I, S7A and B, most AAs variations did not show significant changes between stage-II and stage-III patients. However, D-Val, L-Val, and D-Leu levels were further reduced in stage-III patients compared to stage-II. L-Trp and D-Trp, which showed no differences in stage-II, exhibited significant changes in stage III, suggesting that these AAs may serve as unique biomarkers for stage-III CRC. Additionally, AAs such as L-Asn and L-Phe displayed decreased variability as the disease progressed, potentially due to the significantly lower number of stage-III patients compared to stage II. This hypothesis will be further validated by increasing the sample size in future studies. The analysis of D/L-AAs ratios between stage-II and stage-III patients revealed that the significance of Val increased with disease progression, providing important insights into the underlying mechanisms of CRC.

**References**

1. A.L. Tan, X.X. Ma, Exploring the functional roles of small-molecule metabolites in disease research: Recent advancements in metabolomics, Chinese Chem. Lett. 35 (2024), 109276.
2. Y. Liu, Z. Wu, D.W. Armstrong, et al., Detection and analysis of chiral molecules as disease biomarkers, Nat. Rev. Chem. 7 (2023) 355−373.
3. R. Pandey, M. Collins, X. Lu, et al., Novel Strategy for Untargeted Chiral Metabolomics using Liquid Chromatography-High Resolution Tandem Mass Spectrometry, Anal. Chem. 93 (2021) 5805−5814.
4. C. Lella, L. Nestor, D. De Bundel, et al., Targeted Chiral Metabolomics of D-Amino Acids: Their Emerging Role as Potential Biomarkers in Neurological Diseases with a Focus on Their Liquid Chromatography-Mass Spectrometry Analysis upon Chiral Derivatization, Int. J. Mol. Sci. 25 (2024), 12410.
5. J. Zhang, H. Zhu, W. Liu, et al., Prognostic and predictive molecular biomarkers in colorectal cancer, Front. Oncol. 15 (2025), 1532924.
6. C. Eng, T. Yoshino, E. Ruíz-García, et al., Colorectal cancer, Lancet 404 (2024) 294−310.
7. E. Barauskaite, A. Raciunas, R. Vaicekauskas, Endoscopic Screening and Surveillance of Gastrointestinal Cancer, Cureus 17 (2025), e79274.
8. M. Zhu, Z. Zhai, Y. Wang, et al., Advancements in the application of artificial intelligence in the field of colorectal cancer, Front. Oncol. 15 (2025), 1499223.
9. B.J. Chia, Y. Ruan, C.J. Brown, et al., Modeling the Economic and Health Impact of Lowering the Recommended Colorectal Cancer Screening Age in Canada Using Fecal Immunochemical Test versus Colonoscopy, Cancer Epidemiol. Biomarkers Prev. 34 (2025) 990–997.
10. L. Su, Y. Zeng, G. Li, et al., Quercetin improves high-fat diet-induced obesity by modulating gut microbiota and metabolites in C57BL/6J mice, Phytother. Res. 36 (2022) 4558–4572.
11. P. Cifelli, G. Ruffolo, M. Ceccanti, et al., Classical and Unexpected Effects of Ultra-Micronized PEA in Neuromuscular Function, Biomolecules 12 (2022), 758.
12. S. Li, H. Yang, W. Li, et al., ADH1C inhibits progression of colorectal cancer through the ADH1C/PHGDH /PSAT1/serine metabolic pathway, Acta Pharmacol. Sin. 43 (2022) 2709–2722.
13. T. Ishida, S. Nishiumi, T. Tanahashi, et al., Linoleoyl ethanolamide reduces lipopolysaccharide-induced inflammation in macrophages and ameliorates 2,4-dinitrofluorobenzene-induced contact dermatitis in mice, Eur. J. Pharmacol. 699 (2013) 6–13.
14. M. Kiely, G.L. Milne, T.Z. Minas, et al., Urinary Thromboxane B2 and Lethal Prostate Cancer in African American Men, J. Natl. Cancer Inst. 114 (2022) 123–129.
15. S. Xin, Y. Deng, J. Mao, et al., Characterization of 7-Methylguanosine Identified Biochemical Recurrence and Tumor Immune Microenvironment in Prostate Cancer, Front. Oncol. 12 (2022), 900203.
16. Q. Yang, X. Lei, J. He, et al., N4-Acetylcytidine Drives Glycolysis Addiction in Gastric Cancer via NAT10/SEPT9/HIF-1α Positive Feedback Loop, Adv. Sci. 10 (2023), e2300898.
17. S. Zhang, Y. Liu, X. Ma, et al., Recent advances in the potential role of RNA N4-acetylcytidine in cancer progression, Cell Commun. Signal. 22 (2024), 49.
18. Y. Li, Z. Piao, S. Wang, et al., Screening of glycan biomarkers for early detection of colorectal cancer based on novel isotope labeling relative quantitative method, J. Pharm. Anal. 2025. https://doi.org/10.1016/j.jpha.2025.101262.
19. A. Gold, F. Choueiry, N. Jin, et al., The application of metabolomics in recent colorectal cancer studies: A state-of-the-art review, Cancers 14 (2022), 725.
20. S. Xu, T. Chen, L. Dong, et al., Fatty acid synthase promotes breast cancer metastasis by mediating changes in fatty acid metabolism, Oncol. Lett. 21 (2021), 27.
21. W. Lu, A. Aihaiti, P. Abudukeranmu, et al., Arachidonic acid metabolism as a novel pathogenic factor in gastrointestinal cancers, Mol. Cell. Biochem. 480 (2025) 1225–1239.
22. I. Amelio, F. Cutruzzolá, A. Antonov, et al., Serine and glycine metabolism in cance, Trends Biochem. Sci. 39 (2014) 191–198.
23. N. Shyh-Chang, J.W. Locasale, C.A. Lyssiotis, et al., Influence of threonine metabolism on S-adenosylmethionine and histone methylation, Science 339 (2013) 222–226.
24. É. Szökő, I. Vincze, T. Tábi, Chiral separations for D-amino acid analysis in biological samples, J. Pharm. Biomed. Anal. 130 (2016) 100–109.
25. X. Liu, Y. Wu, L. Guo, et al., Comprehensive Profiling of Amine-Containing Metabolite Isomers with Chiral Phosphorus Reagents, Anal. Chem. 95 (2023) 16830–16839.
26. Y. Kuang, Z. Li, B. Zhu, et al., Determination of 26 free chiral amino acids in amino acid industrial products by precolumn derivatization/high performance liquid chromatography-tandem mass spectrometry, Journal of Analytical Testing 43 (2024) 703–713.
27. H. Mizuno, Y. Miyazaki, K. Ito, et al., A rapid and sensitive detection of D-Aspartic acid in Crystallin by chiral derivatized liquid chromatography mass spectrometry, J. Chromatogr. A. 1467 (2016) 318–325.
28. S. Wang, X. Zhou, X. Wang, et al., Simultaneous determination of free DL-amino acids in human hair with a novel DBD-M-Pro derivatization by UHPLC-HRMS: An application in diabetes patients, J. Pharm. Biomed. Anal. 251 (2024), 116425.
29. Z. Li, Y. Xing, X. Guo, et al., Development of an UPLC-MS/MS method for simultaneous quantitation of 11 d-amino acids in different regions of rat brain: Application to a study on the associations of d-amino acid concentration changes and Alzheimer's disease. J. Chromatogr. B Analyt. Technol. Biomed. Life Sci. 1058 (2017) 40–46.
30. M. Harada, S. Karakawa, N. Yamada, et al., Biaryl axially chiral derivatizing agent for simultaneous separation and sensitive detection of proteinogenic amino acid enantiomers using liquid chromatography-tandem mass spectrometry, J. Chromatogr. A. 1593 (2019) 91–101.
31. T. Sakamoto, S. Furukawa, T. Nishizawa, et al., Succinimidyl (3-[(benzyloxy)carbonyl]-5-oxo-1,3-oxazolidin-4-yl)acetate on a triazole-bonded phase for the separation of dl-amino-acid enantiomers and the mass-spectrometric determination of chiral amino acids in rat plasma, J. Chromatogr. A. 1585 (2019) 131–137.
32. R. Huang, K. Shen, Q. He, et al., Metabolic Profiling of Urinary Chiral Amino-Containing Biomarkers for Gastric Cancer Using a Sensitive Chiral Chlorine-Labeled Probe by HPLC-MS/MS, J. Proteome Res. 20 (2021) 3952–3962.
33. X. Liu, Y. Zhang, M. Yuan, et al., Determination of naftopidil enantiomers in rat plasma using chiral solid phases and pre-column derivatization high-performance liquid chromatography, J. Chromatogr. B Analyt. Technol. Biomed. Life Sci. 907 (2012) 140–145.
34. A.B. Leichtle, J.-M. Nuoffer, U. Ceglarek, et al., Serum amino acid profiles and their alterations in colorectal cancer, Metabolomics 8 (2012) 643–653.
35. Y. Yang, Z. Wang, X. Li, et al., Profiling the metabolic disorder and detection of colorectal cancer based on targeted amino acids metabolomics, J. Transl. Med. 21 (2023), 824.
36. S. Kimura-Ohba, Y. Takabatake, A. Takahashi, et al., Blood levels of d-amino acids reflect the clinical course of COVID-19, Biochem. Biophys. Rep. 34 (2023), 101452.
37. J.J.A.J. Bastings, H.M. van Eijk, S.W. Olde Damink, et al., d-amino Acids in Health and Disease: A Focus on Cancer, Nutrients 11 (2019), 2205.


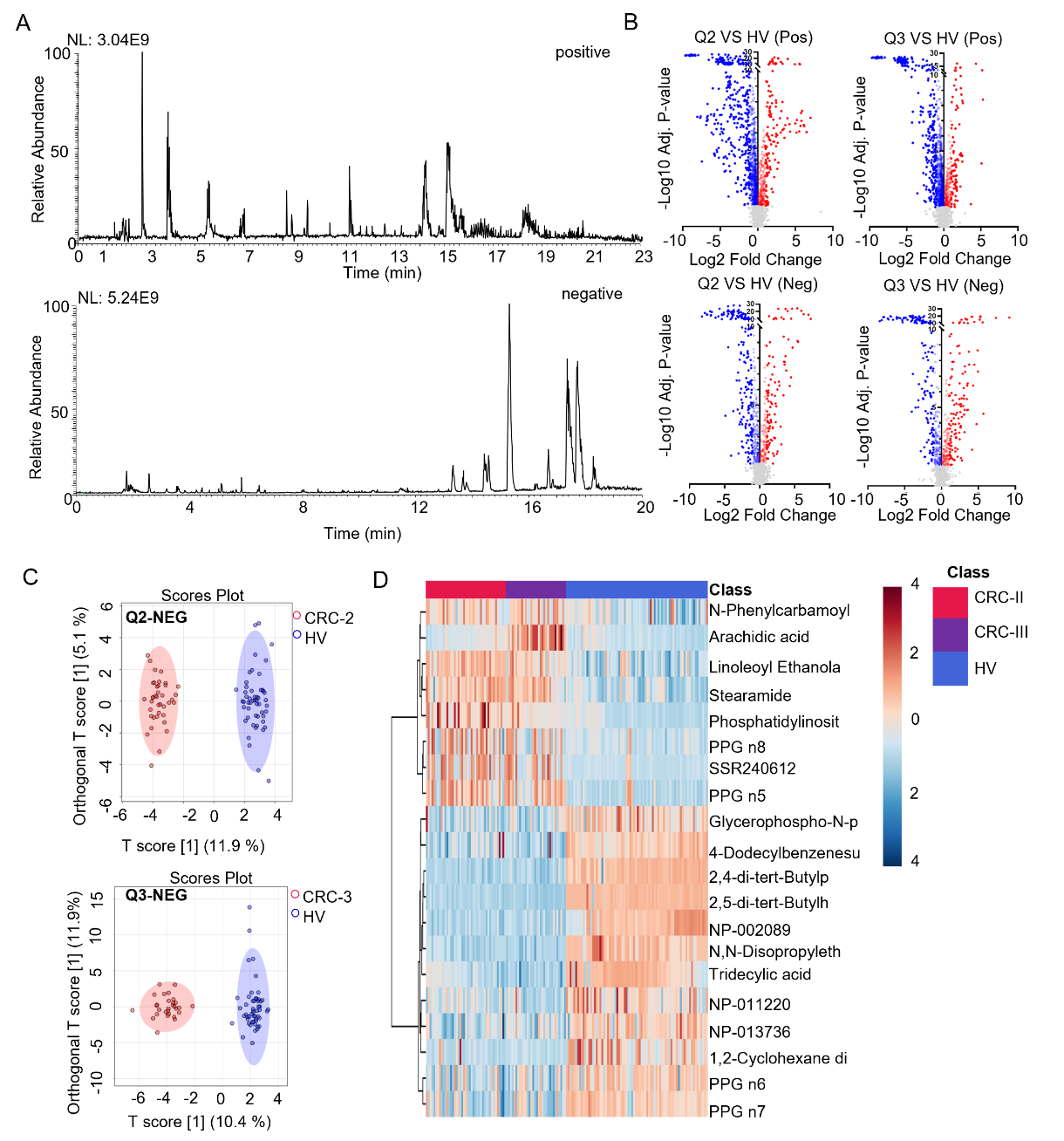


Fig. S1. Serum non-targeted metabolomics results in patients with colon cancer. (A) Base-peak ion chromatogram in positive-negative ion mode. (B) Univariate analysis of volcano plots. (C) Orthogonal Partial Least Squares Discriminant Analysis plots. (D) Differential metabolite cluster analysis plots. (Pos: Positive mode. Neg: Negative mode. HV: Health volunteers. CRC: Colon cancer patients.)
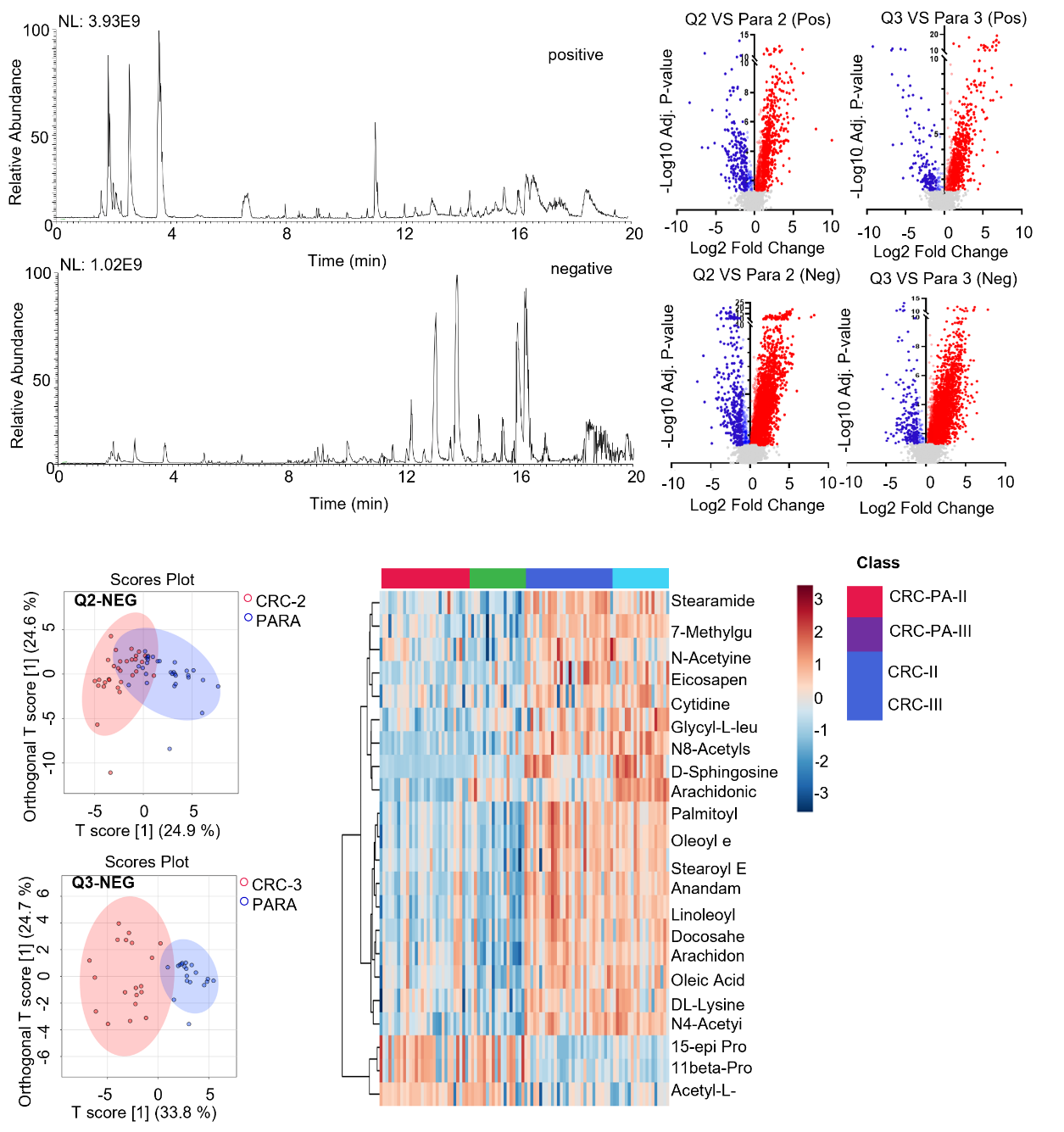


Fig. S2. Tissue non-targeted metabolomics results in patients with colon cancer. (A) Base-peak ion chromatogram in positive-negative ion mode. (B) Univariate analysis of volcano plots. (C) Orthogonal Partial Least Squares Discriminant Analysis plots. (D) Differential metabolite cluster analysis plots. (Pos: Positive mode. Neg: Negative mode. HV: Health volunteers. CRC: colon cancer patients.)


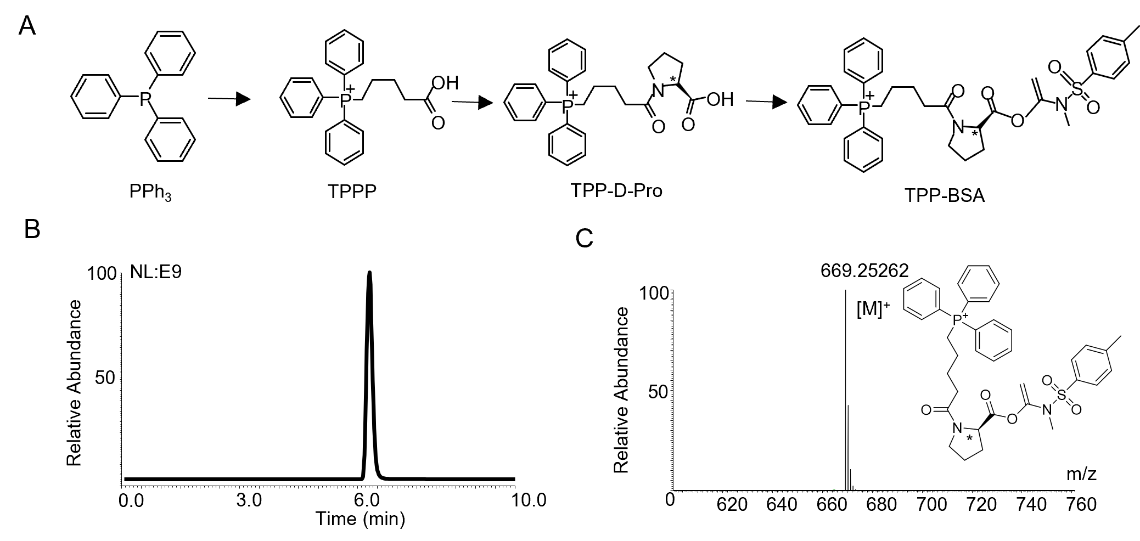


Fig. S3 Synthetic route and structural confirmation of the mass spectrometry probe TPP-BSA. (A) Synthetic route of the mass spectrometry probe TPP-BSA. (B) Extracted ion chromatogram of TPP-BSA. (C) Mass spectra of TPP-BSA. (+ for positive charge; *for asymmetric carbon)


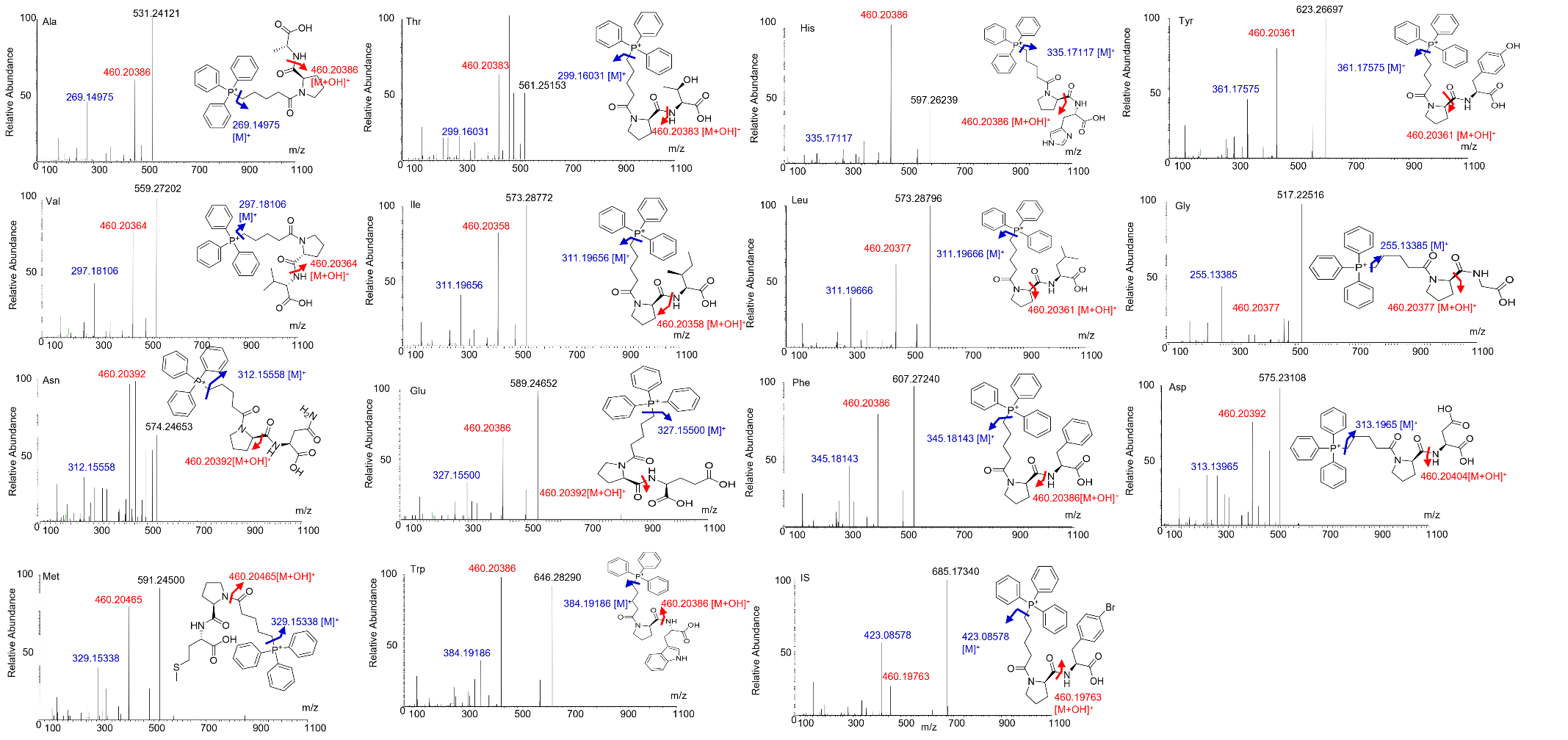


Fig. S4 MS/MS fragments of 14 amino acids and internal standards derivatized by TPP-BSA. (His: Histidine. Asn: Asparaginate. Gly: Glycine. Glu: Glutamic acid. Asp: Aspartic acid. Thr: Threonine. Ala: Alanine. Tyr: Tyrosine. Val: Valine. Met: Methionine. Ile: Isoleucine. Leu: Leucine. Phe: Phenylalanine. Trp: Tryptophan. IS: Internal standard. CEA: Carcinoembryonic antigen.)


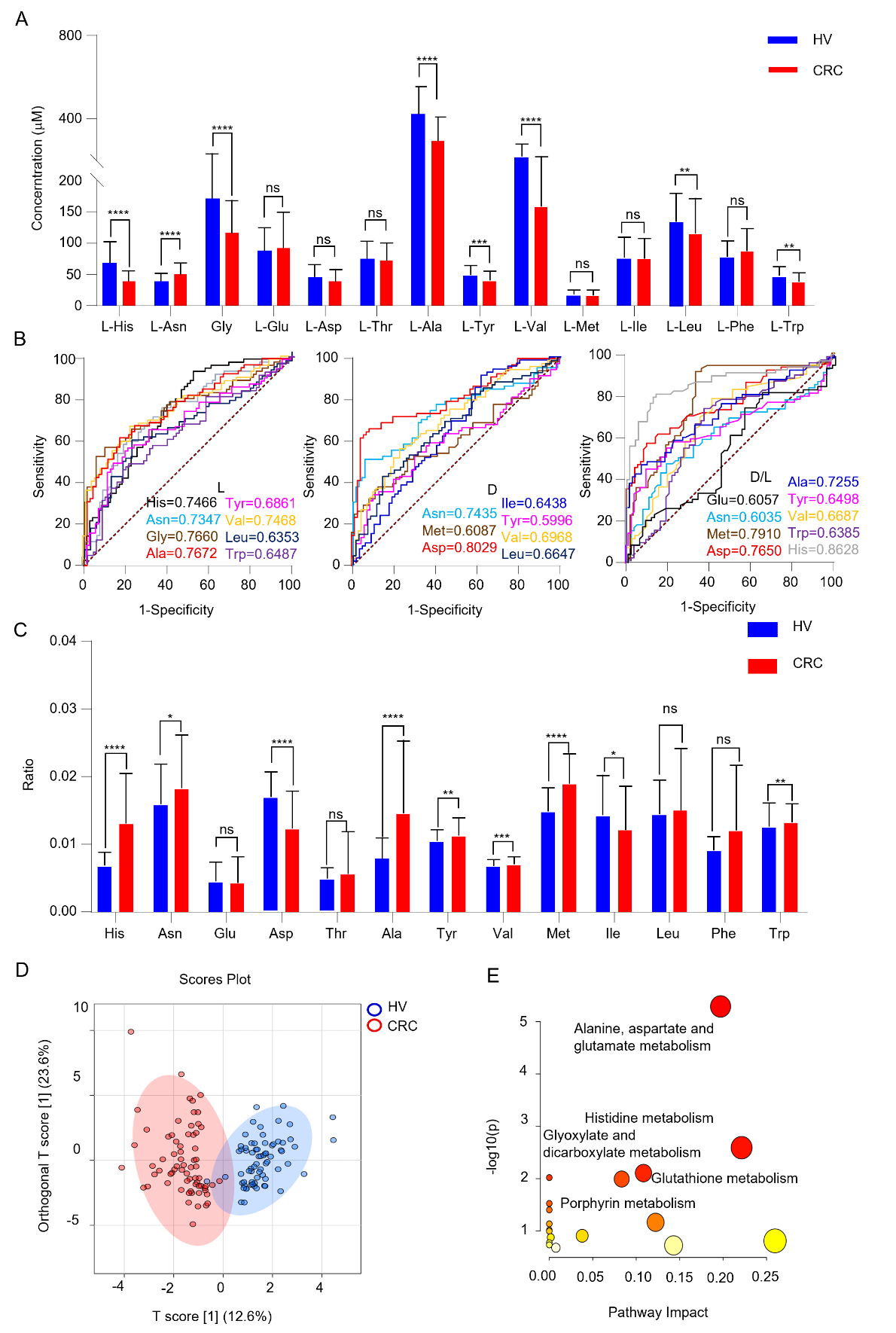


Fig. S5. Differences and statistical analysis of amino acids in serum of healthy volunteers and patients with colorectal cancer. (A) Differences in L-amino acid content. (B) Receiver Operating Characteristic curves of DL-amino acids and D/L-amino acids. (C) D/L-amino acid differences. (D) Orthogonal Partial Least Squares Discriminant Analysis plots. (E) Differential amino acid-related metabolic pathways. (**P* < 0.05, ***P* < 0.01, ****P* < 0.001, *****P* < 0.0001. His: Histidine. Asn: Asparaginate. Gly: Glycine. Glu: Glutamic acid. Asp: Aspartic acid. Thr: Threonine. Ala: Alanine. Tyr: Tyrosine. Val: Valine. Met: Methionine. Ile: Isoleucine. Leu: Leucine. Phe: Phenylalanine. Trp: Tryptophan. IS: Internal standard. CEA: Carcinoembryonic antigen.)


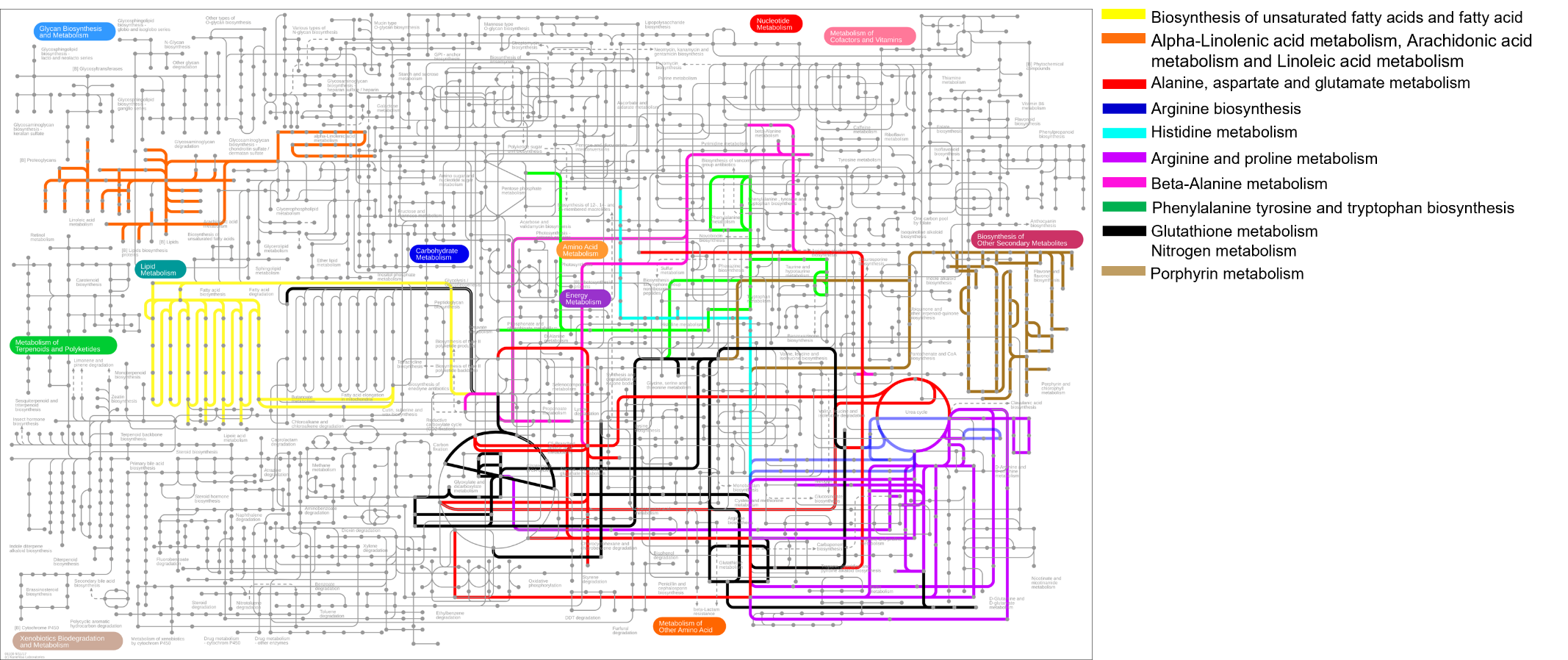


Fig. S6. Ipath analysis developed by ipath 3.0 software (https://pathways.embl.de/). Related pathways that may contribute to CRC.


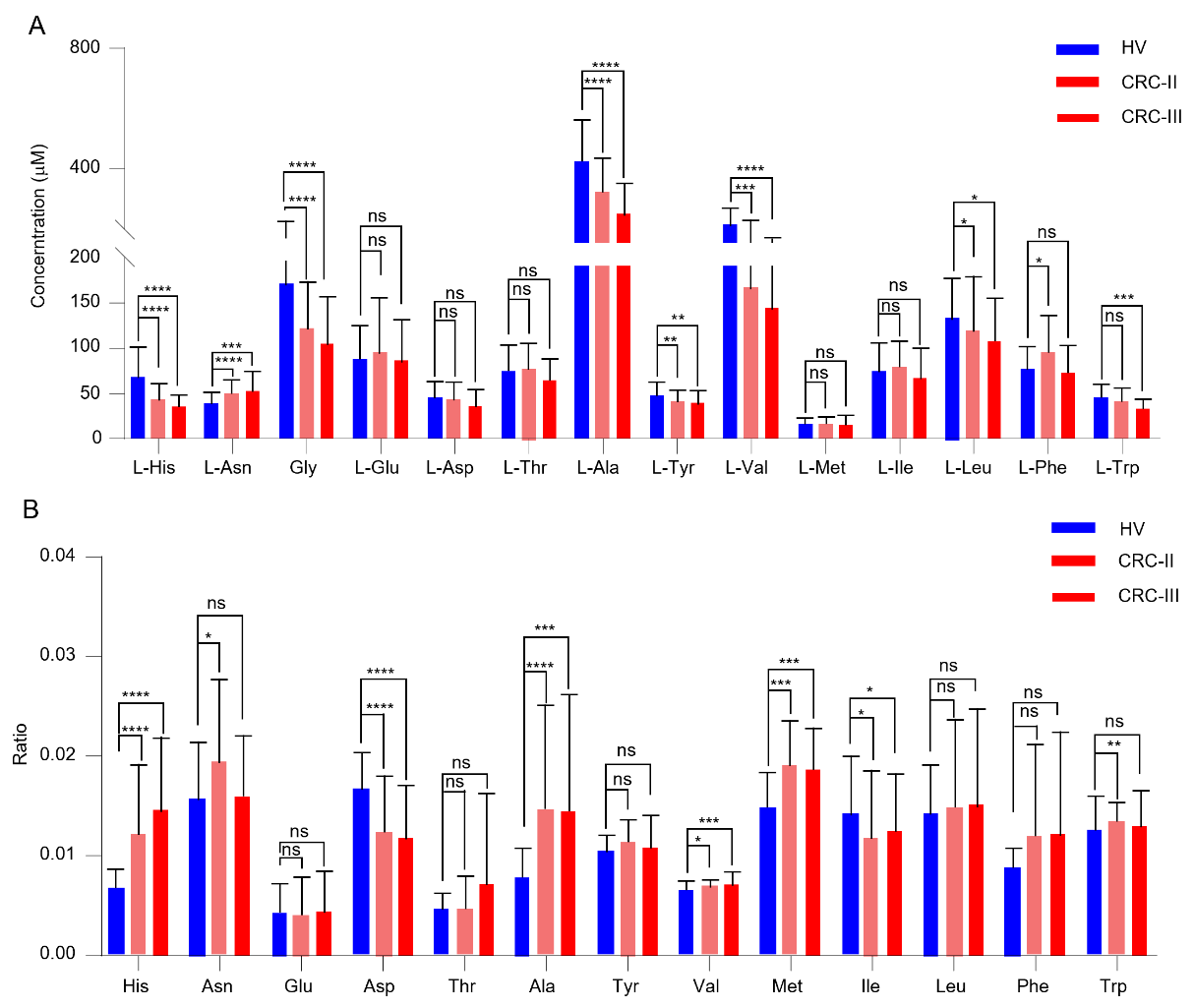


Fig. S7. Differences and statistical analysis of amino acids in serum of healthy volunteers and patients at different stages of CRC. (A) L-amino acids. (B) D/L-amino acids. (**P* < 0.05, ***P* < 0.01, ****P* < 0.001, *****P* < 0.0001. His: Histidine. Asn: Asparaginate. Gly: Glycine. Glu: Glutamic acid. Asp: Aspartic acid. Thr: Threonine. Ala: Alanine. Tyr: Tyrosine. Val: Valine. Met: Methionine. Ile: Isoleucine. Leu: Leucine. Phe: Phenylalanine. Trp: Tryptophan. IS: Internal standard. CEA: Carcinoembryonic antigen.)

Table S1 Information on patients with colorectal cancer

| No | Sex | Age | Stage | CEA | CA19-9 | Tissue |
| --- | --- | --- | --- | --- | --- | --- |
| 1 | Male | 39 | cT3N0M0 IIA | <2.0 | 1.76 | No |
| 2 | Male | 50 | cT3N0M0 IIA | 16.8 | 3.84 | Yes |
| 3 | Male | 58 | cT3N0M0 IIA | 27.7 | 90.2 | Yes |
| 4 | Male | 62 | cT3N0M0 IIA | 13.2 | 9.53 | Yes |
| 5 | Male | 65 | cT3N0M0 IIA | 7.8 | 1.5 | No |
| 6 | Male | 66 | cT3N0M0 IIA | 7.2 | 1.38 | No |
| 7 | Male | 67 | cT3N0M0 IIA | 7.8 | 1.65 | No |
| 8 | Male | 69 | cT3N0M0 IIA | 15.8 | 3.54 | No |
| 9 | Male | 69 | cT3N0M0 IIA | 20.5 | 13.03 | Yes |
| 10 | Male | 71 | cT3N0M0 IIA | 107.2 | 5.49 | Yes |
| 11 | Male | 71 | cT3N0M0 IIA | 11.8 | 125.33 | Yes |
| 12 | Male | 72 | cT3N0M0 IIA | 15.1 | 1.1 | Yes |
| 13 | Male | 73 | cT3N0M0 IIA | 3.38 | 10.82 | No |
| 14 | Male | 73 | cT3N0M0 IIA | 95.3 | 23.3 | No |
| 15 | Male | 73 | cT3N0M0 IIA | 16.3 | 8.09 | No |
| 16 | Male | 74 | cT3N0M0 IIA | 3.99 | 36 | No |
| 17 | Male | 74 | cT3N0M0 IIA | 28.1 | 15.8 | Yes |
| 18 | Male | 74 | cT3N0M0 IIA | 13.2 | 16.9 | Yes |
| 19 | Male | 74 | cT3N0M0 IIA | 41.2 | 4.94 | Yes |
| 20 | Male | 74 | cT3N0M0 ⅡA | 24.8 | 2.13 | No |
| 21 | Male | 75 | cT3N0M0 IIA | 8.89 | 2.8 | Yes |
| 22 | Male | 77 | cT3N0M0 IIA | 2.84 | 5.1 | Yes |
| 23 | Male | 77 | cT3N0M0 IIA | 16.29 | ＜0.8 | Yes |
| 24 | Male | 82 | cT3N0M0 IIA | 7.1 | 5.8 | Yes |
| 25 | Male | 82 | cT4N0M0 ⅡB | 19.46 | 112 | Yes |
| 26 | Male | 84 | cT4N0M0 ⅡB | 3.71 | 3.7 | Yes |
| 27 | Male | 84 | cT3N0M0 ⅡA | 0.86 | 4.3 | Yes |
| 28 | Male | 85 | cT3N0M0 ⅡA | 10.4 | 3.78 | No |
| 29 | Female | 52 | cT3N2aM0 ⅢB | 7.32 | 21.5 | Yes |
| 30 | Female | 53 | cT3N0M0 IIA | 1.13 | 22.6 | Yes |
| 31 | Female | 54 | cT3N0M0 IIA | 11.53 | 14.4 | Yes |
| 32 | Female | 58 | cT3N0M0 IIA | 3.08 | 6.8 | Yes |
| 33 | Female | 63 | cT3N0M0 IIA | 0.69 | 7.1 | Yes |
| 34 | Female | 67 | cT3N0M0 IIA | 7.41 | 63.3 | Yes |
| 35 | Female | 67 | cT3N0M0 IIA | 3.11 | 5 | Yes |
| 36 | Female | 68 | cT3N0M0 IIA | 11.3 | 8.8 | No |
| 37 | Female | 68 | cT3N0M0 IIA | 16.32 | 233.3 | Yes |
| 38 | Female | 69 | cT3N0M0 IIA | 1.29 | 10 | No |
| 39 | Female | 71 | cT3N0M0 IIA | 1.95 | 13 | Yes |
| 40 | Female | 73 | cT3N0M0 IIA | 14.71 | 9.5 | Yes |
| 41 | Female | 74 | cT3N0M0 IIA | 12.89 | ＜0.8 | Yes |
| 42 | Female | 79 | cT3N0M0 IIA | 2.99 | 61 | Yes |
| 43 | Female | 84 | cT3N0M0 IIA | 1.68 | 25.1 | No |
| 44 | Male | 50 | cT3N1M0 ⅢB | 26.98 | 122.9 | Yes |
| 45 | Male | 58 | cT3N2aM0 ⅢB | 17.3 | 2.8 | Yes |
| 46 | Male | 61 | cT3N1M0 ⅢB | 142.83 | 0.8 | Yes |
| 47 | Male | 62 | cT3N2aM0 IIIB | 2.33 | 4.3 | No |
| 48 | Male | 63 | cT3N2bM0 ⅢC | 6.07 | 13.1 | Yes |
| 49 | Male | 66 | cT3N2aM0 ⅢB | 3.47 | 16.1 | Yes |
| 50 | Male | 69 | cT3N2aM0 ⅢB | 1.34 | 8 | No |
| 51 | Male | 69 | cT4N1M0 IIIB | 2.01 | 11.3 | No |
| 52 | Male | 70 | cT3N1bM0 IIIB | 25 | 30.8 | No |
| 53 | Male | 70 | cT3N1aM0 ⅢB | 4.27 | 11.8 | No |
| 54 | Male | 71 | cT4N1M0 IIIB | 48.4 | 64.3 | Yes |
| 55 | Male | 72 | cT3N1bM0 IIIB | 1.87 | 11.2 | No |
| 56 | Male | 72 | cT3N1aM0 ⅢB | 25.2 | 13.2 | Yes |
| 57 | Male | 73 | cT3N1M0 ⅢB | 54.95 | 264.4 | Yes |
| 58 | Male | 73 | cT1N2bM0 ⅢB | 3.09 | 30.4 | Yes |
| 59 | Male | 74 | cT3N1aM0 ⅢB | 7.71 | ＜2 | Yes |
| 60 | Male | 74 | cT3N1aM0 ⅢB | 5.22 | 13.2 | Yes |
| 61 | Male | 74 | cT3N1M0 ⅢB | 0.98 | 2.1 | Yes |
| 62 | Male | 76 | cT3N1bM0 ⅢB | 242.29 | >2001 | Yes |
| 63 | Female | 37 | cT3N1aM0 ⅢB | 0.66 | 6.5 | No |
| 64 | Female | 48 | cT3N1M0 ⅢB | 96.92 | 82.1 | Yes |
| 65 | Female | 59 | cT1N2bM0 ⅢB | 6.36 | 2.6 | Yes |
| 66 | Female | 61 | cT3N1bM0 ⅢB | 1.41 | 6.8 | No |
| 67 | Female | 66 | cT3N1M0 ⅢB | 15.31 | 6.4 | Yes |
| 68 | Female | 68 | cT3N1bM0 ⅢB | 0.51 | 10.4 | Yes |
| 69 | Female | 69 | cT3N1aM0 ⅢB | 2.08 | 13.7 | No |
| 70 | Female | 73 | cT3N2bM0 IIIC | 453 | 171 | No |
| 71 | Female | 76 | cT3N1bM0 ⅢB | 23.91 | 71.2 | Yes |
| 72 | Female | 78 | cT3N1bM0 ⅢB | 1.27 | 73.8 | No |
| 73 | Female | 82 | cT3N1bM0 ⅢB | 85.3 | 68.3 | No |

Table S2 Significant differences in serum metabolites in patients at different stages

| No | Name | Formula | ppm | MW | RT [min] | Ion Mode | CRC/ HV | *P* | Log_2_FC | VIP | Stages |
| --- | --- | --- | --- | --- | --- | --- | --- | --- | --- | --- | --- |
| 1 | DL-Stachydrine | C_7_H_13_NO_2_ | 0.19 | 143.0947 | 2.09 | + | Down | 9.45E-04 | -1.62 | 1.12 | Q3 |
| 2 | Thromboxane B2 | C_20_H_34_O_6_ | -0.19 | 370.2355 | 6.16 | - | Up | 2.46E-06 | 2.17 | 1.2 | Q2/Q3 |
| 3 | Icosapent methyl | C_21_H_32_O_2_ | -0.18 | 316.2402 | 21.95 | + | Down | 2.52E-04 | -1.22 | 1.23 | Q2 |
| 4 | Hippuric acid | C_9_H_9_NO_3_ | -0.14 | 179.0582 | 8.61 | + | Down | 2.22E-04 | -1.46 | 1.24 | Q2 |
| 5 | Cyclo (L-prolyl-L-valyl) | C_10_H_16_N_2_O_2_ | 0.26 | 196.1212 | 8.21 | + | Down | 2.70E-03 | -1.11 | 1.48 | Q2/Q3 |
| 6 | Testosterone sulfate | C_19_H_28_O_5_S | -0.61 | 368.1655 | 6.7 | - | Up | 2.38E-02 | 2.76 | 1.51 | Q3 |
| 7 | Arachidic acid | C_20_H_40_O_2_ | -0.13 | 312.3028 | 21.06 | - | Up | 2.30E-08 | 3.14 | 1.53 | Q3 |
| 8 | L, L-Cyclo (leucylprolyl) | C_11_H_18_N_2_O_2_ | 0.32 | 210.1369 | 9.23 | + | Down | 2.99E-08 | -1.4 | 1.54 | Q2/Q3 |
| 9 | Phosphatidylinositol-1,2-dipalmitoyl | C_41_H_79_O_13_P | -0.03 | 810.5258 | 18.96 | - | Up | 1.63E-09 | 1 | 1.54 | Q2 |
| 10 | 2,2'- Methylenebis (4-methyl-6-tert-butylphenol | C_23_H_32_O_2_ | -0.68 | 340.24 | 16.53 | - | Up | 5.54E-06 | 2 | 1.56 | Q3 |
| 11 | 2,4,6,8,10,12-Docosahexaenoic acid, ethyl ester | C_24_H_36_O_2_ | -0.51 | 356.2714 | 13.09 | + | Down | 2.25E-02 | -2.42 | 1.68 | Q3 |
| 12 | Diisononyl hexahydrophthalate | C_26_H_48_O_4_ | -0.11 | 424.3552 | 25.14 | + | Down | 5.21E-06 | -1.57 | 1.71 | Q2/Q3 |
| 13 | Glycerophospho-N-palmitoyl ethanolamine | C_21_H_44_NO_7_P | -0.02 | 453.2855 | 8.55 | - | Up | 9.44E-05 | 1.62 | 1.8 | Q2/Q3 |
| 14 | Valine | C_5_H_11_NO_2_ | 1.79 | 117.0792 | 2.21 | + | Down | 2.60E-03 | -1.49 | 1.82 | Q2/Q3 |
| 15 | Tridecylic acid | C_13_H_26_O_2_ | -2.81 | 214.1927 | 12.06 | - | Down | 1.08E-05 | -1.55 | 1.92 | Q2/Q3 |
| 16 | Nardosinone | C_15_H_22_O_3_ | -1.23 | 250.1566 | 7.77 | - | Down | 8.60E-23 | -3.7 | 2.13 | Q2/Q3 |
| 17 | Hexapropylene glycol | C_18_H_38_O_7_ | -0.55 | 366.2616 | 10.65 | + | Down | 2.35E-07 | -2.69 | 2.2 | Q2/Q3 |
| 18 | PPG n7 | C_21_H_44_O_8_ | -0.41 | 424.3034 | 11.32 | + | Down | 2.04E-08 | -2.6 | 2.38 | Q2/Q3 |
| 19 | SSR240612 | C_42_H_52_N_4_O_7_S | -2.05 | 756.3541 | 9.03 | + | Up | 8.55E-05 | 1.64 | 2.39 | Q3 |
| 20 | Diisopropylethylamine | C_8_H_19_N | 0.72 | 129.1518 | 25.37 | + | Down | 2.57E-03 | -3.83 | 2.48 | Q2/Q3 |
| 21 | 2,5-di-tert-Butylhydroquinone | C_14_H_22_O_2_ | -2.51 | 222.1614 | 12.1 | - | Down | 2.34E-06 | -5.15 | 2.76 | Q2/Q3 |
| 22 | Octapropylene glycol | C_24_H_50_O_9_ | -0.15 | 482.3454 | 11.91 | + | Down | 5.90E-10 | -2.72 | 2.93 | Q2/Q3 |
| 23 | PPG n5 | C_15_H_32_O_6_ | -0.34 | 308.2198 | 10.01 | + | Down | 2.62E-11 | 2.37 | 3.1 | Q2/Q3 |
| 24 | 2,4-di-tert-Butylphenol | C_14_H_22_O | -3.16 | 206.1664 | 13.48 | - | Down | 7.60E-05 | -1.99 | 3.3 | Q2/Q3 |
| 25 | Stearamide | C_18_H_37_NO | -0.23 | 283.2875 | 25.22 | + | Up | 2.55E-08 | 1.57 | 3.39 | Q2/Q3 |
| 26 | Linoleoyl Ethanolamide | C_20_H_37_NO_2_ | -0.34 | 323.2823 | 17.66 | + | Up | 6.64E-15 | 1.65 | 3.48 | Q2/Q3 |

Table S3 Significantly different metabolites in the tissues of patients at different stages

| No. | Name | Formula | ppm | MW | RT [min] | Ion Mode | CA/ | *P* | log_2_FC | VIP | Stages |
| --- | --- | --- | --- | --- | --- | --- | --- | --- | --- | --- | --- |
|  |  |  |  |  |  |  | PA |  |  |  |  |
| 1 | 8Z,11Z,14Z-Eicosatrienoic acid | C_20_H_34_O_2_ | -1.56 | 306.2554 | 14.61 | - | Up | 3.02E-03 | 1.38 | 1.01 | Q2 |
| 2 | Myristic acid | C_14_H_28_O_2_ | -3.22 | 228.2082 | 12.69 | - | Up | 4.02E-04 | 2.39 | 1.02 | Q3 |
| 3 | 6-keto Prostaglandin F1A | C_20_H_34_O_6_ | -1.9 | 370.2348 | 6.06 | - | Down | 2.38E-02 | -2.05 | 1.04 | Q3 |
| 4 | Palmitoleic acid | C_16_H_30_O_2_ | -2.38 | 254.224 | 13.16 | - | Up | 8.16E-03 | 1.08 | 1.08 | Q3 |
| 5 | Azelaic acid | C_9_H_16_O_4_ | -4.96 | 188.1039 | 2.57 | - | Up | 9.52E-04 | -3.20 | 1.09 | Q3 |
| 6 | Linoleic acid | C_18_H_32_O_2_ | -1.73 | 280.2397 | 13.83 | - | Up | 2.03E-03 | 2.26 | 1.09 | Q2 |
| 7 | 11(Z)-Eicosenoic acid | C_20_H_38_O_2_ | -1.56 | 310.2867 | 19.79 | - | Up | 2.03E-03 | 2.26 | 1.1 | Q2/Q3 |
| 8 | 15-deoxy-xi12,14-Prostaglandin J2 | C_20_H_28_O_3_ | -1.7 | 316.2033 | 8.73 | - | Down | 3.59E-03 | 1.60 | 1.11 | Q3 |
| 9 | α-Linolenic acid | C_18_H_30_O_2_ | -2.02 | 278.224 | 12.09 | - | Up | 7.52E-04 | 1.30 | 1.11 | Q3 |
| 10 | Oleic Acid | C_18_H_34_O_2_ | -1.67 | 282.2554 | 16.25 | - | Up | 4.43E-03 | 1.27 | 1.12 | Q2 |
| 11 | N-Arachidonoyl taurine | C_22_H_37_NO_4_S | -2.16 | 411.2434 | 8.35 | - | Up | 1.61E-03 | 2.40 | 1.12 | Q3 |
| 12 | Indole-3-lactic acid | C_11_H_11_NO_3_ | 0.01 | 205.0739 | 9.52 | + | UP | 1.21E-03 | 1.22 | 1.13 | Q2 |
| 13 | AKK | C_15_H_31_N_5_O_4_ | -0.03 | 345.2376 | 1.69 | + | Up | 2.19E-04 | 1.20 | 1.14 | Q3 |
| 14 | Palmitic Acid | C_16_H_32_O_2_ | -2.23 | 256.2397 | 15.96 | - | Up | 1.34E-04 | 1.84 | 1.18 | Q3 |
| 15 | α-Aspartylphenylalanine | C_13_H_16_N_2_O_5_ | -0.43 | 280.1058 | 7.09 | + | UP | 1.16E-03 | 1.43 | 1.18 | Q2 |
| 16 | Arginine | C_6_H_14_N_4_O_2_ | -0.31 | 174.1116 | 1.81 | + | Up | 3.08E-02 | 1.29 | 1.19 | Q3 |
| 17 | Stearic acid | C_18_H_36_O_2_ | -1.54 | 284.2711 | 19.89 | - | Up | 2.12E-02 | 2.03 | 1.21 | Q2 |

| 18 | Glycyl-L-leucine | C_8_H_16_N_2_O_3_ | -0.74 | 188.116 | 4.03 | + | UP | 2.46E-04 | 1.48 | 1.25 | Q2/Q3 |
| --- | --- | --- | --- | --- | --- | --- | --- | --- | --- | --- | --- |
| 19 | Aspartic acid | C_4_H_7_NO_4_ | 0.16 | 133.0375 | 2.03 | + | Down | 6.63E-04 | -1.16 | 1.25 | Q3 |
| 20 | Icosapent methyl | C_21_H_32_O_2_ | -1.25 | 316.2398 | 18.83 | + | UP | 1.73E-04 | 1.69 | 1.26 | Q2/Q3 |
| 21 | S-Adenosylhomocysteine | C_14_H_20_N_6_O_5_S | -0.75 | 384.1213 | 2.73 | + | Up | 1.13E-02 | 1.98 | 1.26 | Q3 |
| 22 | Arachidonoyl amide | C_20_H_33_NO | -0.82 | 303.256 | 19.07 | + | UP | 2.37E-04 | 1.54 | 1.28 | Q2 |
| 23 | Sphingosine (d18:1) | C_18_H_37_NO_2_ | -0.6 | 299.2823 | 14.79 | + | Up | 5.28E-09 | 8.64 | 1.28 | Q3 |
| 24 | 15-keto Prostaglandin F2A | C_20_H_32_O_5_ | -0.71 | 352.2247 | 12.56 | + | Down | 2.98E-03 | -1.32 | 1.29 | Q2 |
| 25 | D-Sphingosine | C_18_H_37_NO_2_ | -0.62 | 299.2822 | 13.63 | + | Up | 1.52E-04 | 6.84 | 1.3 | Q2/Q3 |
| 26 | N-Acetylneuraminic acid | C_11_H_19_NO_9_ | 0.69 | 309.1062 | 2.05 | + | UP | 1.71E-09 | 3.54 | 1.3 | Q2/Q3 |
| 27 | N1-Acetylspermine | C_12_H_28_N_4_O | -0.45 | 244.2262 | 1.74 | + | UP | 1.18E-06 | 2.29 | 1.31 | Q2 |
| 28 | Adenosine | C_10_H_13_N_5_O_4_ | -0.84 | 267.0965 | 2.61 | + | Down | 4.47E-04 | -1.56 | 1.34 | Q2 |
| 29 | Υ-L-Glutamyl-L-glutamic acid | C_10_H_16_N_2_O_7_ | -0.72 | 276.0956 | 2.14 | + | Down | 1.87E-03 | -1.20 | 1.38 | Q3 |
| 30 | Arachidonic acid | C_20_H_32_O_2_ | -0.88 | 304.24 | 20.11 | + | UP | 3.38E-04 | 1.29 | 1.39 | Q2/Q3 |
| 31 | D-(+)-Pipecolinic acid | C_6_H_11_NO_2_ | 0.02 | 129.079 | 1.78 | + | Up | 1.02E-02 | 1.06 | 1.4 | Q3 |
| 32 | Prostaglandin F2A | C_20_H_34_O_5_ | -1.63 | 354.2401 | 6.54 | - | Down | 3.00E-03 | -2.18 | 1.45 | Q2/Q3 |
| 33 | 3'-AMP | C_10_H_14_N_5_O_7_P | -0.68 | 347.0629 | 2.89 | + | Up | 2.89E-03 | 1.67 | 1.46 | Q3 |
| 34 | Cytidine 5'-monophosphate (hydrate) | C_9_H_14_N_3_O_8_P | -1.16 | 323.0515 | 2.31 | + | UP | 5.51E-05 | 1.51 | 1.5 | Q2/Q3 |
| 35 | Cyclo(leucylprolyl) | C_11_H_18_N_2_O_2_ | -0.68 | 210.1367 | 9.11 | + | UP | 5.32E-05 | 1.09 | 1.51 | Q2/Q3 |
| 36 | DL-Lysine | C_6_H_14_N_2_O_2_ | 0.25 | 146.1056 | 1.75 | + | UP | 2.19E-04 | 1.19 | 1.53 | Q2/Q3 |
| 37 | 11b-PGF2a | C_20_H_34_O_5_ | -0.61 | 354.2404 | 11.99 | + | Down | 1.00E-03 | -2.82 | 1.54 | Q2/Q3 |
| 38 | N8-Acetylspermidine | C_9_H_21_N_3_O | -0.54 | 187.1684 | 1.63 | + | UP | 4.25E-12 | 3.24 | 1.55 | Q2/Q3 |
| 39 | Prostaglandin D2 | C_20_H_30_O_4_ | -0.67 | 334.2142 | 10.25 | + | Down | 1.50E-03 | -2.99 | 1.56 | Q2 |
| 40 | DKK | C_16_H_31_N_5_O_6_ | 0.13 | 389.2275 | 1.71 | + | Up | 4.37E-05 | 1.41 | 1.56 | Q3 |
| 41 | Docosahexaenoyl Ethanolamide | C_24_H_37_NO_2_ | -0.65 | 371.2822 | 17.97 | + | Up | 4.55E-06 | 2.82 | 1.58 | Q3 |
| 42 | Isoleucine | C_6_H_13_NO_2_ | 0.28 | 131.0947 | 2.35 | + | Up | 2.47E-04 | 1.51 | 1.58 | Q3 |
| 43 | 15-epi Prostaglandin A1 | C_20_H_32_O_4_ | -0.74 | 336.2298 | 12 | + | Down | 3.03E-05 | -3.19 | 1.61 | Q2/Q3 |
| 44 | Phenylalanine | C_9_H_11_NO_2_ | -0.4 | 165.0789 | 3.94 | + | Up | 5.24E-04 | 1.16 | 1.66 | Q3 |
| 45 | Anandamide (AEA) | C_22_H_37_NO_2_ | -0.86 | 347.2821 | 18.12 | + | UP | 1.80E-05 | 2.90 | 1.68 | Q2/Q3 |
| 46 | Docosahexaenoic acid | C_22_H_32_O_2_ | -1.07 | 328.2399 | 19.69 | + | UP | 2.67E-06 | 2.52 | 1.69 | Q2/Q3 |
| 47 | Proline | C_5_H_9_NO_2_ | 1.52 | 115.0635 | 2.23 | + | Up | 1.16E-04 | 1.08 | 1.7 | Q3 |
| 48 | L-Tyrosine methyl ester | C_10_H_13_NO_3_ | -0.61 | 195.0894 | 3.58 | + | UP | 2.06E-09 | 4.34 | 1.71 | Q2 |
| 49 | N6-methyladenosine | C_11_H_15_N_5_O_4_ | -0.51 | 281.1123 | 2.58 | + | Up | 2.94E-04 | 1.83 | 1.71 | Q3 |
| 50 | Stearamide | C_18_H_37_NO | -0.96 | 283.2872 | 22 | + | UP | 5.52E-06 | 1.90 | 1.72 | Q2/Q3 |
| 51 | Stearoyl Ethanolamide | C_20_H_41_NO_2_ | -0.83 | 327.3135 | 22.49 | + | UP | 2.84E-04 | 1.55 | 1.77 | Q2/Q3 |
| 52 | Palmitoyl ethanolamide | C_18_H_37_NO_2_ | -0.86 | 299.2822 | 19.68 | + | UP | 1.87E-06 | 2.32 | 1.78 | Q2/Q3 |
| 53 | N4-Acetylcytidine | C_11_H_15_N_3_O_6_ | -0.55 | 285.0959 | 4.25 | + | UP | 1.56E-06 | 2.00 | 1.81 | Q2/Q3 |
| 54 | Linoleoyl Ethanolamide | C_20_H_37_NO_2_ | -0.93 | 323.2821 | 18.32 | + | UP | 5.05E-08 | 2.61 | 1.82 | Q2/Q3 |
| 55 | Oleoyl ethanolamide | C_20_H_39_NO_2_ | -0.95 | 325.2978 | 19.96 | + | UP | 1.65E-07 | 2.54 | 1.83 | Q2/Q3 |
| 56 | Arachidonic acid methyl ester | C_21_H_34_O_2_ | -0.36 | 318.2558 | 22.34 | + | Up | 2.27E-07 | 3.44 | 1.84 | Q2/Q3 |
| 57 | Acetyl-L-carnitine | C_9_H_17_NO_4_ | -0.26 | 203.1157 | 2.14 | + | Down | 2.53E-02 | -1.18 | 1.94 | Q2/Q3 |
| 58 | (±)11(12)-EET | C_20_H_32_O_3_ | -0.46 | 320.235 | 11.10 | + | Down | 1.34E-04 | -2.39 | 2.02 | Q2/Q3 |
| 59 | Kynurenine | C_10_H_12_N_2_O_3_ | -0.18 | 208.0848 | 3.68 | + | UP | 1.70E-10 | 2.08 | 2.02 | Q2 |
| 60 | (±)17(18)-EpETE | C_20_H_30_O_3_ | -0.33 | 318.2194 | 11.10 | + | Down | 1.01E-04 | -3.34 | 2.03 | Q2 |
| 61 | 7-Methylguanosine | C_11_H_15_N_5_O_5_ | -0.62 | 297.1071 | 3.210 | + | UP | 1.47E-05 | 1.64 | 2.04 | Q2/Q3 |
| 62 | Tridemorph | C_19_H_39_NO | -0.16 | 297.3031 | 22.45 | + | UP | 3.40E-08 | 2.58 | 2.04 | Q2 |
| 63 | N,N-Dimethylarginine | C_8_H_18_N_4_O_2_ | -0.26 | 202.1429 | 1.88 | + | UP | 3.06E-06 | 1.41 | 2.09 | Q2/Q3 |
| 64 | Valine | C_5_H_11_NO_2_ | 1.47 | 117.0792 | 2.07 | + | UP | 7.07E-03 | 1.19 | 2.11 | Q2 |

Table S4 Derivatization reagents that can be used to separate chiral amino acids

| Derivatization reagents | Analyte | Rs | LOD | Derivative conditions | Rf |
| --- | --- | --- | --- | --- | --- |
| SIPAL | DL-amino compounds (Cell) | ＞ 1.5 | -- | Rt, 60 min | [25] |
| FDAA | D/L-Amino acids (chemicals) | 0.7-14.9 | 0.03-4.0 nM | 37^o^C, 16 h | [26] |
| DBD-PyNCS | D/L-Asp (protein) | -- | 0.16-0.75 pmol | 70^o^C, 4 h | [27] |
| DBD-M-Pro | D/L-Amino acids (hair) | 1.59-24.11 | 2.5-500 pmol | 60^o^C, 1 h | [28] |
| (S)-NIFE | D-Amino acids (tissue) | 2.45-11.17 | 0.34-51.50 fmol | Rt, 20 min | [29] |
| (R)-BiAC | D/L-Amino acids (food) | 1.91-12.8 | 7.0-127.0 amol | 55^o^C, 10 min | [30] |
| (S)-COXA-OSu | D/L-Amino acids (plasma) | 1.102-4.460 | 1.38-518 fmol | 40^o^C, 90 min | [31] |
| D-BPCl | DL-amino compounds (urine) | 0.87-5.16 | 0.05-17.48 fmol | 4^o^C, overnight | [32] |
| (±)-DATAN | Chiral amino- and hydroxyl- compounds (plasma) | -- | 0.36-22.13 fmol | 75^o^C, 2 h | [3][33] |
| TPP-BSA | D-Amino acids (serum) | 1.22-14.50 | 0.2-4.5 fmol | 70^o^C, 1h | in this study |

| No. | Ammino | *k* | | Rs | α |
| --- | --- | --- | --- | --- | --- |
|  | compounds | L-Isomer | D-Isomer |  |  |
| 1 | His | 9.71 | 8.43 | 1.22 | 1.10 |
| 2 | Asn | 17.06 | 16.08 | 1.81 | 1.06 |
| 3 | Glu | 24.92 | 23.40 | 2.75 | 1.05 |
| 4 | Asp | 24.06 | 22.38 | 2.31 | 1.08 |
| 5 | Thr | 28.65 | 24.02 | 5.30 | 1.15 |
| 6 | Ala | 29.60 | 24.40 | 4.06 | 1.16 |
| 7 | Tyr | 34.20 | 32.94 | 4.96 | 1.04 |
| 8 | Val | 37.62 | 33.23 | 11.37 | 1.17 |
| 9 | Met | 36.17 | 33.62 | 6.72 | 1.10 |
| 10 | Ile | 46.08 | 37.14 | 14.50 | 1.31 |
| 11 | Leu | 47.09 | 38.29 | 11.57 | 1.31 |
| 12 | Phe | 49.30 | 40.38 | 11.77 | 1.28 |
| 13 | Trp | 48.12 | 43.03 | 7.06 | 1.15 |

Table S5 Isolation of 13 D/L-amino acids labeled by TPP-BSA

Table S6 Calibration curve and detection limit of 14 amino acids (n = 3)

| Amino acids | Calibration range  (μM) | Linear equation | Linearity (R^2^) | LOD |
| --- | --- | --- | --- | --- |
|  |  |  |  | (fmol) |
| D-Ala | 1-25 | y = 0.0218x+ 0.0215 | 0.9966 | 3 |
| L-Ala | 25-1000 | y = 0.0449x - 0.9357 | 0.9992 | 3 |
| D-Phe | 0.1-10 | y = 0.0272x - 0.0017 | 0.9922 | 2 |
| L-Phe | 10-500 | y = 0.0276x - 0.0057 | 0.9984 | 2 |
| D-Asn | 0.1-10 | y = 0.0119x - 0.0009 | 0.9997 | 2 |
| L-Asn | 5-250 | y = 0.0168x - 0.0569 | 0.9934 | 2 |
| D-Thr | 0.05-5 | y = 0.0187x - 0.0012 | 0.9984 | 2 |
| L-Thr | 1-100 | y = 0.0168x+ 0.074 | 0.9974 | 2 |
| D-Met | 0.05-5 | y = 0.0386x - 0.0012 | 0.9992 | 1 |
| L-Met | 1-100 | y = 0.0269x+ 0.1161 | 0.9983 | 1 |
| D-Ile | 0.1-10 | y = 0.0426x - 0.0034 | 0.9997 | 1.5 |
| L-Ile | 10-500 | y = 0.0178x - 0.0522 | 0.9976 | 1.5 |
| D-Leu | 0.1-10 | y = 0.0236x - 0.0008 | 0.9992 | 1.5 |
| L-Leu | 10-500 | y = 0.0176x - 0.1823 | 0.997 | 1.5 |
| D-Tyr | 0.1-10 | y = 0.0236x - 0.0008 | 0.9997 | 2 |
| L-Tyr | 25-1000 | y = 0.0199x + 0.3174 | 0.9971 | 2 |
| D-Val | 0.1-10 | y = 0.0573x - 0.0036 | 0.9998 | 1 |
| L-Val | 25-1000 | y = 0.0298x + 0.6207 | 0.995 | 1 |
| D-Asp | 0.05-5 | y = 0.0054x - 0.0007 | 0.9957 | 4 |
| L-Asp | 1-100 | y = 0.0048x - 0.0065 | 0.9919 | 4 |
| D-Trp | 0.1-10 | y = 0.0265x - 0.0002 | 0.9997 | 2 |
| L-Trp | 10-500 | y = 0.0183x + 0.0272 | 0.9996 | 2 |
| D-His | 0.1-10 | y = 0.0009x - 0.0001 | 0.9936 | 2 |
| L-His | 5-250 | y = 0.0009x - 0.0022 | 0.9915 | 2 |
| D-Glu | 0.05-5 | y = 0.0107 - 0.0002 | 0.9976 | 4.5 |
| L-Glu | 5-250 | y = 0.0095x + 0.064 | 0.9996 | 4.5 |
| Gly | 10-500 | y = 0.0441x + 0.2599 | 0.9914 | 0.2 |

Table S7 Precision and accuracy of 14 amino acids in blank serum (n = 6)

| Amino acids (μM) | Spiked amount (μM) | | Intra-day (n=6) | | | | | | | Inter-day (n=6) | | |  |
| --- | --- | --- | --- | --- | --- | --- | --- | --- | --- | --- | --- | --- | --- |
|  |  |  | Mean (μM) | | RSD (%) | | Accuracy (%) | | | Mean (μM) | RSD (%) | Accuracy (%) |  |
|  |  |  |  |  |  |  |  |  |  |  |  |  |  |
| D-Ala | 2.00 | | 1.90 | | 10.87 | | 94.87 | | | 1.93 | 5.09 | 96.58 |  |
|  | 6.25 | | 6.27 | | 7.35 | | 100.33 | | | 6.44 | 4.68 | 102.98 |  |
|  | 20.00 | | 19.96 | | 4.23 | | 99.78 | | | 19.68 | 3.97 | 98.39 |  |
| L-Ala | 50.00 | | 50.24 | | 4.01 | | 100.48 | | | 49.71 | 2.58 | 99.42 |  |
|  | 200.00 | | 196.26 | | 6.40 | | 98.13 | | | 200.44 | 4.51 | 100.22 |  |
|  | 800.00 | | 763.63 | | 8.03 | | 95.45 | | | 801.04 | 4.31 | 100.13 |  |
| D-Phe | 0.20 | | 0.19 | | 8.21 | | 96.98 | | | 0.18 | 4.52 | 91.98 |  |
|  | 1.25 | | 1.24 | | 6.30 | | 99.45 | | | 1.23 | 5.54 | 98.76 |  |
|  | 8.00 | | 7.95 | | 4.97 | | 99.33 | | | 7.93 | 6.21 | 99.18 |  |
| L-Phe | 20.00 | | 19.74 | | 3.35 | | 98.72 | | | 19.99 | 4.45 | 99.94 |  |
|  | 100.00 | | 97.68 | | 9.37 | | 97.68 | | | 97.62 | 4.03 | 97.62 |  |
|  | 400.00 | | 380.75 | | 7.07 | | 95.19 | | | 396.28 | 3.70 | 99.07 |  |
| D-Asn | 0.20 | | 0.19 | | 3.96 | | 97.26 | | | 0.20 | 2.49 | 98.63 |  |
|  | 1.25 | | 1.21 | | 4.89 | | 97.09 | | | 1.20 | 3.42 | 96.07 |  |
|  | 8.00 | | 7.71 | | 6.02 | | 96.32 | | | 8.00 | 2.74 | 100.03 |  |
| L-Asn | 10.00 | | 10.25 | | 2.39 | | 102.55 | | | 9.98 | 1.19 | 99.82 |  |
|  | 40.00 | | 38.99 | | 4.55 | | 97.48 | | | 39.59 | 3.02 | 98.98 |  |
|  | 200.00 | | 196.78 | | 5.31 | | 98.39 | | | 198.84 | 2.98 | 99.42 |  |
| D-Thr | 0.10 | | 0.10 | | 1.44 | | 98.01 | | | 0.10 | 0.92 | 98.98 |  |
|  | 0.80 | | 0.76 | | 4.74 | | 95.03 | | | 0.75 | 3.84 | 93.45 |  |
|  | 4.00 | | 3.87 | | 9.20 | | 96.85 | | | 3.86 | 4.02 | 96.61 |  |
| L-Thr | 2.00 | | 1.99 | | 8.37 | | 99.48 | | | 1.89 | 12.87 | 94.52 |  |
|  | 20.00 | | 19.92 | | 10.33 | | 99.59 | | | 19.97 | 5.22 | 99.86 |  |
|  | 80.00 | | 76.81 | | 8.08 | | 96.01 | | | 79.97 | 3.71 | 99.96 |  |
| D-Met | 0.10 | | 0.09 | | 4.61 | | 93.87 | | | 0.10 | 3.22 | 98.24 |  |
|  | 0.80 | | 0.75 | | 6.87 | | 94.14 | | | 0.76 | 5.60 | 94.78 |  |
|  | 4.00 | | 3.98 | | 8.87 | | 99.43 | | | 3.88 | 4.28 | 96.92 |  |
| L-Met | 2.00 | | 1.91 | | 2.17 | | 95.41 | | | 1.85 | 6.82 | 92.57 |  |
|  | 20.00 | | 19.59 | | 7.53 | | 97.95 | | | 19.81 | 1.64 | 99.06 |  |
|  | | 80.00 | | 80.71 | | 6.84 | | 100.89 | 79.82 | | 4.84 | 99.78 |  |
| D-Ile | | 0.20 | | 0.19 | | 2.01 | | 97.21 | 0.20 | | 2.27 | 98.55 |  |
|  |  | 1.25 | | 1.21 | | 6.43 | | 96.49 | 1.21 | | 3.14 | 96.75 |  |
|  |  | 8.00 | | 8.33 | | 5.84 | | 104.09 | 8.01 | | 4.56 | 100.07 |  |
| L-Ile | | 20.00 | | 19.86 | | 4.92 | | 99.30 | 19.82 | | 3.87 | 99.09 |  |
|  |  | 100.00 | | 97.11 | | 6.96 | | 97.11 | 98.29 | | 2.48 | 98.29 |  |
|  |  | 400.00 | | 392.10 | | 6.22 | | 98.02 | 394.71 | | 4.89 | 98.68 |  |
| D-Leu | | 0.20 | | 0.20 | | 5.13 | | 99.92 | 0.20 | | 3.24 | 97.91 |  |
|  |  | 1.25 | | 1.24 | | 4.83 | | 99.42 | 1.22 | | 3.29 | 97.59 |  |
|  |  | 8.00 | | 8.39 | | 8.45 | | 104.89 | 8.04 | | 3.64 | 100.46 |  |
| L-Leu | | 20.00 | | 20.06 | | 3.39 | | 100.31 | 20.00 | | 1.83 | 99.98 |  |
|  |  | 100.00 | | 99.15 | | 5.60 | | 99.15 | 98.15 | | 4.18 | 98.15 |  |
|  |  | 400.00 | | 381.35 | | 7.12 | | 95.34 | 390.77 | | 3.02 | 97.69 |  |
| D-Tyr | | 0.20 | | 0.21 | | 4.18 | | 103.76 | 0.20 | | 2.82 | 98.16 |  |
|  |  | 1.25 | | 1.21 | | 6.68 | | 96.80 | 1.22 | | 3.94 | 97.62 |  |
|  |  | 8.00 | | 7.92 | | 9.65 | | 99.00 | 8.05 | | 3.02 | 100.63 |  |
| L-Tyr | | 50.00 | | 49.80 | | 7.07 | | 99.60 | 49.67 | | 4.06 | 99.35 |  |
|  |  | 200.00 | | 200.43 | | 5.99 | | 100.22 | 200.19 | | 4.05 | 100.10 |  |
|  |  | 800.00 | | 774.65 | | 6.65 | | 96.83 | 792.94 | | 4.96 | 99.12 |  |
| D-Val | | 0.20 | | 0.19 | | 3.11 | | 96.97 | 0.20 | | 2.34 | 97.82 |  |
|  |  | 1.25 | | 1.22 | | 7.71 | | 97.63 | 1.21 | | 3.41 | 96.96 |  |
|  |  | 8.00 | | 7.89 | | 6.21 | | 98.67 | 8.05 | | 2.63 | 100.67 |  |
| L-Val | | 50.00 | | 49.10 | | 7.87 | | 98.21 | 49.33 | | 5.95 | 98.65 |  |
|  |  | 200.00 | | 191.69 | | 4.63 | | 95.84 | 200.00 | | 5.49 | 100.00 |  |
|  |  | 800.00 | | 793.20 | | 5.83 | | 99.15 | 788.16 | | 5.44 | 98.52 |  |
| D-Asp | | 0.10 | | 0.09 | | 5.06 | | 94.10 | 0.10 | | 3.20 | 103.50 |  |
|  |  | 0.80 | | 0.81 | | 6.92 | | 101.50 | 0.85 | | 3.09 | 105.94 |  |
|  |  | 4.00 | | 4.19 | | 4.79 | | 104.67 | 4.28 | | 2.61 | 106.97 |  |
| L-Asp | | 2.00 | | 1.85 | | 5.08 | | 92.42 | 1.89 | | 3.80 | 94.37 |  |
|  |  | 20.00 | | 19.30 | | 3.14 | | 96.52 | 19.15 | | 13.46 | 95.74 |  |
|  |  | 80.00 | | 83.18 | | 2.33 | | 103.98 | 82.20 | | 3.33 | 102.75 |  |
| D-Trp | | 0.20 | | 0.19 | | 6.49 | | 93.50 | 0.19 | | 7.33 | 97.00 |  |
|  |  | 1.25 | | 1.25 | | 4.88 | | 99.67 | 1.21 | | 2.82 | 97.19 |  |
|  |  | 8.00 | | 8.47 | | 8.19 | | 105.82 | 8.01 | | 3.99 | 100.14 |  |
| L-Trp | | 20.00 | | 18.36 | | 4.27 | | 91.82 | 19.93 | | 3.43 | 99.64 |  |
|  |  | 100.00 | | 95.66 | | 5.46 | | 95.66 | 98.15 | | 4.18 | 98.15 |  |
|  |  | 400.00 | | 404.19 | | 7.39 | | 101.05 | 394.71 | | 3.48 | 98.68 |  |
| D-His | | 0.20 | | 0.20 | | 3.26 | | 101.03 | 0.20 | | 1.04 | 99.10 |  |
|  |  | 1.25 | | 1.19 | | 6.95 | | 95.29 | 1.22 | | 3.94 | 97.62 |  |
|  |  | 8.00 | | 7.92 | | 4.05 | | 99.00 | 7.91 | | 4.05 | 98.83 |  |
| L-His | | 10.00 | | 9.59 | | 5.95 | | 95.87 | 9.84 | | 2.73 | 98.38 |  |
|  |  | 40.00 | | 39.86 | | 7.16 | | 99.64 | 39.38 | | 3.19 | 98.44 |  |
|  |  | 200.00 | | 190.11 | | 7.18 | | 95.05 | 192.73 | | 5.29 | 96.36 |  |
| D-Glu | | 0.10 | | 0.11 | | 2.12 | | 108.06 | 0.09 | | 14.00 | 94.35 |  |
|  |  | 0.80 | | 0.82 | | 3.40 | | 102.04 | 0.81 | | 4.38 | 101.55 |  |
|  |  | 4.00 | | 4.16 | | 5.83 | | 103.95 | 4.05 | | 4.58 | 101.19 |  |
| L-Glu | | 10.00 | | 10.32 | | 7.08 | | 103.22 | 9.67 | | 6.72 | 96.74 |  |
|  |  | 40.00 | | 37.32 | | 7.74 | | 93.30 | 38.95 | | 4.34 | 97.37 |  |
|  |  | 200.00 | | 202.71 | | 6.05 | | 101.36 | 199.48 | | 4.57 | 99.74 |  |
| Gly | | 20.00 | | 19.84 | | 3.46 | | 99.20 | 21.12 | | 4.56 | 105.58 |  |
|  |  | 100.00 | | 96.99 | | 6.91 | | 96.99 | 97.60 | | 2.92 | 97.60 |  |
|  |  | 400 | | 385.95 | | 7.31 | | 96.49 | 391.52 | | 2.73 | 97.88 |  |

Table S8 Recovery of 14 amino acids spiked in artificial serum (n = 6)

| Amino acids | Spiked amount (μM) | Measured  Mean ± SD (μM) | RSD (%) (n=6) | Recovery (%) | Mean Recovery (%) |
| --- | --- | --- | --- | --- | --- |
|  |  |  |  |  |  |
|  |  |  |  |  |  |
| D-Ala | 2 | 2.078±0.190 | 9.13 | 103.9 | 102.86 |
|  | 6.25 | 6.465±0.315 | 4.88 | 103.44 |  |
|  | 20 | 20.25±1.754 | 8.66 | 101.25 |  |
| L-Ala | 50 | 50.028±3.965 | 7.93 | 100.06 | 99.56 |
|  | 200 | 196.505±7.139 | 3.63 | 98.25 |  |
|  | 800 | 803.006±69.814 | 8.69 | 100.38 |  |
| D-Phe | 0.2 | 0.201±0.007 | 3.39 | 100.50 | 100.68 |
|  | 1.25 | 1.246±0.064 | 5.17 | 99.68 |  |
|  | 8 | 8.148±0.451 | 5.53 | 101.85 |  |
| L-Phe | 20 | 21.117±0.849 | 4.02 | 105.59 | 103.90 |
|  | 100 | 100.13±5.623 | 5.62 | 100.13 |  |
|  | 400 | 423.984±25.089 | 5.92 | 106.00 |  |
| D-Asn | 0.2 | 0.202±0.009 | 4.64 | 101.00 | 101.40 |
|  | 1.25 | 1.26±0.047 | 3.76 | 100.80 |  |
|  | 8 | 8.192±0.717 | 8.75 | 102.40 |  |
| L-Asn | 10 | 10.052±0.756 | 7.52 | 100.52 | 100.97 |
|  | 40 | 39.725±1.529 | 3.85 | 99.31 |  |
|  | 200 | 206.162±10.876 | 5.28 | 103.08 |  |
| D-Thr | 0.1 | 0.099±0.002 | 2.33 | 99.00 | 100.36 |
|  | 0.8 | 0.788±0.023 | 2.92 | 98.50 |  |
|  | 4 | 4.143±0.329 | 7.94 | 103.58 |  |
| L-Thr | 2 | 1.896±0.449 | 11.59 | 93.79 | 99.27 |
|  | 20 | 20.179±0.935 | 4.63 | 100.90 |  |
|  | 80 | 82.491±7.26 | 8.80 | 103.11 |  |
| D-Met | 0.1 | 0.101±0.005 | 5.22 | 101.00 | 99.99 |
|  | 0.8 | 0.803±0.031 | 3.81 | 100.38 |  |
|  | 4 | 3.944±0.167 | 4.23 | 98.60 |  |
| L-Met | 2 | 1.99±0.921 | 11.80 | 99.50 | 101.32 |
|  | 20 | 20.295±1.34 | 6.60 | 101.48 |  |
|  | 80 | 82.385±10.670 | 12.95 | 102.98 |  |
| D-Ile | 0.2 | 0.198±0.007 | 3.52 | 99.00 | 99.36 |
|  | 1.25 | 1.246±0.046 | 3.71 | 99.68 |  |
|  | 8 | 7.951±0.403 | 5.07 | 99.39 |  |
| L-Ile | 20 | 19.934±0.877 | 4.40 | 99.67 | 99.22 |
|  | 100 | 98.922±2.116 | 2.14 | 98.92 |  |
|  | 400 | 396.215±35.635 | 8.99 | 99.05 |  |
| D-Leu | 0.2 | 0.191±0.010 | 5.30 | 95.50 | 99.58 |
|  | 1.25 | 1.262±0.070 | 5.58 | 100.96 |  |
|  | 8 | 8.182±0.703 | 8.59 | 102.28 |  |
| L-Leu | 20 | 19.599±0.538 | 2.74 | 98.00 | 98.08 |
|  | 100 | 101.717±2.686 | 2.64 | 101.72 |  |
|  | 400 | 378.09±18.873 | 4.99 | 94.52 |  |
| D-Tyr | 0.2 | 0.214±0.017 | 8.04 | 107.00 | 102.85 |
|  | 1.25 | 1.242±0.044 | 3.57 | 99.36 |  |
|  | 8 | 8.174±0.547 | 6.70 | 102.18 |  |
| L-Tyr | 50 | 53.925±6.893 | 12.78 | 107.85 | 102.91 |
|  | 200 | 197.239±7.663 | 3.89 | 98.62 |  |
|  | 800 | 818.009±56.428 | 6.90 | 102.25 |  |
| D-Val | 0.2 | 0.205±0.014 | 6.64 | 102.50 | 101.06 |
|  | 1.25 | 1.255±0.059 | 5.96 | 100.40 |  |
|  | 8 | 8.023±0.662 | 3.42 | 100.29 |  |
| L-Val | 50 | 49.134±6.617 | 13.47 | 98.27 | 98.37 |
|  | 200 | 198.454±10.361 | 5.22 | 99.23 |  |
|  | 800 | 780.981±67.805 | 8.68 | 97.62 |  |
| D-Asp | 0.10 | 0.093±0.007 | 7.19 | 93.00 | 97.88 |
|  | 0.80 | 0.813±0.046 | 5.71 | 101.63 |  |
|  | 4.00 | 3.961±0.189 | 4.78 | 99.03 |  |
| L-Asp | 2 | 1.876±0.175 | 9.32 | 93.80 | 98.71 |
|  | 20 | 20.311±1.020 | 5.02 | 101.56 |  |
|  | 80 | 80.609±6.972 | 8.65 | 100.76 |  |
| D-Trp | 0.2 | 0.197±0.015 | 7.64 | 98.50 | 99.20 |
|  | 1.25 | 1.28±0.059 | 4.59 | 102.40 |  |
|  | 8 | 7.737±0.662 | 8.55 | 96.71 |  |
| L-Trp | 20 | 20.494±1.893 | 9.24 | 102.47 | 100.09 |
|  | 100 | 99.36±4.943 | 4.97 | 99.36 |  |
|  | 400 | 393.725±32.121 | 8.16 | 98.43 |  |
| D-His | 0.2 | 0.203±0.007 | 3.65 | 101.50 | 99.93 |
|  | 1.25 | 1.26±0.024 | 1.88 | 100.80 |  |
|  | 8 | 7.8±0.497 | 6.37 | 97.50 |  |
| L-His | 10 | 10.528±0.842 | 7.99 | 105.28 | 101.41 |
|  | 40 | 39.674±1.617 | 4.08 | 99.19 |  |
|  | 200 | 199.511±0.497 | 10.05 | 99.76 |  |
| D-Glu | 0.1 | 0.113±0.004 | 3.54 | 113.00 | 111.78 |
|  | 0.8 | 0.897±0.039 | 4.35 | 112.13 |  |
|  | 4 | 4.408±0.282 | 6.39 | 110.20 |  |
| L-Glu | 10 | 10.534±1.02 | 9.68 | 105.34 | 100.88 |
|  | 40 | 39.24±2.432 | 6.20 | 98.10 |  |
|  | 200 | 198.416±9.956 | 5.02 | 99.21 |  |
| Gly | 20 | 19.038±2.318 | 12.18 | 95.19 | 99.34 |
|  | 100 | 100.875±3.446 | 3.42 | 100.88 |  |
|  | 400 | 407.823±36.795 | 9.02 | 101.96 |  |

Table S9 Matrix effect of 14 amino acids in artificial serum (n = 6)

| Amino acids | Spiked amount (μM) | Matrix effect (%) | |
| --- | --- | --- | --- |
|  |  | Mean ± SD | RSD (%) |
| D-Ala | 2 | 95.21± 8.2 | 8.61 |
|  | 20 | 103.73± 5.59 | 5.39 |
| L-Ala | 50 | 101.18± 10.68 | 10.56 |
|  | 800 | 96.67± 7.99 | 8.26 |
| D-Phe | 0.2 | 91.9± 5.93 | 6.46 |
|  | 8 | 105.85± 4.76 | 4.5 |
| L-Phe | 20 | 101.81± 11.91 | 11.7 |
|  | 400 | 88.22± 4.76 | 9.01 |
| D-Asn | 0.2 | 104.29± 8.75 | 8.39 |
|  | 8 | 102.91± 8.68 | 8.44 |
| L-Asn | 10 | 101.6± 12.14 | 11.95 |
|  | 200 | 101.14± 12.23 | 12.09 |
| D-Thr | 0.1 | 93.83± 8.75 | 8.37 |
|  | 4 | 98.94± 9.12 | 9.22 |
| L-Thr | 2 | 103.7± 13.66 | 13.17 |
|  | 80 | 98.88± 8.61 | 8.71 |
| D-Met | 0.1 | 96.52± 13.58 | 14.07 |
|  | 4 | 100.65± 8.18 | 8.13 |
| L-Met | 2 | 106.7± 7.78 | 7.3 |
|  | 80 | 100.58± 7.79 | 7.74 |
| D-Ile | 0.2 | 98.59± 9.82 | 9.96 |
|  | 8 | 100.83± 5.78 | 5.73 |
| L-Ile | 20 | 107± 8.08 | 7.55 |
|  | 400 | 97.27± 7.9 | 8.12 |
| D-Leu | 0.2 | 101.35± 11.16 | 11.02 |
|  | 8 | 101.54± 12.29 | 12.11 |
| L-Leu | 20 | 103.95± 11.77 | 11.32 |
|  | 400 | 96.88± 9.37 | 9.67 |
| D-Tyr | 0.2 | 109.37± 12.6 | 11.52 |
|  | 8 | 103.4± 7.66 | 7.4 |
| L-Tyr | 50 | 104.47± 9.32 | 8.92 |
|  | 800 | 93.07± 6.6 | 7.09 |
| D-Val | 0.2 | 96.85± 10.27 | 10.6 |
|  | 8 | 94.71± 5.02 | 5.3 |
| L-Val | 50 | 96.73± 8.36 | 8.65 |
|  | 800 | 98.42± 9.29 | 9.44 |
| D-Asp | 0.1 | 97.55± 9.61 | 9.85 |
|  | 4 | 104.47± 9.96 | 9.54 |
| L-Asp | 2 | 98.68± 11.07 | 11.22 |
|  | 80 | 106.34± 12.21 | 11.48 |
| D-Trp | 0.2 | 103.95± 9.34 | 8.98 |
|  | 8 | 106.85± 5.37 | 5.03 |
| L-Trp | 20 | 99.49± 4.38 | 4.4 |
|  | 400 | 104.29± 8.15 | 7.82 |
| D-His | 0.2 | 103.42± 12.52 | 12.1 |
|  | 8 | 101.87± 9.52 | 9.35 |
| L-His | 10 | 96.02± 3.67 | 3.82 |
|  | 200 | 104.47± 14.6 | 13.97 |
| D-Glu | 0.1 | 107.91± 3.67 | 14.58 |
|  | 8 | 97.87± 11.27 | 11.52 |
| L-Glu | 10 | 100.15± 14.03 | 14.01 |
|  | 200 | 101.06± 9.24 | 9.14 |
| Gly | 20 | 103.55± 10.17 | 9.82 |
|  | 400 | 91.72± 5.9 | 6.43 |

Table S10 Stability test results of 14 amino acids in artificial serum (n = 3)

| Amino acids | Spiked amount (μM) | Room temperature for 4 h | | Processed samples in autosampler vials for 8 h | | Three freeze thaw cycles | |
| --- | --- | --- | --- | --- | --- | --- | --- |
|  |  | RE (%) | RSD (%) | RE (%) | RSD (%) | RE (%) | RE (%) |
| D-Ala | 2 | 1.5 | 6 | 11 | 6.8 | 5.5 | 2.3 |
|  | 6.3 | -1.9 | 2.4 | -0.8 | 8.5 | -0.6 | 0.2 |
|  | 20 | -0.7 | 4.1 | 0.2 | 7.5 | 8.8 | 1.8 |
| L-Ala | 50 | 2.1 | 2.9 | 4 | 3.7 | 1.5 | 4.8 |
|  | 200 | 3.6 | 3.1 | -1.8 | 3.2 | 5 | 3.9 |
|  | 800 | -2.1 | 1.1 | 5.1 | 2.7 | -4.5 | 3 |
| D-Phe | 0.2 | 1.6 | 4.2 | 1 | 3 | 8 | 1.1 |
|  | 1.3 | -1.6 | 6.2 | 4.8 | 6.3 | 3.2 | 3.9 |
|  | 8 | 4 | 3.5 | 3 | 7.7 | 4.6 | 3.9 |
| L-Phe | 20 | 4.9 | 7.3 | 10.9 | 4.6 | 3.9 | 4.7 |
|  | 100 | 3.3 | 4.4 | 5 | 6.6 | 2.8 | 5.3 |
|  | 400 | 4.8 | 6.1 | 2.4 | 1.4 | 6.3 | 4.9 |
| D-Asn | 0.2 | 1.1 | 2.4 | 5 | 0.5 | 5 | 1.5 |
|  | 1.3 | 2.4 | 4.1 | 0.8 | 4.1 | 0.8 | 2.4 |
|  | 8 | 1.4 | 4 | 6.9 | 8.3 | 1.5 | 3.7 |
| L-Asn | 10 | -0.7 | 4.8 | 0.8 | 6.4 | 5 | 3.8 |
|  | 40 | 4 | 4.4 | -2.8 | 2.4 | 6.9 | 2 |
|  | 200 | 0.1 | 6.6 | 3.9 | 5.1 | 0.2 | 6.8 |
| D-Thr | 0.1 | 2 | 1.9 | 6 | 0.9 | 12 | 1.3 |
|  | 0.8 | 2.5 | 0.3 | 0.3 | 0.7 | 3.7 | 6.9 |
|  | 4 | 1.8 | 4.3 | -4.3 | 3.5 | 3.3 | 3 |
| L-Thr | 2 | 12.5 | 12.6 | 3 | 1.7 | -4 | 6.8 |
|  | 20 | 2.1 | 4.1 | -0.5 | 3.7 | 1.3 | 8.3 |
|  | 80 | 1.2 | 4.6 | 1.3 | 3.1 | -2.2 | 3.7 |
| D-Met | 0.1 | 5.2 | 4.1 | 6.8 | 5 | 8.8 | 3.7 |
|  | 0.8 | 2.5 | 5.9 | 6.2 | 4.6 | 2.5 | 3.8 |
|  | 4 | 4.3 | 2.4 | 5.8 | 4.4 | -0.5 | 2.4 |
| L-Met | 2 | 8 | 9.9 | 6 | 13.9 | 13.5 | 7.6 |
|  | 20 | 7 | 8.2 | -3.3 | 3.7 | 2.1 | 11.2 |
|  | 80 | -6.2 | 1.6 | -1 | 1.4 | 4.6 | 1.9 |
| D-Ile | 0.2 | 0.6 | 4.5 | 5 | 3 | 6.6 | 3.7 |
|  | 1.3 | 0.8 | 6.8 | 2.4 | 10.2 | -2.4 | 2.4 |
|  | 8 | -0.7 | 8.3 | 1.6 | 7.8 | 7.1 | 5.4 |
| L-Ile | 20 | -3.5 | 5.4 | 5.1 | 2.2 | 3.2 | 4.3 |
|  | 100 | 5.1 | 4.6 | 5.3 | 3.7 | 2.4 | 3.4 |
|  | 400 | 1.9 | 4.1 | 4.3 | 5.7 | -1.5 | 7 |
| D-Leu | 0.2 | 5 | 3.8 | 1.7 | 5.1 | 0.8 | 3.4 |
|  | 1.3 | 5.6 | 1.3 | -1.6 | 4.5 | -1.6 | 3.4 |
|  | 8 | -0.6 | 0.9 | 3 | 3.1 | 7.1 | 3.9 |
| L-Leu | 20 | 3.1 | 2.3 | -0.7 | 0.8 | 1.3 | 1.9 |
|  | 100 | 4.1 | 5.5 | 3 | 1.1 | -0.3 | 5.6 |
|  | 400 | -0.7 | 4 | -2.2 | 5 | -0.6 | 6 |
| D-Tyr | 0.2 | 0 | 3.1 | 4.4 | 2.8 | 5 | 3.5 |
|  | 1.3 | 3.2 | 5.2 | -1.6 | 1.8 | 1.6 | 5.1 |
|  | 8 | 2.1 | 4.6 | 7.4 | 6.1 | 7.3 | 4.6 |
| L-Tyr | 50 | 2.2 | 5.9 | -4 | 8.2 | -3.5 | 6.5 |
|  | 200 | -1.3 | 4.6 | 8.8 | 2.6 | 0.4 | 4.2 |
|  | 800 | 5.5 | 1.4 | 4.6 | 2.2 | 8.1 | 6.5 |
| D-Val | 0.2 | 1.7 | 2.4 | 3.2 | 4.6 | 6.1 | 2 |
|  | 1.3 | 3.2 | 5.1 | 4 | 4.6 | 4.8 | 5.2 |
|  | 8 | 8.7 | 3.2 | -0.2 | 7 | -1.4 | 4 |
| L-Val | 50 | 1.5 | 10.9 | -0.2 | 9.2 | 5.4 | 7.2 |
|  | 200 | 1.9 | 3.4 | -0.3 | 4.3 | 3.5 | 4.1 |
|  | 800 | 5.7 | 3.4 | 0.5 | 4.2 | 2.3 | 4.7 |
| D-Asp | 0.1 | 0.3 | 2.9 | 0.6 | 0.9 | 5 | 1.7 |
|  | 0.8 | -1.3 | 6.3 | 8.7 | 4.3 | 2.5 | 5.9 |
|  | 4 | -2.5 | 3.3 | -1.3 | 7 | 10.3 | 1.2 |
| L-Asp | 2 | 11.5 | 10.7 | 8.5 | 10.6 | -1 | 11.3 |
|  | 20 | 1.9 | 3.7 | -0.9 | 8.6 | 1.5 | 5.8 |
|  | 80 | 5.7 | 6.3 | -1.3 | 6.2 | 6.6 | 6.1 |
| D-Trp | 0.2 | 5 | 3.2 | 5 | 5.5 | 1.7 | 4.1 |
|  | 1.3 | 2.4 | 5.2 | 1.6 | 0.3 | 5.6 | 0.9 |
|  | 8 | 1.5 | 4.6 | 10.1 | 3.6 | 6.5 | 5.6 |
| L-Trp | 20 | 6.1 | 2.2 | -3.7 | 3.6 | 5.7 | 3.2 |
|  | 100 | 4.1 | 2.4 | 3.3 | 4.7 | 3.5 | 9.5 |
|  | 400 | 1 | 3.5 | 0.2 | 5.3 | 2.9 | 2.1 |
| D-His | 0.2 | 0.5 | 1.2 | 5 | 1.8 | 1.2 | 0.6 |
|  | 1.3 | 3.2 | 3.9 | 1.6 | 7.1 | -0.8 | 3.5 |
|  | 8 | -2 | 5.1 | 2.1 | 4.7 | 0.2 | 5.9 |
| L-His | 10 | 5.4 | 1.3 | 1.5 | 7.8 | -3.4 | 1.1 |
|  | 40 | 0.4 | 7.1 | 3.6 | 4.8 | 2.9 | 6.5 |
|  | 200 | 2.6 | 5.8 | 6.2 | 7.2 | 5.2 | 9.4 |
| D-Glu | 0.1 | -10 | 1.4 | 3 | 1.6 | -8.3 | 1.2 |
|  | 0.8 | 0 | 4 | 3.7 | 1.9 | -10.8 | 2.3 |
|  | 4 | 4.5 | 4.3 | 7.5 | 4.6 | 5.5 | 5.2 |
| L-Glu | 10 | 2.5 | 6.2 | 1.8 | 3.7 | 2.5 | 14.6 |
|  | 40 | 1.1 | 3.8 | 2 | 1.1 | 0.9 | 7.3 |
|  | 200 | 0.2 | 4.4 | 4.9 | 4.1 | 5.7 | 1.8 |
| Gly | 20 | 2.9 | 7.4 | 3.1 | 4.3 | 3.2 | 10.5 |
|  | 100 | 6.3 | 4.3 | -2.7 | 4.3 | 2 | 8.7 |
|  | 400 | 5.3 | 8 | 7.2 | 7.7 | 6.8 | 4.4 |

Table S11 Content of 14 L-amino acids in serum of healthy volunteers

| Sex | Age | L-Ala | L-Phe | L-Asn | L-Thr | L-Met | L-Ile | L-Leu | L-Tyr | L-Val | L-Asp | L-Trp | L-His | L-Glu | Gly |
| --- | --- | --- | --- | --- | --- | --- | --- | --- | --- | --- | --- | --- | --- | --- | --- |
|  |  | (μM) | (μM) | (μM) | (μM) | (μM) | (μM) | (μM) | (μM) | (μM) | (μM) | (μM) | (μM) | (μM) | (μM) |
| M | 79 | 409.09 | 137.3 | 44.04 | 81.51 | 22.16 | 108.51 | 190.66 | 46.59 | 272.72 | 43.63 | 20.83 | 127.16 | 109.69 | 183.67 |
| M | 69 | 545.23 | 81.03 | 68.91 | 112.02 | 24.34 | 94.28 | 153.74 | 63.5 | 238.59 | 39.89 | 39.08 | 147.26 | 97.69 | 181.59 |
| M | 67 | 394.46 | 65.73 | 65.03 | 87.36 | 18.83 | 65.39 | 119.58 | 59.37 | 188.38 | 46.36 | 47.03 | 112.87 | 80.99 | 227.21 |
| M | 64 | 344.92 | 94.56 | 68.39 | 105.3 | 25.33 | 136.95 | 211.53 | 55.32 | 234.4 | 35.16 | 21.43 | 100.33 | 72.76 | 181.92 |
| M | 61 | 68.63 | 100.74 | 51.7 | 103.31 | 17.34 | 68.46 | 151.05 | 64.87 | 231.58 | 45.05 | 65.99 | 133.6 | 113.88 | 171.87 |
| M | 68 | 684.45 | 85.69 | 68.75 | 135.23 | 28.73 | 95.59 | 194.5 | 80.41 | 295.1 | 49.11 | 66.25 | 146.73 | 113.18 | 184.55 |
| M | 69 | 547.43 | 92.6 | 41.33 | 82.05 | 17.41 | 78.8 | 151.13 | 55.13 | 220.51 | 59.13 | 54.5 | 105.12 | 132.52 | 147.36 |
| M | 62 | 505.8 | 75.15 | 54.06 | 149.35 | 23.89 | 70.6 | 121.66 | 54.8 | 222.82 | 33.52 | 48.48 | 105.58 | 63.5 | 202.11 |
| M | 70 | 463.99 | 69.68 | 39.84 | 63.21 | 15.26 | 69.89 | 153.23 | 51.82 | 220.24 | 34.2 | 50.39 | 118 | 95.62 | 124.56 |
| M | 59 | 325.83 | 107.56 | 41.52 | 66.35 | 12.57 | 59.99 | 125.84 | 44.4 | 199.25 | 58.35 | 33 | 84.09 | 122.91 | 151.54 |
| M | 65 | 224.38 | 44.08 | 47.76 | 39.67 | 33.92 | 45.41 | 42.8 | 43.33 | 162.26 | 34.41 | 35.82 | 134.12 | 49.68 | 123.17 |
| M | 55 | 285.25 | 55.89 | 28.43 | 57.65 | 11.28 | 57.88 | 112.21 | 32.38 | 171.77 | 18.88 | 43.57 | 86.55 | 56.7 | 113.68 |
| M | 58 | 390.2 | 63.42 | 40.51 | 67.43 | 13.37 | 61.23 | 127.5 | 39.81 | 206.95 | 28.21 | 51.92 | 105.35 | 68.21 | 140.19 |
| M | 63 | 392.32 | 63.06 | 36.26 | 59.93 | 13.24 | 55.65 | 113.05 | 39.38 | 177.32 | 28.67 | 47.76 | 92.95 | 62.09 | 133.31 |
| M | 55 | 289.7 | 76.23 | 38.82 | 77.89 | 13.72 | 82.67 | 162.55 | 45.82 | 222.65 | 41.36 | 39.88 | 100.55 | 137.43 | 129.22 |
| M | 63 | 520.22 | 70.36 | 23.87 | 62.86 | 16.08 | 78.35 | 170.24 | 55.02 | 231.75 | 26.9 | 47.69 | 35.59 | 50.57 | 136.96 |
| M | 77 | 262.36 | 85.88 | 19.8 | 31.62 | 10.69 | 59.47 | 101.33 | 28.92 | 152.68 | 29.25 | 27.67 | 15.86 | 44.41 | 90.96 |
| M | 59 | 404.92 | 63.03 | 29.92 | 86.62 | 13.91 | 72.36 | 139.11 | 38.76 | 219.78 | 32.41 | 58.93 | 31.33 | 58.43 | 161.38 |
| M | 67 | 405.65 | 62.92 | 29.96 | 87 | 13.92 | 72.47 | 137.8 | 38.82 | 220.16 | 33.28 | 59.02 | 32.48 | 61.41 | 161.56 |
| M | 62 | 364.38 | 67.39 | 32.71 | 54.74 | 12.18 | 99.96 | 146.6 | 28.39 | 227.84 | 62.74 | 33.4 | 28.12 | 79.3 | 259.97 |
| M | 67 | 405.21 | 63.78 | 30.58 | 74.89 | 19.68 | 85.8 | 149 | 47 | 212.57 | 27.79 | 47.99 | 31.27 | 67.17 | 141.33 |
| M | 70 | 575.74 | 102.63 | 42.68 | 64.11 | 17.04 | 69.63 | 151.21 | 51.69 | 239.97 | 97.11 | 38.36 | 30.58 | 229.83 | 165.65 |
| M | 83 | 424.8 | 87.21 | 30.05 | 68.38 | 14.12 | 81.36 | 182.66 | 61.7 | 289.87 | 55.49 | 49.12 | 28.89 | 86.44 | 132.75 |
| M | 58 | 266.22 | 105.82 | 29.34 | 46.46 | 7.9 | 66.72 | 144.09 | 32.67 | 204.13 | 20.37 | 22.02 | 29.37 | 62.48 | 135.67 |
| M | 62 | 357.08 | 147 | 35.17 | 42.5 | 13.47 | 192.36 | 159.77 | 44.39 | 169.86 | 50.06 | 19.59 | 60.62 | 68.76 | 185.3 |
| M | 67 | 310.44 | 52.91 | 26.06 | 48.39 | 11.67 | 57.62 | 115.13 | 26.84 | 182.75 | 31.38 | 40.53 | 33.51 | 53.65 | 141.36 |
| M | 70 | 587.7 | 62.21 | 32.82 | 82.09 | 15.96 | 77.27 | 146.28 | 41.43 | 230.4 | 49.33 | 49.26 | 40.71 | 89.57 | 193.79 |
| M | 83 | 403.37 | 62.77 | 38.59 | 89.57 | 13.3 | 52.11 | 130.68 | 40.69 | 214.89 | 51.58 | 51.72 | 44.71 | 121.89 | 214.09 |
| M | 58 | 390.16 | 60.16 | 26.91 | 47.07 | 11.03 | 68.48 | 145.73 | 46.94 | 231.66 | 27.79 | 49.7 | 41.68 | 85.67 | 139.78 |
| M | 61 | 543.73 | 70.56 | 42.56 | 104.83 | 20.39 | 111.62 | 176.36 | 48.78 | 265.86 | 46.52 | 57.42 | 44.58 | 68.81 | 179.33 |
| M | 66 | 659.12 | 112.76 | 68.38 | 136.6 | 24.27 | 88.37 | 154.5 | 64.14 | 216.4 | 28.85 | 40.25 | 54.53 | 114.07 | 205.04 |
| M | 69 | 810.49 | 93.62 | 42.14 | 73.27 | 6.66 | 153.51 | 224.13 | 45.46 | 311.66 | 74.17 | 52.46 | 46.32 | 143.87 | 234.01 |
| M | 67 | 661.7 | 127.27 | 34.57 | 153.37 | 6.62 | 126.48 | 241.64 | 73.81 | 357.69 | 57.03 | 89.4 | 66.69 | 66.85 | 325.6 |
| M | 63 | 587.36 | 104.61 | 50.77 | 147.86 | 7.53 | 120.74 | 212.79 | 66.99 | 322.85 | 62.88 | 71.26 | 56.4 | 214.48 | 346.27 |
| M | 62 | 759.35 | 110.39 | 19.25 | 93.88 | 6.72 | 122.08 | 206.78 | 48.38 | 296.14 | 98.13 | 38.25 | 48.83 | 70.1 | 322.89 |
| M | 79 | 309.53 | 46.08 | 28.36 | 64.02 | 12.43 | 60.64 | 73.71 | 38.45 | 134.26 | 51.49 | 47.81 | 26.18 | 65.78 | 172.19 |
| M | 80 | 665.11 | 131.65 | 93.84 | 134.83 | 30.15 | 85.77 | 159.42 | 83.6 | 180.14 | 35.35 | 26.9 | 70.55 | 122.96 | 335.17 |
| M | 76 | 218.11 | 38.34 | 20.56 | 29.51 | 10.34 | 14.35 | 41.18 | 38.26 | 160.27 | 10.68 | 26.29 | 25.18 | 22.61 | 78.41 |
| M | 75 | 276.48 | 54.49 | 25.66 | 46.34 | 11.56 | 25.07 | 67.16 | 47.06 | 100.82 | 20.97 | 35.42 | 26.02 | 65.53 | 91.13 |
| M | 70 | 396.51 | 41.87 | 24.07 | 54.01 | 8.72 | 49.93 | 66.41 | 27.8 | 135.5 | 41.69 | 29.7 | 20.73 | 47.91 | 155.58 |
| M | 71 | 358.35 | 59.34 | 31.09 | 89.22 | 11.5 | 67.78 | 122.02 | 35.38 | 212.23 | 49.08 | 40.39 | 41.99 | 81.21 | 170.67 |
| F | 69 | 312.77 | 47.42 | 35.76 | 68.72 | 8.61 | 34.95 | 77.89 | 29.34 | 126.91 | 37.08 | 19.83 | 38.99 | 52.9 | 186.18 |
| F | 65 | 450.7 | 73.6 | 32.19 | 55.67 | 13.22 | 83.31 | 153.63 | 47.73 | 253.7 | 60.66 | 52.02 | 52.16 | 110.33 | 168.41 |
| F | 56 | 417.34 | 113.29 | 35.5 | 81.56 | 18.98 | 128.11 | 209.93 | 63.9 | 322.14 | 73.07 | 52.82 | 51.88 | 104.34 | 154.07 |
| F | 57 | 484.71 | 111.27 | 22.81 | 34.26 | 7.42 | 97.2 | 186.64 | 36.89 | 281.37 | 115.68 | 36.83 | 31.19 | 109.54 | 142.25 |
| F | 55 | 426.77 | 91.32 | 28.68 | 66.82 | 11.01 | 54.2 | 108.42 | 43.87 | 182.49 | 80.89 | 33.83 | 43.72 | 128.31 | 166.1 |
| F | 63 | 517.62 | 74.88 | 31.54 | 60.8 | 14.22 | 37.33 | 81.43 | 46.59 | 233.67 | 51.8 | 61.43 | 51.18 | 96.46 | 248.62 |
| F | 74 | 267.23 | 59.98 | 27.48 | 50.01 | 11.06 | 56.72 | 101.7 | 34.04 | 161.65 | 29.91 | 30.52 | 38.51 | 69.39 | 129.17 |
| F | 63 | 480.9 | 65.09 | 29.56 | 67.8 | 12.92 | 69.36 | 140.85 | 50.37 | 234.97 | 56.98 | 42.84 | 35.58 | 139.58 | 168.98 |
| F | 77 | 598.92 | 70.13 | 26.93 | 47.09 | 11.44 | 63.19 | 129.51 | 41.62 | 216.78 | 61.35 | 56.23 | 32.16 | 86.67 | 217.41 |
| F | 61 | 376.21 | 63.87 | 25.95 | 43.1 | 10.48 | 65.38 | 126.51 | 36.81 | 217.17 | 51.46 | 36.34 | 29.1 | 85.98 | 218.43 |
| F | 77 | 228.86 | 47.18 | 19.46 | 41.44 | 8.81 | 38.61 | 75.7 | 29.48 | 126.86 | 17.95 | 34.75 | 54.76 | 37.02 | 91.87 |
| F | 60 | 365.69 | 50.92 | 30.2 | 48.79 | 10.42 | 58.74 | 112.07 | 36.34 | 170.3 | 32.21 | 33.36 | 65.83 | 67.77 | 122.62 |
| F | 66 | 502.64 | 45.35 | 30.89 | 49.87 | 10.31 | 38.57 | 74.61 | 35.46 | 137.14 | 39.34 | 31.02 | 59.25 | 72.36 | 157.71 |
| F | 76 | 400.47 | 57.15 | 34.82 | 57.66 | 11.54 | 51.71 | 111.15 | 37.3 | 175.4 | 41.43 | 32.91 | 78.93 | 78.42 | 126.02 |
| F | 69 | 423.85 | 65.27 | 37.84 | 56.76 | 14.94 | 70.6 | 115.22 | 44.71 | 153.93 | 26.49 | 47.87 | 69.74 | 75.71 | 166.81 |
| F | 62 | 481.08 | 81.86 | 44.19 | 66.22 | 17.34 | 78.64 | 136.86 | 52.76 | 182.21 | 24.29 | 59.76 | 91.15 | 72.1 | 191.57 |
| F | 80 | 471.19 | 74.72 | 47.05 | 92.16 | 14.32 | 66.2 | 122.21 | 38.91 | 219.63 | 29.71 | 40.86 | 91.79 | 59.31 | 200.97 |
| F | 65 | 474.35 | 126.09 | 42.57 | 77.69 | 21.7 | 146.82 | 243.24 | 109.42 | 312.37 | 55.17 | 90.12 | 76.55 | 116.08 | 117.42 |
| F | 63 | 253.66 | 64.33 | 36.03 | 71.15 | 8.83 | 51.61 | 115.24 | 34.18 | 177.16 | 28.99 | 33.81 | 72.78 | 12.36 | 113.89 |
| F | 63 | 343.5 | 53.12 | 36.86 | 54.72 | 11.02 | 37.7 | 85.15 | 35.32 | 133.15 | 40.25 | 38.88 | 85.36 | 119.04 | 187.13 |
| F | 68 | 300.61 | 63.11 | 39.37 | 67.58 | 12.54 | 52.46 | 91.77 | 34.67 | 165.28 | 40.05 | 37.07 | 80.7 | 91.92 | 194.09 |
| F | 55 | 359.31 | 51.94 | 36.32 | 70.51 | 11.85 | 45.39 | 86.21 | 29.94 | 145.03 | 33.13 | 29.84 | 74.23 | 62.24 | 198.14 |
| F | 75 | 492.32 | 84.37 | 42.45 | 66.87 | 14.97 | 85.1 | 157.86 | 50.99 | 287.86 | 45.16 | 51.09 | 80.38 | 117.05 | 139.2 |
| F | 61 | 394.74 | 57.16 | 36.15 | 89.52 | 16.29 | 39.1 | 68.65 | 39.69 | 137.67 | 23.94 | 35.12 | 61.34 | 45.66 | 157.99 |
| F | 60 | 448.44 | 71.15 | 40.93 | 79.68 | 14.19 | 54.4 | 110.84 | 39.09 | 179.85 | 52.63 | 36.85 | 81.64 | 95.91 | 199.69 |
| F | 72 | 333.95 | 59.13 | 37.78 | 70.16 | 11.44 | 49.81 | 106.92 | 36.48 | 183.05 | 35.96 | 41.23 | 106.3 | 83.76 | 126.09 |
| F | 58 | 346.83 | 67.63 | 37.43 | 71.07 | 13.29 | 49.13 | 109.12 | 51.6 | 181.39 | 48.71 | 41.48 | 88.92 | 101.72 | 123.77 |
| F | 59 | 319.07 | 56.39 | 36.56 | 61.61 | 11.75 | 40.85 | 85.01 | 33.51 | 148.55 | 33.58 | 33.89 | 84.7 | 87.72 | 122.52 |
| F | 68 | 340.19 | 70.17 | 51.34 | 85.46 | 14.37 | 50.93 | 107.23 | 49.13 | 187.61 | 34 | 50.32 | 119.09 | 54.86 | 234.46 |
| Total | Mean±SD | 420.14±134.7 | 75.94±24.83 | 38±13.48 | 73.67±28 | 14.41±5.61 | 72.97±31.77 | 133.26±45.17 | 46.12±14.47 | 208.88±54.82 | 43.86±19.14 | 43.42±14.15 | 66.3±33.83 | 86.5±36.84 | 172.06±55.45 |

Table S12 Content of 13 D-amino acids in serum of healthy volunteers

| Sex | Age | D-Ala | D-Phe | D-Asn | D-Thr | D-Met | D-Ile | D-Leu | D-Tyr | D-Val | D-Asp | D-Trp | D-His | D-Glu |
| --- | --- | --- | --- | --- | --- | --- | --- | --- | --- | --- | --- | --- | --- | --- |
|  |  | (μM) | (μM) | (μM) | (μM) | (μM) | (μM) | (μM) | (μM) | (μM) | (μM) | (μM) | (μM) | (μM) |
| M | 79 | 5.20 | 0.79 | 0.66 | 0.39 | 0.43 | 1.11 | 2.59 | 0.41 | 1.64 | 0.42 | 0.28 | 0.79 | 0.47 |
| M | 69 | 5.48 | 0.61 | 0.69 | 0.34 | 0.35 | 1.04 | 1.76 | 0.64 | 1.53 | 0.57 | 0.67 | 0.67 | 0.22 |
| M | 67 | 3.16 | 0.46 | 0.85 | 0.46 | 0.25 | 0.71 | 1.32 | 0.59 | 1.18 | 0.55 | 0.58 | 0.91 | 0.17 |
| M | 64 | 2.95 | 0.66 | 0.55 | 0.47 | 0.32 | 1.37 | 2.11 | 0.50 | 1.34 | 0.49 | 0.20 | 0.84 | 0.08 |
| M | 61 | 1.23 | 0.86 | 0.66 | 0.28 | 0.23 | 0.77 | 1.72 | 0.71 | 1.54 | 0.66 | 0.81 | 0.55 | 0.40 |
| M | 68 | 3.37 | 0.64 | 0.63 | 0.31 | 0.36 | 0.97 | 2.03 | 0.77 | 1.82 | 0.66 | 0.76 | 0.82 | 0.26 |
| M | 69 | 5.42 | 0.80 | 0.47 | 0.33 | 0.24 | 0.90 | 1.84 | 0.62 | 1.43 | 0.85 | 0.62 | 0.86 | 0.46 |
| M | 62 | 3.31 | 0.60 | 0.60 | 0.29 | 0.32 | 0.73 | 1.31 | 0.61 | 1.36 | 0.50 | 0.55 | 0.80 | 0.22 |
| M | 70 | 4.50 | 0.49 | 0.68 | 0.25 | 0.22 | 0.72 | 1.67 | 0.54 | 1.33 | 0.51 | 0.59 | 0.79 | 0.21 |
| M | 59 | 3.65 | 0.83 | 0.47 | 0.29 | 0.17 | 0.67 | 1.47 | 0.47 | 1.31 | 0.72 | 0.37 | 0.62 | 0.22 |
| M | 65 | 1.26 | 0.40 | 0.34 | 0.34 | 0.16 | 0.49 | 1.15 | 0.48 | 1.02 | 0.81 | 0.48 | 0.66 | 0.16 |
| M | 55 | 1.46 | 0.38 | 0.35 | 0.30 | 0.14 | 0.61 | 1.23 | 0.36 | 1.09 | 0.33 | 0.51 | 0.54 | 0.10 |
| M | 58 | 2.12 | 0.46 | 0.52 | 0.23 | 0.17 | 0.69 | 1.46 | 0.41 | 1.26 | 0.42 | 0.54 | 0.85 | 0.13 |
| M | 63 | 1.37 | 0.50 | 0.44 | 0.27 | 0.18 | 0.62 | 1.28 | 0.43 | 1.17 | 0.44 | 0.54 | 0.66 | 0.10 |
| M | 55 | 1.95 | 0.63 | 0.52 | 0.25 | 0.19 | 0.94 | 1.82 | 0.46 | 1.43 | 0.62 | 0.40 | 0.80 | 0.42 |
| M | 63 | 1.92 | 0.71 | 0.36 | 0.25 | 0.25 | 1.61 | 3.00 | 0.48 | 1.70 | 0.50 | 0.54 | 0.23 | 0.22 |
| M | 77 | 1.49 | 0.91 | 0.53 | 0.29 | 0.19 | 1.10 | 1.80 | 0.24 | 0.88 | 0.48 | 0.32 | 0.15 | 0.08 |
| M | 59 | 1.58 | 0.68 | 0.48 | 0.29 | 0.22 | 1.17 | 2.02 | 0.36 | 1.44 | 0.65 | 0.81 | 0.17 | 0.47 |
| M | 67 | 2.84 | 0.60 | 0.57 | 0.28 | 0.25 | 1.30 | 2.15 | 0.38 | 1.46 | 0.59 | 0.56 | 0.13 | 0.42 |
| M | 62 | 5.37 | 0.82 | 0.84 | 0.33 | 0.23 | 1.78 | 2.92 | 0.38 | 1.71 | 1.26 | 0.35 | 0.19 | 0.36 |
| M | 67 | 3.14 | 0.66 | 0.63 | 0.30 | 0.29 | 1.35 | 2.49 | 0.42 | 1.28 | 0.51 | 0.60 | 0.14 | 0.28 |
| M | 70 | 3.49 | 0.92 | 1.00 | 0.27 | 0.29 | 1.49 | 3.31 | 0.48 | 1.66 | 1.72 | 0.51 | 0.14 | 0.58 |
| M | 83 | 3.20 | 0.72 | 0.78 | 0.28 | 0.28 | 1.45 | 3.09 | 0.70 | 2.30 | 1.08 | 0.82 | 0.13 | 0.46 |
| M | 58 | 2.83 | 0.94 | 0.45 | 0.22 | 0.11 | 0.92 | 1.85 | 0.24 | 1.11 | 0.56 | 0.20 | 0.12 | 0.37 |
| M | 62 | 1.03 | 0.84 | 0.51 | 0.28 | 0.16 | 0.99 | 2.39 | 0.33 | 1.43 | 1.17 | 0.57 | 0.12 | 0.51 |
| M | 67 | 3.46 | 0.54 | 0.43 | 0.23 | 0.21 | 1.23 | 2.29 | 0.25 | 1.25 | 0.66 | 0.60 | 0.14 | 0.22 |
| M | 70 | 3.23 | 0.77 | 0.60 | 0.46 | 0.28 | 1.59 | 2.31 | 0.47 | 1.68 | 0.97 | 0.79 | 0.32 | 0.39 |
| M | 83 | 2.94 | 0.47 | 0.51 | 0.26 | 0.17 | 0.60 | 1.39 | 0.45 | 1.32 | 0.80 | 0.55 | 0.16 | 0.33 |
| M | 58 | 2.37 | 0.42 | 0.47 | 0.26 | 0.13 | 0.74 | 1.74 | 0.49 | 1.39 | 0.45 | 0.55 | 0.16 | 0.21 |
| M | 61 | 1.56 | 0.60 | 0.66 | 0.29 | 0.29 | 1.26 | 2.02 | 0.57 | 1.72 | 0.49 | 0.67 | 0.18 | 0.18 |
| M | 66 | 5.72 | 0.74 | 0.52 | 0.43 | 0.30 | 0.86 | 1.58 | 0.70 | 1.17 | 0.42 | 0.33 | 0.32 | 0.22 |
| M | 69 | 5.33 | 0.76 | 0.56 | 0.24 | 0.07 | 1.60 | 2.22 | 0.48 | 1.84 | 0.93 | 0.39 | 0.19 | 0.69 |
| M | 67 | 3.20 | 1.12 | 0.62 | 0.46 | 0.07 | 1.19 | 2.47 | 0.74 | 2.02 | 1.20 | 0.97 | 0.22 | 0.43 |
| M | 63 | 3.76 | 0.87 | 0.74 | 0.45 | 0.08 | 1.21 | 2.27 | 0.66 | 1.85 | 0.88 | 0.69 | 0.32 | 0.45 |
| M | 62 | 2.56 | 1.09 | 0.60 | 0.31 | 0.09 | 1.50 | 2.64 | 0.69 | 2.13 | 1.79 | 0.33 | 0.31 | 0.43 |
| M | 79 | 1.70 | 0.43 | 0.45 | 0.15 | 0.18 | 0.76 | 0.92 | 0.47 | 0.92 | 0.52 | 0.61 | 0.21 | 0.40 |
| M | 80 | 5.66 | 0.91 | 0.48 | 0.29 | 0.33 | 0.80 | 1.57 | 0.73 | 0.92 | 0.49 | 0.20 | 0.30 | 0.33 |
| M | 76 | 1.35 | 0.27 | 0.35 | 0.26 | 0.15 | 0.21 | 0.52 | 0.45 | 0.41 | 0.22 | 0.38 | 0.26 | 0.26 |
| M | 75 | 2.09 | 0.40 | 0.45 | 0.27 | 0.15 | 0.30 | 0.79 | 0.56 | 0.64 | 0.41 | 0.45 | 0.13 | 0.23 |
| M | 70 | 4.16 | 0.61 | 0.53 | 0.33 | 0.19 | 1.18 | 2.11 | 0.35 | 1.14 | 0.85 | 0.70 | 0.22 | 0.38 |
| M | 71 | 3.40 | 0.65 | 0.57 | 0.29 | 0.23 | 1.63 | 2.42 | 0.37 | 1.50 | 0.99 | 0.64 | 0.31 | 0.44 |
| F | 69 | 1.71 | 0.51 | 0.76 | 0.32 | 0.16 | 1.22 | 2.16 | 0.17 | 0.87 | 0.78 | 0.39 | 0.37 | 0.35 |
| F | 65 | 5.41 | 0.83 | 0.45 | 0.29 | 0.22 | 1.82 | 2.86 | 0.49 | 1.79 | 1.25 | 0.79 | 0.55 | 0.75 |
| F | 56 | 2.35 | 1.42 | 0.68 | 0.29 | 0.36 | 2.57 | 4.16 | 0.64 | 2.50 | 1.69 | 0.67 | 0.34 | 0.90 |
| F | 57 | 2.35 | 1.38 | 0.90 | 0.26 | 0.15 | 1.93 | 3.35 | 0.43 | 2.13 | 2.08 | 0.41 | 0.37 | 0.80 |
| F | 55 | 4.20 | 1.25 | 0.75 | 0.41 | 0.21 | 1.32 | 2.36 | 0.52 | 1.48 | 1.62 | 0.58 | 0.26 | 0.74 |
| F | 63 | 3.87 | 0.80 | 0.75 | 0.29 | 0.25 | 1.45 | 2.52 | 0.45 | 1.68 | 1.11 | 0.86 | 0.32 | 0.55 |
| F | 74 | 1.11 | 0.65 | 0.50 | 0.16 | 0.33 | 1.03 | 1.80 | 0.34 | 1.12 | 0.58 | 0.42 | 0.34 | 0.94 |
| F | 63 | 4.55 | 0.70 | 0.49 | 0.30 | 0.21 | 1.18 | 2.41 | 0.50 | 1.69 | 1.17 | 0.66 | 0.26 | 0.87 |
| F | 77 | 6.89 | 0.89 | 0.55 | 0.44 | 0.21 | 1.17 | 2.48 | 0.46 | 1.69 | 1.28 | 0.95 | 0.31 | 0.66 |
| F | 61 | 2.59 | 0.77 | 0.41 | 0.30 | 0.18 | 1.44 | 2.10 | 0.41 | 1.67 | 1.06 | 0.55 | 0.20 | 0.49 |
| F | 77 | 2.80 | 0.35 | 0.24 | 0.25 | 0.11 | 0.37 | 0.84 | 0.29 | 0.73 | 0.26 | 0.40 | 0.35 | 0.44 |
| F | 60 | 4.15 | 0.36 | 0.33 | 0.20 | 0.13 | 0.61 | 1.20 | 0.39 | 1.04 | 0.42 | 0.31 | 0.57 | 0.12 |
| F | 66 | 4.94 | 0.34 | 0.49 | 0.24 | 0.14 | 0.42 | 0.85 | 0.37 | 0.83 | 0.50 | 0.33 | 0.37 | 0.12 |
| F | 76 | 3.17 | 0.43 | 0.37 | 0.27 | 0.15 | 0.52 | 1.20 | 0.36 | 1.04 | 0.49 | 0.35 | 0.54 | 0.07 |
| F | 69 | 3.66 | 0.50 | 0.55 | 0.24 | 0.20 | 0.70 | 1.33 | 0.48 | 0.99 | 0.39 | 0.48 | 0.41 | 0.07 |
| F | 62 | 4.54 | 0.70 | 0.73 | 0.28 | 0.23 | 0.87 | 1.62 | 0.60 | 1.10 | 0.51 | 0.65 | 0.56 | 0.13 |
| F | 80 | 2.32 | 0.70 | 0.65 | 0.37 | 0.21 | 0.74 | 1.55 | 0.48 | 1.54 | 0.50 | 0.42 | 0.74 | 0.15 |
| F | 65 | 4.77 | 0.98 | 0.62 | 0.37 | 0.29 | 1.47 | 2.61 | 0.37 | 2.04 | 0.68 | 0.87 | 0.46 | 0.27 |
| F | 63 | 1.78 | 0.50 | 0.45 | 0.36 | 0.12 | 0.59 | 1.32 | 0.38 | 1.19 | 0.45 | 0.38 | 0.51 | 0.10 |
| F | 63 | 1.38 | 0.40 | 0.43 | 0.26 | 0.14 | 0.40 | 0.92 | 0.34 | 0.84 | 0.52 | 0.47 | 0.62 | 0.23 |
| F | 68 | 3.42 | 0.54 | 0.42 | 0.33 | 0.20 | 0.59 | 1.08 | 0.42 | 1.13 | 0.53 | 0.44 | 0.60 | 0.23 |
| F | 55 | 3.08 | 0.38 | 0.56 | 0.36 | 0.15 | 0.49 | 0.93 | 0.33 | 0.89 | 0.43 | 0.33 | 0.43 | 0.05 |
| F | 75 | 5.67 | 0.69 | 0.58 | 0.29 | 0.20 | 0.90 | 1.78 | 0.54 | 1.82 | 0.62 | 0.53 | 0.51 | 0.36 |
| F | 61 | 2.86 | 0.46 | 0.63 | 0.42 | 0.23 | 0.43 | 0.82 | 0.43 | 0.85 | 0.39 | 0.42 | 0.39 | 0.40 |
| F | 60 | 3.78 | 0.52 | 0.38 | 0.36 | 0.18 | 0.55 | 1.15 | 0.43 | 1.09 | 0.61 | 0.37 | 0.53 | 0.06 |
| F | 72 | 3.33 | 0.41 | 0.36 | 0.31 | 0.15 | 0.52 | 1.18 | 0.36 | 1.12 | 0.49 | 0.45 | 0.74 | 0.18 |
| F | 58 | 1.39 | 0.53 | 0.52 | 0.28 | 0.18 | 0.53 | 1.22 | 0.53 | 1.12 | 0.66 | 0.46 | 0.67 | 0.22 |
| F | 59 | 2.20 | 0.41 | 0.49 | 0.28 | 0.15 | 0.46 | 0.98 | 0.41 | 0.97 | 0.52 | 0.39 | 0.65 | 0.12 |
| F | 68 | 1.75 | 0.52 | 0.46 | 0.30 | 0.19 | 0.56 | 1.18 | 0.53 | 1.16 | 0.50 | 0.57 | 0.82 | 0.08 |
| Total | Mean±SD | 3.17±1.41 | 0.66±0.24 | 0.55±0.15 | 0.31±0.07 | 0.21±0.08 | 0.99±0.46 | 1.84±0.72 | 0.47±0.13 | 1.36±0.41 | 0.73±0.39 | 0.53±0.18 | 0.43±0.24 | 0.33±0.22 |

Table S13 Content of 14 L-amino acids in patients with colorectal cancer

| Sex | Age | stage | L-Ala | L-Phe | L-Asn | L-Thr | L-Met | L-Ile | L-Leu | L-Tyr | L-Val | L-Asp | L-Trp | L-His | L-Glu | Gly |
| --- | --- | --- | --- | --- | --- | --- | --- | --- | --- | --- | --- | --- | --- | --- | --- | --- |
|  |  |  | (μM) | (μM) | (μM) | (μM) | (μM) | (μM) | (μM) | (μM) | (μM) | (μM) | (μM) | (μM) | (μM) | (μM) |
| M | 39 | II | 398.35 | 112.25 | 51.02 | 86.51 | 19.69 | 92.17 | 203.63 | 50.10 | 249.17 | 85.47 | 44.44 | 42.69 | 208.95 | 557.35 |
| M | 50 | II | 393.62 | 110.86 | 61.02 | 87.24 | 24.53 | 94.43 | 249.06 | 63.85 | 273.94 | 72.40 | 56.29 | 43.42 | 131.12 | 608.32 |
| M | 58 | II | 394.00 | 112.97 | 26.66 | 86.94 | 10.20 | 95.08 | 69.94 | 25.48 | 136.42 | 25.75 | 21.34 | 30.18 | 65.46 | 309.94 |
| M | 62 | II | 403.10 | 111.88 | 66.63 | 88.34 | 21.70 | 92.46 | 260.57 | 36.97 | 225.01 | 65.52 | 59.93 | 56.27 | 136.78 | 569.76 |
| M | 65 | II | 406.39 | 116.39 | 38.26 | 90.41 | 5.88 | 94.31 | 53.71 | 26.82 | 67.52 | 18.57 | 31.92 | 14.47 | 26.10 | 133.63 |
| M | 66 | II | 412.44 | 114.67 | 91.59 | 91.38 | 26.94 | 95.85 | 211.28 | 69.06 | 293.47 | 73.70 | 75.87 | 20.19 | 244.53 | 526.50 |
| M | 67 | II | 377.20 | 140.62 | 64.99 | 96.69 | 18.82 | 101.19 | 168.10 | 57.98 | 217.27 | 43.45 | 53.34 | 31.19 | 189.91 | 490.43 |
| M | 69 | II | 555.91 | 167.22 | 46.50 | 96.06 | 6.82 | 129.17 | 43.04 | 26.46 | 90.95 | 37.16 | 19.17 | 19.63 | 27.59 | 149.59 |
| M | 69 | II | 227.54 | 46.39 | 46.20 | 76.25 | 36.27 | 56.77 | 258.00 | 61.69 | 186.28 | 28.98 | 46.96 | 84.38 | 140.84 | 813.92 |
| M | 71 | II | 299.10 | 141.75 | 41.74 | 86.71 | 8.54 | 146.82 | 80.61 | 29.27 | 117.60 | 40.23 | 37.13 | 24.59 | 27.08 | 182.91 |
| M | 71 | II | 103.99 | 39.21 | 46.36 | 28.73 | 16.85 | 40.64 | 156.42 | 45.43 | 223.62 | 16.98 | 40.05 | 61.99 | 50.68 | 374.37 |
| M | 72 | II | 569.97 | 158.61 | 34.50 | 88.01 | 10.34 | 122.29 | 71.59 | 28.52 | 105.26 | 25.18 | 23.40 | 31.58 | 36.71 | 201.94 |
| M | 73 | II | 494.93 | 105.07 | 37.11 | 79.73 | 13.16 | 101.52 | 81.04 | 31.02 | 131.86 | 23.88 | 32.20 | 44.58 | 127.17 | 419.60 |
| M | 73 | II | 147.12 | 37.15 | 56.36 | 40.20 | 13.94 | 33.15 | 161.54 | 37.58 | 172.53 | 34.20 | 32.45 | 59.47 | 143.58 | 440.74 |
| M | 73 | II | 503.40 | 187.87 | 41.35 | 110.75 | 15.73 | 119.13 | 195.42 | 47.16 | 229.85 | 45.10 | 53.62 | 57.19 | 117.91 | 433.54 |
| M | 74 | II | 249.68 | 52.06 | 52.28 | 40.49 | 20.57 | 46.05 | 109.52 | 28.50 | 168.76 | 39.72 | 45.63 | 46.23 | 53.14 | 397.58 |
| M | 74 | II | 319.27 | 144.32 | 55.74 | 93.13 | 9.91 | 90.21 | 25.28 | 32.03 | 109.57 | 18.33 | 75.96 | 20.69 | 66.13 | 172.91 |
| M | 74 | II | 171.76 | 64.55 | 93.38 | 50.04 | 22.70 | 42.28 | 88.82 | 44.46 | 196.80 | 43.96 | 45.35 | 23.76 | 37.45 | 428.96 |
| M | 74 | II | 302.81 | 59.63 | 52.88 | 61.69 | 12.01 | 48.21 | 117.53 | 30.14 | 169.15 | 22.17 | 35.14 | 50.65 | 57.55 | 313.65 |
| M | 74 | II | 276.36 | 90.18 | 51.75 | 64.77 | 17.33 | 94.39 | 101.21 | 47.58 | 232.00 | 92.20 | 44.55 | 48.39 | 35.25 | 653.96 |
| M | 75 | II | 367.28 | 97.57 | 48.00 | 77.15 | 6.48 | 114.00 | 127.24 | 27.22 | 73.90 | 66.32 | 15.43 | 63.11 | 101.32 | 209.93 |
| M | 77 | II | 329.09 | 109.16 | 50.14 | 85.72 | 8.94 | 69.06 | 38.76 | 29.17 | 146.61 | 47.22 | 33.03 | 24.41 | 15.15 | 366.44 |
| M | 77 | II | 263.30 | 40.66 | 57.16 | 27.74 | 16.62 | 90.78 | 101.99 | 37.83 | 166.95 | 51.87 | 30.34 | 18.73 | 92.54 | 265.45 |
| M | 82 | II | 216.66 | 71.34 | 35.10 | 43.01 | 14.90 | 54.16 | 112.64 | 29.18 | 149.91 | 28.79 | 46.18 | 31.19 | 55.97 | 334.15 |
| M | 82 | II | 364.48 | 95.55 | 25.25 | 109.35 | 7.99 | 67.16 | 68.30 | 27.14 | 138.81 | 39.67 | 23.57 | 39.10 | 31.75 | 283.91 |
| M | 84 | II | 287.18 | 67.81 | 27.00 | 101.47 | 10.18 | 66.52 | 89.69 | 26.25 | 160.34 | 29.06 | 22.86 | 33.22 | 60.26 | 335.46 |
| M | 84 | II | 443.57 | 154.44 | 34.32 | 128.74 | 11.81 | 94.60 | 105.06 | 30.25 | 136.88 | 12.75 | 21.28 | 28.12 | 31.10 | 277.52 |
| M | 85 | II | 141.06 | 24.66 | 34.89 | 38.66 | 11.58 | 32.34 | 74.35 | 30.80 | 116.66 | 34.92 | 28.66 | 45.32 | 86.38 | 385.31 |
| F | 52 | II | 390.21 | 86.04 | 39.92 | 59.39 | 21.05 | 49.24 | 105.86 | 42.71 | 166.75 | 29.10 | 53.65 | 41.60 | 56.67 | 185.18 |
| F | 53 | II | 191.08 | 79.79 | 57.01 | 71.84 | 13.14 | 73.69 | 137.19 | 42.45 | 134.31 | 26.55 | 46.08 | 41.91 | 23.99 | 292.17 |
| F | 54 | II | 246.34 | 94.93 | 56.85 | 57.31 | 16.37 | 58.50 | 116.03 | 42.28 | 201.37 | 28.55 | 32.96 | 51.34 | 75.87 | 618.67 |
| F | 58 | II | 204.20 | 74.75 | 48.77 | 37.26 | 9.46 | 40.02 | 183.76 | 26.11 | 62.82 | 10.42 | 22.34 | 75.21 | 133.11 | 389.62 |
| F | 63 | II | 181.65 | 42.46 | 57.47 | 56.73 | 22.53 | 91.71 | 44.13 | 52.84 | 255.73 | 52.35 | 52.79 | 29.61 | 50.72 | 566.90 |
| F | 67 | II | 227.17 | 62.93 | 43.52 | 56.10 | 25.70 | 39.81 | 173.17 | 63.48 | 193.83 | 46.86 | 54.12 | 91.55 | 226.13 | 561.34 |
| F | 67 | II | 185.49 | 53.00 | 31.19 | 58.87 | 6.61 | 78.77 | 156.83 | 36.14 | 182.48 | 33.94 | 21.10 | 60.64 | 150.68 | 603.02 |
| F | 68 | II | 269.34 | 74.47 | 49.59 | 59.03 | 24.21 | 81.02 | 106.84 | 68.10 | 251.22 | 45.48 | 50.64 | 55.07 | 65.43 | 465.14 |
| F | 68 | II | 223.29 | 92.15 | 71.07 | 98.89 | 8.71 | 67.68 | 179.81 | 29.41 | 121.63 | 73.97 | 25.62 | 65.51 | 131.52 | 226.94 |
| F | 69 | II | 304.18 | 83.39 | 43.30 | 95.40 | 13.58 | 102.85 | 78.50 | 31.07 | 101.97 | 53.55 | 29.92 | 31.29 | 205.45 | 377.94 |
| F | 71 | II | 227.52 | 44.35 | 31.46 | 39.52 | 9.83 | 25.78 | 78.71 | 33.19 | 124.30 | 14.02 | 26.77 | 33.30 | 176.08 | 216.77 |
| F | 73 | II | 441.81 | 122.03 | 50.17 | 96.00 | 12.55 | 105.81 | 70.85 | 34.67 | 121.29 | 38.96 | 35.65 | 32.82 | 36.68 | 258.83 |
| F | 74 | II | 466.38 | 159.46 | 40.65 | 84.77 | 8.74 | 66.47 | 90.53 | 34.23 | 81.93 | 24.72 | 19.05 | 29.33 | 169.31 | 221.95 |
| F | 79 | II | 287.90 | 62.69 | 48.83 | 126.25 | 7.67 | 73.01 | 45.06 | 28.93 | 107.72 | 64.49 | 32.25 | 26.62 | 65.09 | 161.84 |
| F | 84 | II | 384.20 | 138.46 | 53.13 | 159.66 | 22.44 | 84.52 | 77.10 | 69.18 | 290.48 | 60.37 | 62.92 | 20.31 | 104.87 | 450.37 |
| M | 50 | III | 191.90 | 57.51 | 42.61 | 46.85 | 13.05 | 51.38 | 228.88 | 42.35 | 184.14 | 67.68 | 49.45 | 70.67 | 227.92 | 409.70 |
| M | 58 | III | 348.19 | 66.69 | 38.53 | 54.39 | 11.95 | 43.14 | 153.65 | 30.26 | 114.27 | 32.39 | 27.43 | 49.88 | 52.59 | 233.35 |
| M | 61 | III | 188.25 | 52.82 | 30.46 | 64.60 | 2.68 | 31.58 | 79.71 | 29.05 | 55.41 | 12.32 | 35.03 | 37.12 | 96.07 | 147.98 |
| M | 69 | III | 271.64 | 82.50 | 102.85 | 41.90 | 11.26 | 46.88 | 54.98 | 28.63 | 115.40 | 62.10 | 39.44 | 14.51 | 30.27 | 186.09 |
| M | 69 | III | 151.64 | 46.99 | 46.11 | 49.83 | 30.83 | 30.67 | 87.22 | 98.96 | 123.94 | 35.22 | 46.70 | 29.42 | 168.00 | 360.35 |
| M | 70 | III | 157.62 | 64.05 | 42.11 | 32.37 | 13.22 | 40.74 | 145.13 | 27.79 | 159.75 | 25.87 | 28.54 | 46.45 | 37.86 | 246.56 |
| M | 70 | III | 487.61 | 117.04 | 43.85 | 101.53 | 29.31 | 130.64 | 102.61 | 64.53 | 319.59 | 29.28 | 62.01 | 35.34 | 85.83 | 319.30 |
| M | 71 | III | 308.09 | 89.75 | 32.17 | 75.41 | 7.56 | 88.47 | 209.27 | 35.33 | 79.02 | 29.10 | 23.26 | 55.82 | 71.87 | 210.50 |
| M | 72 | III | 192.42 | 47.60 | 55.88 | 65.09 | 11.94 | 47.00 | 58.60 | 35.22 | 178.85 | 51.24 | 38.70 | 27.08 | 80.53 | 362.79 |
| M | 72 | III | 89.51 | 15.05 | 38.26 | 14.27 | 18.48 | 35.77 | 131.96 | 33.28 | 132.16 | 36.11 | 40.11 | 35.41 | 133.26 | 303.84 |
| M | 73 | III | 227.73 | 73.98 | 94.98 | 50.56 | 8.08 | 41.14 | 70.33 | 38.25 | 91.14 | 57.47 | 21.15 | 42.21 | 84.75 | 163.63 |
| M | 73 | III | 450.86 | 95.46 | 49.83 | 93.23 | 13.86 | 150.18 | 77.16 | 32.55 | 220.46 | 48.80 | 39.05 | 25.20 | 117.95 | 333.86 |
| M | 74 | III | 242.73 | 59.95 | 39.51 | 52.68 | 12.52 | 62.53 | 143.47 | 31.38 | 124.01 | 27.50 | 37.24 | 45.22 | 152.54 | 264.48 |
| M | 74 | III | 331.01 | 107.67 | 41.95 | 97.55 | 43.86 | 139.92 | 76.73 | 32.12 | 167.50 | 50.51 | 34.62 | 36.14 | 81.54 | 501.79 |
| F | 37 | III | 154.04 | 39.74 | 35.11 | 40.12 | 9.02 | 38.44 | 151.59 | 25.99 | 133.72 | 20.50 | 26.02 | 52.92 | 113.34 | 245.70 |
| F | 48 | III | 260.66 | 91.84 | 94.17 | 74.70 | 10.07 | 68.04 | 67.53 | 32.04 | 156.27 | 47.94 | 42.16 | 35.33 | 42.08 | 142.69 |
| F | 59 | III | 316.90 | 73.29 | 74.13 | 112.63 | 19.41 | 54.55 | 124.11 | 44.99 | 208.51 | 75.38 | 34.96 | 28.13 | 82.42 | 434.70 |
| F | 61 | III | 125.64 | 57.41 | 60.16 | 59.95 | 18.53 | 43.56 | 141.00 | 37.65 | 139.76 | 14.69 | 25.10 | 20.11 | 78.75 | 389.86 |
| F | 66 | III | 262.73 | 75.62 | 27.23 | 61.42 | 14.69 | 89.28 | 103.18 | 26.86 | 149.20 | 12.20 | 25.50 | 32.09 | 36.21 | 226.09 |
| F | 68 | III | 212.48 | 62.21 | 32.80 | 55.44 | 6.74 | 53.37 | 90.05 | 34.33 | 108.30 | 19.76 | 22.71 | 37.12 | 38.55 | 233.42 |
| F | 69 | III | 296.91 | 144.21 | 37.02 | 107.11 | 7.97 | 110.35 | 62.65 | 40.25 | 112.93 | 14.99 | 25.14 | 24.19 | 52.09 | 209.28 |
| F | 73 | III | 168.57 | 38.58 | 87.31 | 61.16 | 4.82 | 53.43 | 80.62 | 43.25 | 57.95 | 58.01 | 22.72 | 32.60 | 32.13 | 100.33 |
| F | 76 | III | 136.25 | 78.77 | 59.45 | 59.98 | 5.29 | 68.52 | 40.96 | 30.55 | 150.91 | 17.04 | 19.93 | 18.00 | 82.97 | 457.26 |
| F | 78 | III | 461.05 | 127.07 | 66.61 | 77.47 | 27.77 | 76.67 | 125.55 | 47.25 | 200.17 | 27.18 | 24.99 | 23.76 | 82.97 | 693.97 |
| F | 82 | III | 201.33 | 53.07 | 41.25 | 58.22 | 20.58 | 75.56 | 81.20 | 50.22 | 130.21 | 20.03 | 35.33 | 20.13 | 96.43 | 234.52 |
| Mean±SD | 68.69±10.41 |  | 292.44±115.86 | 86.24±38.23 | 50.08±17.67 | 72.37±27.49 | 14.95±7.89 | 74.05±30.99 | 114.07±56.11 | 39.2±14.03 | 157.33±59.92 | 39.12±19.65 | 36.61±13.79 | 39.05±16.76 | 91.6±56.96 | 347.74±156.09 |

Table S14 Content of 13 D-amino acids in patients with colorectal cancer

| Sex | Age | Stage | D-Ala | D-Phe | D-Asn | D-Thr | D-Met | D-Ile | D-Leu | D-Tyr | D-Val | D-Asp | D-Trp | D-His | D-Glu |
| --- | --- | --- | --- | --- | --- | --- | --- | --- | --- | --- | --- | --- | --- | --- | --- |
|  |  |  | (μM) | (μM) | (μM) | (μM) | (μM) | (μM) | (μM) | (μM) | (μM) | (μM) | (μM) | (μM) | (μM) |
| M | 39 | II | 4.08 | 1.25 | 1.56 | 0.45 | 0.28 | 1.04 | 2.16 | 0.53 | 1.64 | 1.05 | 0.55 | 0.38 | 0.24 |
| M | 50 | II | 7.80 | 1.38 | 1.86 | 0.24 | 0.43 | 1.05 | 2.88 | 0.60 | 1.54 | 1.06 | 0.55 | 0.32 | 0.36 |
| M | 58 | II | 3.73 | 0.37 | 0.80 | 0.39 | 0.16 | 0.66 | 0.80 | 0.26 | 0.82 | 0.36 | 0.27 | 0.28 | 0.29 |
| M | 62 | II | 3.08 | 1.34 | 1.01 | 0.24 | 0.39 | 1.36 | 3.10 | 0.41 | 1.43 | 0.68 | 0.73 | 0.67 | 0.10 |
| M | 65 | II | 1.57 | 0.26 | 0.45 | 0.24 | 0.09 | 0.49 | 0.59 | 0.19 | 0.50 | 0.25 | 0.45 | 0.23 | 0.10 |
| M | 66 | II | 8.26 | 1.87 | 2.08 | 0.22 | 0.58 | 1.11 | 3.58 | 0.92 | 2.09 | 0.95 | 1.08 | 0.20 | 0.33 |
| M | 67 | II | 7.91 | 1.05 | 1.37 | 0.25 | 0.36 | 1.04 | 2.40 | 0.73 | 1.50 | 0.74 | 0.69 | 0.27 | 0.54 |
| M | 69 | II | 1.23 | 0.31 | 0.28 | 0.10 | 0.13 | 0.38 | 0.50 | 0.16 | 0.63 | 0.18 | 0.27 | 0.21 | 0.53 |
| M | 69 | II | 6.41 | 1.73 | 0.83 | 0.40 | 0.55 | 1.17 | 2.52 | 0.62 | 1.00 | 0.37 | 0.51 | 0.92 | 0.48 |
| M | 71 | II | 4.08 | 0.51 | 0.49 | 0.07 | 0.16 | 0.56 | 1.06 | 0.40 | 0.87 | 0.28 | 0.58 | 0.23 | 0.30 |
| M | 71 | II | 5.75 | 1.66 | 1.06 | 0.39 | 0.47 | 1.09 | 2.10 | 0.54 | 1.80 | 0.43 | 0.50 | 0.18 | 0.07 |
| M | 72 | II | 2.19 | 0.61 | 0.55 | 0.06 | 0.19 | 0.46 | 0.93 | 0.31 | 0.72 | 0.32 | 0.32 | 0.29 | 0.08 |
| M | 73 | II | 4.03 | 0.39 | 0.86 | 0.22 | 0.20 | 0.46 | 0.85 | 0.32 | 0.77 | 0.41 | 0.34 | 0.38 | 0.09 |
| M | 73 | II | 5.22 | 0.76 | 1.34 | 0.26 | 0.27 | 1.03 | 1.94 | 0.42 | 1.12 | 0.58 | 0.36 | 0.50 | 0.06 |
| M | 73 | II | 4.90 | 0.89 | 1.64 | 0.36 | 0.24 | 1.26 | 2.18 | 0.54 | 1.67 | 0.63 | 0.69 | 0.51 | 0.34 |
| M | 74 | II | 4.32 | 1.11 | 0.40 | 0.57 | 0.46 | 0.76 | 1.34 | 0.33 | 1.00 | 0.47 | 0.64 | 0.52 | 0.25 |
| M | 74 | II | 2.96 | 0.65 | 0.60 | 0.37 | 0.09 | 0.68 | 1.13 | 0.46 | 0.86 | 0.25 | 1.31 | 0.23 | 0.59 |
| M | 74 | II | 4.69 | 1.08 | 0.84 | 0.29 | 0.53 | 0.74 | 1.55 | 0.55 | 1.28 | 0.36 | 0.66 | 0.61 | 0.43 |
| M | 74 | II | 5.67 | 0.77 | 0.90 | 0.49 | 0.30 | 0.83 | 1.34 | 0.41 | 1.21 | 0.27 | 0.49 | 0.61 | 0.20 |
| M | 74 | II | 2.16 | 1.79 | 1.66 | 0.60 | 0.52 | 1.03 | 1.74 | 0.55 | 1.51 | 0.19 | 0.49 | 0.69 | 0.06 |
| M | 75 | II | 2.20 | 0.18 | 0.45 | 0.11 | 0.13 | 0.40 | 0.52 | 0.21 | 0.50 | 0.16 | 0.22 | 0.25 | 0.35 |
| M | 77 | II | 7.05 | 0.80 | 1.21 | 0.22 | 0.17 | 0.56 | 1.29 | 0.36 | 1.09 | 0.75 | 0.45 | 0.16 | 0.06 |
| M | 77 | II | 3.39 | 0.96 | 0.83 | 0.26 | 0.53 | 0.80 | 1.56 | 0.40 | 1.12 | 0.35 | 0.44 | 0.50 | 0.40 |
| M | 82 | II | 5.84 | 0.90 | 0.39 | 0.35 | 0.31 | 0.61 | 0.68 | 0.24 | 0.96 | 0.36 | 0.63 | 0.59 | 0.09 |
| M | 82 | II | 2.15 | 0.86 | 0.51 | 0.08 | 0.18 | 0.51 | 1.22 | 0.37 | 1.14 | 0.41 | 0.37 | 0.29 | 0.29 |
| M | 84 | II | 2.96 | 0.47 | 0.67 | 0.10 | 0.25 | 1.19 | 1.43 | 0.33 | 1.19 | 0.22 | 0.32 | 0.39 | 0.27 |
| M | 84 | II | 3.89 | 0.63 | 0.61 | 0.15 | 0.33 | 0.46 | 0.88 | 0.23 | 0.85 | 0.38 | 0.26 | 0.47 | 0.07 |
| M | 85 | II | 2.49 | 0.44 | 0.65 | 0.17 | 0.18 | 0.88 | 1.22 | 0.40 | 0.79 | 0.38 | 0.35 | 0.44 | 0.24 |
| F | 52 | II | 2.74 | 0.86 | 0.67 | 0.23 | 0.41 | 1.15 | 1.90 | 0.75 | 1.44 | 0.33 | 0.97 | 0.41 | 0.16 |
| F | 53 | II | 1.11 | 0.72 | 0.92 | 0.29 | 0.19 | 0.66 | 1.17 | 0.48 | 0.89 | 0.36 | 0.56 | 0.46 | 0.32 |
| F | 54 | II | 2.91 | 0.74 | 1.43 | 0.36 | 0.25 | 1.06 | 2.06 | 0.52 | 1.38 | 0.54 | 0.32 | 0.45 | 0.10 |
| F | 58 | II | 3.28 | 0.37 | 0.57 | 0.08 | 0.15 | 0.33 | 0.60 | 0.35 | 0.42 | 0.20 | 0.34 | 0.43 | 0.37 |
| F | 63 | II | 5.46 | 1.22 | 1.68 | 0.32 | 0.44 | 1.15 | 1.88 | 0.63 | 1.67 | 0.88 | 0.59 | 0.94 | 0.06 |
| F | 67 | II | 3.66 | 1.54 | 1.28 | 0.29 | 0.40 | 0.65 | 1.81 | 0.64 | 1.18 | 0.80 | 0.63 | 0.67 | 0.89 |
| F | 67 | II | 2.29 | 0.64 | 0.80 | 0.46 | 0.13 | 0.87 | 1.32 | 0.44 | 1.25 | 0.54 | 0.26 | 0.81 | 0.44 |
| F | 68 | II | 6.97 | 1.34 | 1.41 | 0.67 | 0.42 | 0.87 | 2.00 | 0.74 | 1.65 | 0.64 | 0.60 | 0.53 | 0.12 |
| F | 68 | II | 1.73 | 0.55 | 0.38 | 0.37 | 0.17 | 0.53 | 0.97 | 0.26 | 0.82 | 0.30 | 0.34 | 0.20 | 0.35 |
| F | 69 | II | 5.45 | 0.64 | 1.14 | 0.20 | 0.21 | 0.48 | 1.05 | 0.37 | 0.67 | 0.65 | 0.41 | 0.39 | 0.56 |
| F | 71 | II | 2.61 | 0.45 | 0.94 | 0.25 | 0.17 | 0.37 | 0.90 | 0.40 | 0.79 | 0.25 | 0.38 | 0.33 | 0.52 |
| F | 73 | II | 3.64 | 0.65 | 0.49 | 0.22 | 0.20 | 0.51 | 1.10 | 0.39 | 0.76 | 0.17 | 0.48 | 0.42 | 0.26 |
| F | 74 | II | 1.38 | 0.36 | 0.40 | 0.31 | 0.15 | 0.36 | 0.53 | 0.29 | 0.56 | 0.13 | 0.27 | 0.39 | 0.49 |
| F | 79 | II | 1.53 | 0.69 | 0.38 | 0.19 | 0.13 | 0.59 | 1.08 | 0.44 | 0.93 | 0.43 | 0.54 | 0.33 | 0.18 |
| F | 84 | II | 2.00 | 0.97 | 1.09 | 0.64 | 0.34 | 1.41 | 2.47 | 0.72 | 1.94 | 0.36 | 0.69 | 0.80 | 0.19 |
| M | 50 | III | 3.34 | 0.98 | 1.11 | 0.52 | 0.24 | 1.16 | 1.94 | 0.58 | 1.53 | 0.52 | 0.71 | 0.77 | 0.49 |
| M | 58 | III | 3.50 | 0.36 | 0.60 | 0.39 | 0.19 | 0.53 | 0.97 | 0.34 | 0.79 | 0.20 | 0.34 | 0.51 | 0.42 |
| M | 61 | III | 1.83 | 0.13 | 0.25 | 0.05 | 0.06 | 0.12 | 0.27 | 0.16 | 0.10 | 0.18 | 0.18 | 0.19 | 0.38 |
| M | 69 | III | 2.77 | 0.67 | 0.62 | 0.22 | 0.18 | 0.49 | 1.11 | 0.37 | 0.84 | 0.39 | 0.53 | 0.49 | 0.49 |
| M | 69 | III | 4.12 | 0.68 | 0.96 | 0.36 | 0.27 | 0.53 | 1.23 | 0.47 | 0.94 | 0.34 | 0.86 | 0.77 | 0.53 |
| M | 70 | III | 2.95 | 0.50 | 0.63 | 0.29 | 0.25 | 0.70 | 1.19 | 0.32 | 1.07 | 0.23 | 0.34 | 0.49 | 0.11 |
| M | 70 | III | 3.39 | 1.08 | 1.18 | 0.59 | 0.54 | 1.75 | 2.97 | 0.78 | 2.35 | 0.35 | 0.74 | 0.85 | 0.11 |
| M | 71 | III | 2.21 | 0.40 | 0.61 | 0.19 | 0.13 | 0.51 | 0.82 | 0.33 | 0.57 | 0.25 | 0.35 | 0.39 | 0.33 |
| M | 72 | III | 4.27 | 1.17 | 0.95 | 0.48 | 0.24 | 0.97 | 1.94 | 0.52 | 1.49 | 0.47 | 0.63 | 0.59 | 0.32 |
| M | 72 | III | 5.48 | 0.81 | 0.57 | 0.70 | 0.38 | 0.71 | 0.90 | 0.39 | 1.02 | 0.31 | 0.58 | 0.72 | 0.54 |
| M | 73 | III | 1.24 | 0.49 | 0.58 | 0.36 | 0.13 | 0.53 | 1.01 | 0.25 | 0.66 | 0.31 | 0.31 | 0.44 | 0.19 |
| M | 73 | III | 4.19 | 0.70 | 0.78 | 0.29 | 0.31 | 0.89 | 1.71 | 0.37 | 1.29 | 0.34 | 0.40 | 0.62 | 0.43 |
| M | 74 | III | 4.32 | 0.81 | 0.62 | 0.29 | 0.30 | 0.75 | 1.21 | 0.44 | 1.05 | 0.28 | 0.65 | 0.56 | 0.36 |
| M | 74 | III | 3.89 | 1.40 | 0.54 | 0.49 | 0.79 | 1.03 | 1.65 | 0.34 | 0.96 | 0.61 | 0.34 | 0.25 | 0.12 |
| F | 37 | III | 2.53 | 0.40 | 0.59 | 0.32 | 0.18 | 0.65 | 0.93 | 0.33 | 1.02 | 0.25 | 0.33 | 0.54 | 0.09 |
| F | 48 | III | 1.51 | 0.70 | 0.70 | 0.30 | 0.16 | 0.77 | 1.61 | 0.41 | 1.12 | 0.27 | 0.52 | 0.37 | 0.38 |
| F | 59 | III | 1.83 | 1.95 | 1.66 | 0.34 | 0.56 | 0.78 | 2.88 | 0.68 | 1.38 | 0.86 | 0.44 | 0.37 | 0.05 |
| F | 61 | III | 2.29 | 0.66 | 0.86 | 0.30 | 0.34 | 1.08 | 1.50 | 0.60 | 1.26 | 0.35 | 0.58 | 0.39 | 1.18 |
| F | 66 | III | 3.04 | 0.50 | 0.58 | 0.20 | 0.23 | 0.60 | 1.11 | 0.32 | 1.08 | 0.27 | 0.19 | 0.43 | 0.14 |
| F | 68 | III | 2.12 | 0.48 | 0.64 | 0.06 | 0.14 | 0.60 | 0.92 | 0.22 | 0.84 | 0.36 | 0.29 | 0.51 | 0.35 |
| F | 69 | III | 1.41 | 0.41 | 0.60 | 0.16 | 0.12 | 0.57 | 1.09 | 0.29 | 0.87 | 0.27 | 0.37 | 0.50 | 0.15 |
| F | 73 | III | 1.31 | 0.31 | 0.26 | 0.23 | 0.12 | 0.27 | 0.46 | 0.22 | 0.41 | 0.50 | 0.25 | 0.26 | 0.13 |
| F | 76 | III | 3.78 | 0.78 | 1.17 | 0.72 | 0.10 | 0.99 | 1.69 | 0.40 | 1.13 | 0.30 | 0.27 | 0.30 | 0.26 |
| F | 78 | III | 3.56 | 1.26 | 1.25 | 0.18 | 0.33 | 0.72 | 1.87 | 0.50 | 1.02 | 0.54 | 0.22 | 0.25 | 0.07 |
| F | 82 | III | 2.01 | 0.83 | 0.81 | 0.58 | 0.21 | 0.76 | 0.91 | 0.50 | 1.00 | 0.20 | 0.35 | 0.08 | 0.10 |
| Total | Mean±SD |  | 3.52±1.75 | 0.81±0.43 | 0.86±0.42 | 0.31±0.16 | 0.28±0.15 | 0.77±0.31 | 1.44±0.7 | 0.43±0.16 | 1.08±0.41 | 0.42±0.22 | 0.48±0.21 | 0.45±0.19 | 0.29±0.21 |
